# Intermolecular oxidative decarbonylative [2 + 2 + 2] carbocyclization of *N*-(2-ethynylaryl)acrylamides with tertiary and secondary alkyl aldehydes involving C(sp^3^)–H functionalization†
†Electronic supplementary information (ESI) available. CCDC 1438591. For ESI and crystallographic data in CIF or other electronic format see DOI: 10.1039/c6sc02451c


**DOI:** 10.1039/c6sc02451c

**Published:** 2016-07-21

**Authors:** Yang Li, Gao-Hui Pan, Ming Hu, Bang Liu, Ren-Jie Song, Jin-Heng Li

**Affiliations:** a State Key Laboratory of Chemo/Biosensing and Chemometrics , Hunan University , Changsha 410082 , China . Email: srj0731@hnu.edu.cn ; Email: jhli@hnu.edu.cn; b Key Laboratory of Jiangxi Province for Persistent Pollutants Control and Resources Recycle , Nanchang Hangkong University , Nanchang 330063 , China; c State Key Laboratory of Applied Organic Chemistry , Lanzhou University , Lanzhou 730000 , China

## Abstract

A new metal-free oxidative decarbonylative [2 + 2 + 2] carbocyclization of *N*-(2-ethynylaryl)acrylamides with tertiary and secondary alkyl aldehydes is described. This reaction enables the formation of three new C–C bonds in a single reaction by a sequence of oxidative decarbonylation, radical addition across C–C unsaturated bonds, C–H functionalization and annulation, and represents the first oxidative decarbonylative [2 + 2 + 2] carbocyclization approach using tertiary and secondary alkyl aldehydes as a two carbon unit for assembling six-membered carbocycle-fused polycycles.

## Abstract 2

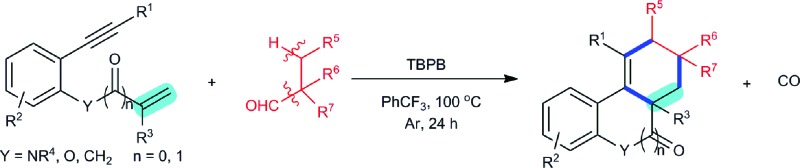
A new metal-free oxidative decarbonylative [2 + 2 + 2] carbocyclization of *N*-(2-ethynylaryl)acrylamides with tertiary and secondary alkyl aldehydes is described.

## Introduction

High-order carbocyclization reactions represent one of the most powerful methods for building complex carbocyclic frameworks. In this field, one particularly fascinating area is the intermolecular [2 + 2 + *m*] carbocyclization of 1,*n*-enynes, which continues to gain much attention due to its straightforward and highly atom-economic features.^1–4^ Significant achievements include the [2 + 2 + 2] carbocyclization strategy which can allow the formation of a six-membered carbocycle within a complex polycyclic system by introducing a two-carbon unit across 1,*n*-enynes.^2,3^ Despite these advances, there are still limitations with the available transformations, such as the requirement for noble metal catalysts and narrow two-carbon unit scope (*e.g.*, alkynes,^3a–e^ alkenes^3h–m^ and arylsulfonyl chlorides^3*n*^). Therefore, it would be welcomed to develop more efficient methods, especially metal-free use of new two-carbon unit strategies, to achieve the [2 + 2 + 2] carbocyclization of 1,*n*-enynes, which unfortunately remains a great challenge.

**Scheme 1:**
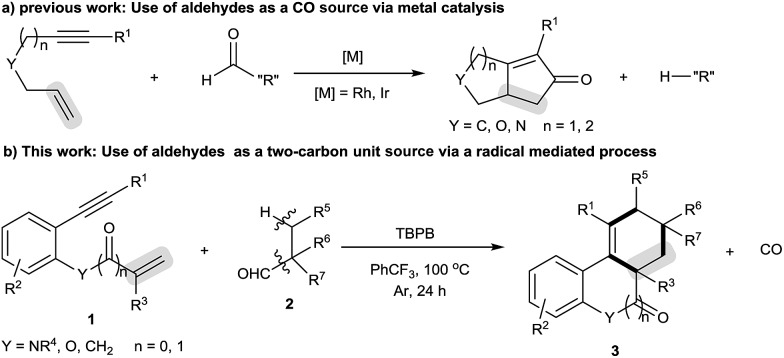
[2 + 2 + *m*] carbocyclization of 1,*n*-enynes with aldehydes.

The decarbonylation reaction has proven among the most important methods for the formation of diverse chemical bonds in synthesis, the majority of which focus on the cleavage of C–CHO bonds by extrusion of carbon monoxide (CO) gas and then transformation through radical^5^ and anionic intermediates.^6,7^ Such successful transformations include transition metal-catalyzed Pauson–Khand-type reactions of 1,*n*-enynes using aldehydes as the carbon monoxide (CO) source (Scheme 1a).^7^ To our knowledge, however, approaches for carbocyclization of 1,*n*-enynes with aldehydes as the “R” source have never been reported.^5*g*^ Intrigued by these results, we envisioned that extension of the decarbonylation concept to the combination of *N*-(2-ethynylaryl)acrylamides and alkyl aldehydes would offer a novel method to assemble six-membered carbocycle-fused polycyclic architectures. Herein, we report a novel oxidative radical decarbonylative [2 + 2 + 2] carbocyclization of *N*-(2-ethynylaryl)acrylamides with tertiary and secondary alkyl aldehydes that can be achieved under metal-free conditions through a sequence of oxidative decarbonylation, radical addition across C–C unsaturated bonds, C–H functionalization and annulation (Scheme 1b).^10^ To the best of our knowledge, this method is the first metal-free radical-mediated decarbonylative [2 + 2 + 2] carbocyclization reaction of *N*-(2-ethynylaryl)acrylamides with tertiary alkyl aldehydes as a two carbon unit, which provides selective and straightforward access to important six-membered carbocycle-fused polycyclic skeletons, including tetrahydrophenanthridin-6(5*H*)-ones, 6*H*-indeno[1,2-*c*]quinolin-6-one, 6*H*-benzo[*c*]chromen-6-one and 1*H*-fluorene.^9^ Moreover, this reaction is applicable to secondary alkyl aldehydes and the selectivity is shifted towards the decarbonylative [2 + 2 + 1] carbocyclization with *N*-(2-ethynylaryl)acrylamides.

## Results and discussion

Our initial investigations focused on optimization of the decarbonylative [2 + 2 + 2] carbocyclization between *N*-methyl-*N*-(2-(phenylethynyl)phenyl)methacrylamide (**1a**) and pivalaldehyde (**2a**) (Table 1).^11^ When amide **1a** and aldehyde **2a** were subjected to *tert*-butyl perbenzoate (TBPB) oxidation in PhCF_3_ at 100 °C for 24 h, the desired decarbonylative [2 + 2 + 2] carbocyclization product **3aa** was obtained in 81% yield (entry 1). Inspired by this result, a series of other oxidants, including *tert*-butyl hydroperoxide (TBHP), di-*tert*-butyl peroxide (DTBP) and K_2_S_2_O_8_, were examined (entries 2–4): they showed lower activity for the reaction than TBPB in terms of yields. A screening of the amount of TBPB revealed that 3 equiv. of TBPB was the best choice for further optimization (entries 5 and 6). We found that while a higher reaction temperature (110 °C) slightly affected the reaction (entry 7), a lower reaction temperature (90 °C) had an obviously negative effect (entry 8). Other solvents, including MeCN (entry 9), MeCONMe_2_ (entry 10) and PhCl (entry 11), were found to be less effective than PhCF_3_, thus giving diminished yields. Gratifyingly, the reaction could be satisfactorily performed at a 1 gram scale of amide **1a**, providing **3aa** in moderate yield (entry 12).

**Table 1:** Screening of optimal reaction conditions^*a*^

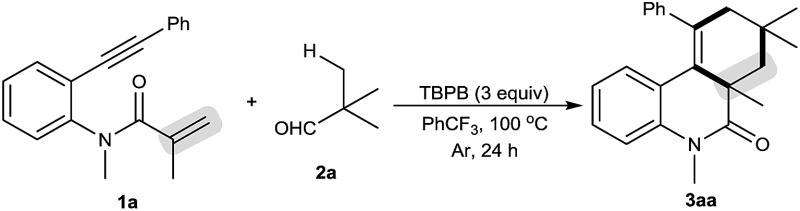		
Entry	Variation from the standard conditions	Isolated yield (%)
1	None	81
2	TBHP instead of TBPB	10
3	DTBP instead of TBPB	22
4	K_2_S_2_O_8_ instead of TBPB	Trace
5	TBPB (3.5 equiv.)	80
6	TBPB (2.5 equiv.)	71
7	At 110 °C	80
8	At 90 °C	62
9	MeCN instead of PhCF_3_	67
10	MeCONMe_2_ instead of PhCF_3_	20
11	PhCl instead of PhCF_3_	40
12^*b*^	None	73

^*a*^Reaction conditions: **1a** (0.2 mmol), **2a** (2 equiv.), TBPB (3 equiv.), PhCF_3_ (2 mL), argon, 100 °C for 24 h. TBHP (5 M in decane). Some by-products, including vinyl C–N bond-decomposition products, were observed.

^*b*^ **1a** (1 g, 3.64 mmol) and PhCF_3_ (5 mL) for 48 h.

The scope of this decarbonylative [2 + 2 + 2] carbocyclization protocol was probed with regard to both the *N*-(2-ethynylaryl)acrylamide **1** and aldehyde **2** (Table 2). Gratifyingly, a variety of *N*-(2-ethynylaryl)acrylamides **1b–s** underwent the reaction with pivalaldehyde (**2a**) and TBPB to afford phenanthridin-6(5*H*)-ones **3** in moderate to good yields. We found that amides **1b–e** bearing a wide range of *N*-substituents, such as *N*-Bn, *N*-allyl, *N*-Ts and even free *N*–H, were viable substrates to assemble **3ba–ea** in moderate yields. With respect to the alkyne moiety in *N*-(2-ethynylaryl)acrylamides, the reaction was perfectly tolerant of various aryl substituents, namely 4-MeC_6_H_4_, 4-MeOC_6_H_4_, 4-BrC_6_H_4_, 4-ClC_6_H_4_, 4-FC_6_H_4_, 4-CNC_6_H_4_, 3-MeC_6_H_4_, 3-BrC_6_H_4_ and pyridin-3-yl groups, at the terminal alkyne, and the nature of the aryl group had no detrimental effect on the reaction (**3fa–na**).^11^ It was noted that in the case of the alkyl-substituted alkyne **1o** a moderate yield of the product (**3oa**) was still achieved. It is important to emphasize that amides **1p–r** with substituents, such as Me and Cl, on the 4- or 5-position of the *N*-aryl moiety were smoothly converted to **3pa–ra** in 70–75% yields. To our surprise, amide **1s** with a phenyl group on the 2-position of the acrylamide moiety shifted the selectivity towards functionalization of the phenyl C(sp^2^)–H bond, not the *tert*-butyl C(sp^3^)–H bond, thus furnishing 6*H*-indeno[1,2-*c*]quinolin-6-one **3sa** rather than the phenanthridin-6(5*H*)-one.

**Table 2:** Carbocyclization of *N*-(2-ethynylaryl)acrylamides (**1**) with tertiary alkyl aldehydes (**2**)^*a*^

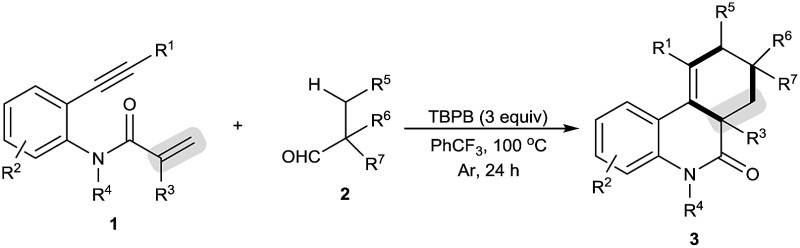
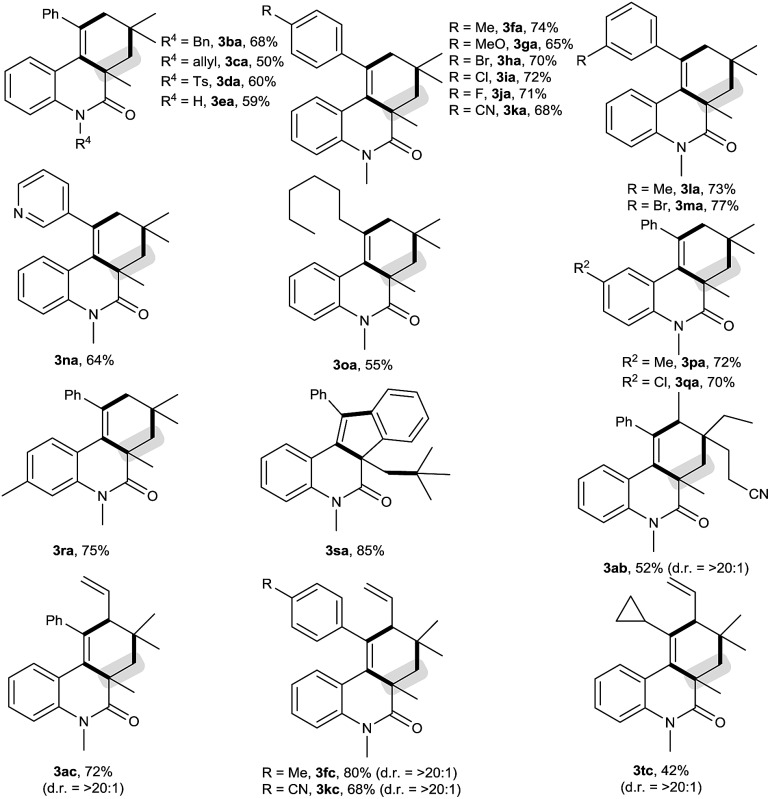

^*a*^Reaction conditions: **1** (0.2 mmol), **2a** (2 equiv.), TBPB (3 equiv.), PhCF_3_ (2 mL), argon, 100 °C for 24 h.

In the presence of amide **1a** and TBPB, tertiary alkyl aldehydes, namely 3-ethyl-3-formylpentanenitrile (**2b**) and 2,2-dimethylpent-4-enal (**2c**), were also found to be viable substrates to produce the decarbonylative [2 + 2 + 2] carbocyclization products **3ab** and **3ac**. Notably, in the case of 2,2-dimethylpent-4-enal (**2c**) the functionalization of the more active allyl C(sp^3^)–H bond takes precedence over the methyl C(sp^3^)–H bond: 2,2-dimethylpent-4-enal (**2c**) successfully underwent the reaction with *N*-(2-ethynylaryl)acrylamides **1f**, **1k** and **1t**, affording **3fc**, **3kc** and **3tc** in moderate to good yields with high diastereoselectivity.

**Scheme 2:**
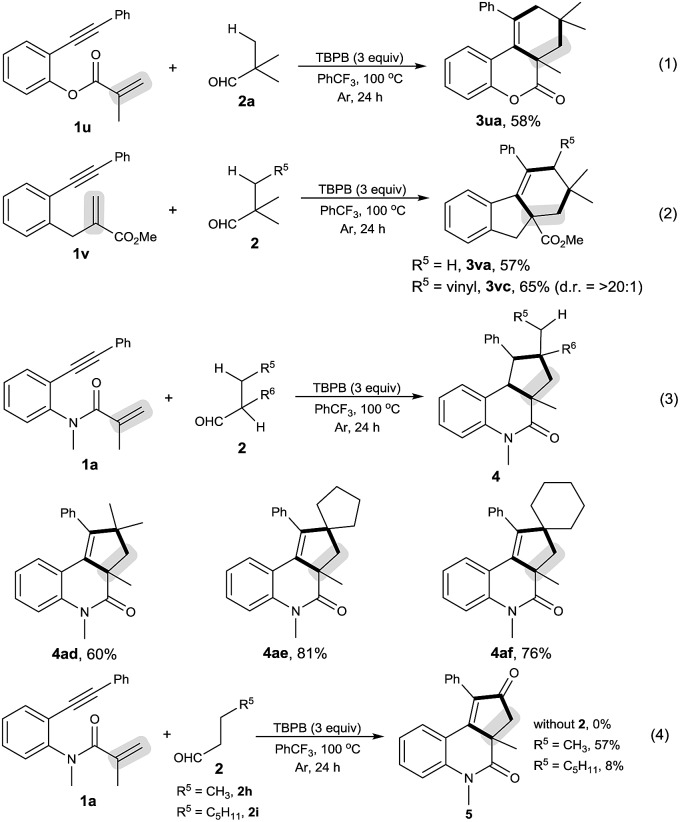
Variation of other 1,*n*-enynes and secondary alkyl aldehydes.

As shown in Scheme 2, the decarbonylative [2 + 2 + 2] carbocyclization protocol also allowed the formation of useful 6*H*-benzo[*c*]chromen-6-one **3ua** (eqn (1)) and 1*H*-fluorenes **3va** and **3vc** (eqn (2)) from the corresponding 2-(phenylethynyl)phenyl methacrylate (**1u**) and methyl 2-(2-(phenylethynyl)benzyl)acrylate (**1v**), but a linear enyne, 4-methyl-*N*-(2-methylallyl)-*N*-(3-phenylprop-2-yn-1-yl)-benzenesulfonamide (**1w**), was not a suitable substrate.

We found that secondary alkyl aldehydes **2d–f** were viable substrates to perform the decarbonylative carbocyclization reaction, but the selectivity was shifted towards the functionalization of the C(sp^3^)–H bond adjacent to the aldehyde group, resulting in the construction of five-membered carbocycle-fused polycyclic skeletons (eqn (3)). For example, isobutyraldehyde (**2d**) underwent the decarbonylative [2 + 2 + 1] carbocyclization reaction to afford 4*H*-cyclopenta[*c*]quinolin-4-one **4ad** in 60% yield. Using cyclopentanecarbaldehyde (**2e**) or cyclohexanecarbaldehyde (**2f**) also delivered the decarbonylative [2 + 2 + 1] carbocyclization products **4ae** and **4af** with a spirocyclic scaffold. Unfortunately, cyclopropane aldehyde (**2g**) was not a suitable aldehyde. Attempts to carbocyclize primary alkyl aldehydes, such as butyraldehyde (**2h**) and caprylic aldehyde (**2i**), failed to give the desired products. but instead provided the CO-inserted Pauson–Khand-type product, 3*a*,5-dimethyl-1-phenyl-3,3*a*-dihydro-2*H*-cyclopenta[*c*]quinoline-2,4(5*H*)-dione (**5**), in 57% and 8% yields, respectively (eqn (4)).^7^

**Scheme 3:**
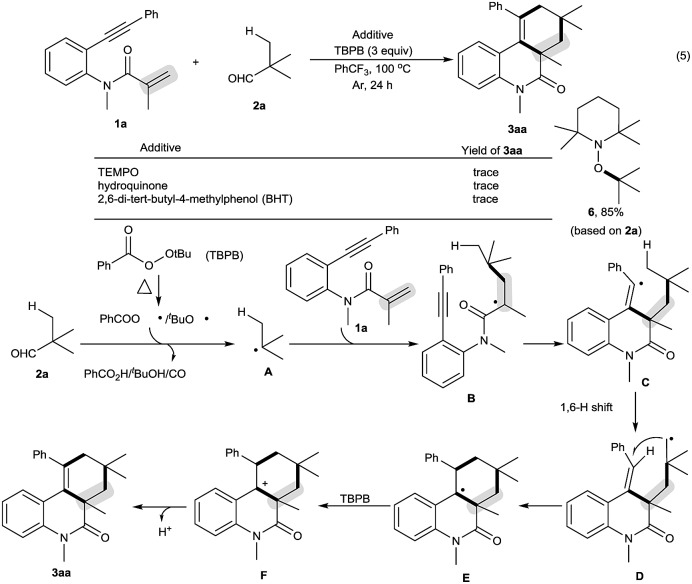
Control experiments and possible reaction mechanism.

To understand the mechanism, the control reaction of amide **1a** with aldehyde **2a** and TBPB was completely suppressed by a stoichiometric amount of radical inhibitors, including TEMPO, hydroquinone and BHT. Moreover, aldehyde **2a** was converted into 1-(*tert*-butoxy)-2,2,6,6-tetramethylpiperidine (**6**) by reacting with TEMPO (eqn (5) in Scheme 3). These results implied that the decarbonylative [2 + 2 + 2] carbocyclization protocol involves a radical process. Therefore, the mechanism of this reaction is presented in Scheme 3.^4,5,8,10^ Aldehyde **2a** is converted into alkyl radical **A** *via* oxidative decarbonylation with the aid of TBPB.^5^ Addition of the alkyl radical **A** across the C–C double bond in amide **1a** affords new alkyl radical intermediate **B**, followed by cyclization to produce unstable vinyl radical intermediate **C**.^4,10^ The intermediate **C** readily undergoes a 1,6-H shift followed by annulation to form intermediate **E**.^8^ Oxidation of intermediate **E** by TBPB affords the cationic intermediate **F**.^4,5,8^ Finally, deprotonation of intermediate **F** results in the formation of the product **3aa**.

## Conclusions

In summary, we have developed the first oxidative decarbonylative [2 + 2 + *m*] annulation reaction of 1,*n*-enynes with tertiary and secondary alkyl aldehydes as a two-carbon unit source for the synthesis of diverse six-membered carbocycle-fused polycycles under metal-free conditions. This method proceeds through a sequence of oxidative decarbonylation, 1,6-H shift and annulation, and allows the one-step formation of three new C–C bonds with broad substrate scope and excellent functional group tolerance. Moreover, this method is applicable to secondary alkyl aldehydes leading to five-membered carbocyclic-ring-fused polycycles. Further studies will focus on the development of enantioselective aspects and other 1,*n*-enyne oxidative radical annulation reactions.
