# Supplementary material for: Intermolecular oxidative decarbonylative [2 + 2 + 2] carbocyclization of N-(2-ethynylaryl)acrylamides with tertiary and secondary alkyl aldehydes involving C(sp3)–H functionalization
Source: Chem Sci. 2016 Jul 21;7(12):7050–4. doi: 10.1039/c6sc02451c (PMC5282743; doi:10.1039/c6sc02451c)

Electronic Supplementary Material (ESI) for Chemical Science.
This journal is © The Royal Society of Chemistry 2016

Supporting Information

Intermolecular Oxidative Decarbonylative [2+2+2] Carbocyclization of *N*-(2-Ethynylaryl)acrylamides with Tertiary and Secondary Alkyl Aldehydes involving C(sp^3)-H Functionalization

Yang Li, Gao-Hui Pan, Ming Hu, Bang Liu, Ren-Jie Song*, and Jin-Heng Li*

State Key Laboratory of Chemo/Biosensing and Chemometrics, College of Chemistry and Chemical Engineering, Hunan University, Changsha 410082, China, Key Laboratory of Jiangxi Province for Persistent Pollutants Control and Resources Recycle, Nanchang Hangkong University, Nanchang 330063, China, and State Key Laboratory of Applied Organic Chemistry Lanzhou University, Lanzhou 730000,China

E-mail: srj0731@hnu.edu.cn and jhli@hnu.edu.cn

List of Contents

(A) Typical experimental procedure

(B) Analytical data

(C) References

(D) Additional experiments

(E) Spectra

(F) The X-ray single-crystal diffraction analysis of 3ha

(A) General Experimental Procedures

(a) Preparation of Substrates 1:

Substrates **1** were prepared according to literature procedures.[1]

(b) General Procedures for the Decarbonylative [2+2+2] Annulation of Benzene-Linked 1,*n*-Enynes with Alkyl Aldehydes Involving C(sp^3)-H Functionalization:

To a Schlenk tube were added benzene-linked 1,*n*-enyne **1** (0.2 mmol), **2** (2 equiv), and TBPB (3 equiv). Then the mixture was stirred in $PhCF_3$ at 100 °C (oil bath temperature) under argon atmosphere (1 atm) for 24 h until complete consumption of startingmaterial as monitored by TLC and/or GC-MS analysis. After the reaction wasfinished, the reaction mixture was washed with brine. The aqueous phase wasre-extracted with EtOAc (3×10 mL). The combined organic extracts were dried over Na_2SO_4 and concentrated in vacuum. The residue was purified by silica gel flash column chromatography to afford the desired products **3**.

(B) Analytical data

5,6a,8,8-Tetramethyl-10-phenyl-6a,7,8,9-tetrahydrophenanthridin-6(5*H*)-one (3aa):

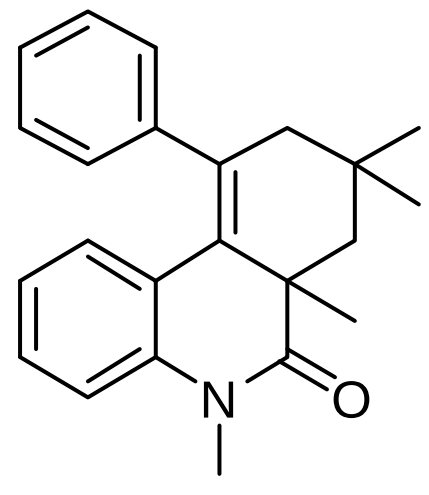

Yellow oil; ^{1}H NMR (400 MHz, $CDCl_3$) δ: 7.21-7.16 (m, 3H), 7.11 (t, *J* = 7.6 Hz, 1H), 6.98-6.93 (m, 3H), 6.65-6.58 (m, 2H), 3.40 (s, 3H), 2.71 (d, *J* = 14.0 Hz, 1H), 2.49 (d, *J* = 16.8 Hz, 1H), 2.01 (d, *J* = 16.8 Hz, 1H), 1.46 (d, *J* = 13.6 Hz, 1H), 1.11 (s, 3H), 1.06 (s, 3H), 0.95 (s, 3H); ^{13}C NMR (100 MHz, $CDCl_3$) δ: 174.3, 143.6, 138.9, 136.7, 130.0, 128.6, 128.5, 128.1, 127.1, 126.4, 125.5, 121.7, 113.7, 47.1, 43.7, 42.9, 30.4, 30.3, 29.5, 26.4,

26.3; IR ν CO (KBr, cm^{-1}): 1680, 1594, 1456; LRMS (EI, 70 eV) m/z (%): 331 (M^+, 90), 314 (100), 256 (34); HRMS *m/z* (ESI) calcd for $C_{23}H_{26}NO$ $[M+H]^+$ 332.2209, found 332.2014.

5-Benzyl-6a,8,8-trimethyl-10-phenyl-6a,7,8,9-tetrahydrophenanthridin-6(5*H*)-one (3ba):

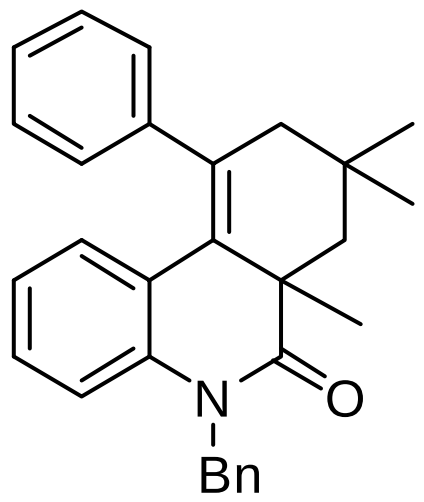

Yellow oil; ^1H NMR (400 MHz, $CDCl_3$) δ: 7.30 (t, *J* = 7.6 Hz, 2H), 7.24-7.15 (m, 6H), 6.97-6.93 (m, 3H), 6.84 (d, *J* = 8.4 Hz, 1H), 6.60-6.55 (m, 2H), 5.31 (d, *J* = 16.0 Hz, 1H), 5.13 (d, *J* = 16.0 Hz, 1H), 2.77 (d, *J* = 14.0 Hz, 1H), 2.53 (d, *J* = 16.8 Hz, 1H), 2.04 (d, *J* = 16.8 Hz, 1H), 1.49 (d, *J* = 14.0 Hz, 1H), 1.20 (s, 3H), 1.13 (s, 3H), 0.99 (s, 3H); ^{13}C NMR (100 MHz, $CDCl_3$) δ: 174.2, 143.6, 138.1, 137.5, 137.0, 130.1, 128.6, 128.5, 128.5, 128.2, 127.1, 126.9, 126.5, 126.3, 125.9, 121.8, 114.8, 47.1, 46.5, 43.7, 43.1, 30.4, 29.6, 26.5, 26.4; IR ν CO (KBr, cm^{-1}): 1690, 1587, 1462; LRMS (EI, 70 eV) m/z (%): 407 (M^+, 84), 274 (11), 91 (100); HRMS *m/z* (ESI) calcd for $C_{29}H_{30}NO$ $[M+H]^+$ 408.2322, found 408.2329.

5-Allyl-6a,8,8-trimethyl-10-phenyl-6a,7,8,9-tetrahydrophenanthridin-6(5*H*)-one (3ca):

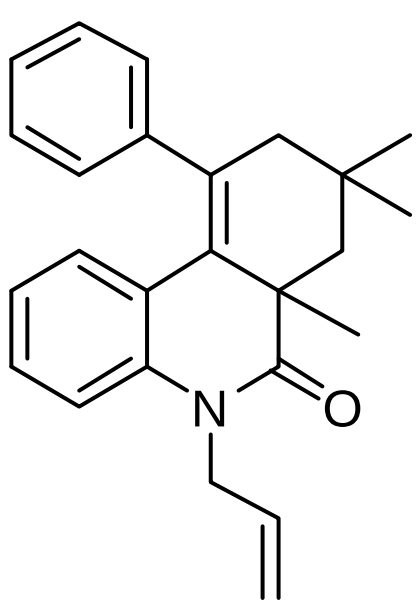

Yellow oil; ^1H NMR (400 MHz, $CDCl_3$) δ: 7.21-7.14 (m, 3H), 7.08-7.04 (m, 1H), 6.98-6.92 (m, 3H), 6.63-6.58 (m, 2H), 5.96-5.87 (m, 1H), 5.17 (t, *J* = 14.4 Hz, 2H), 4.79-4.74 (m, 1H), 4.44-4.38 (m, 1H), 2.73-2.69 (m, 1H), 2.50 (d, *J* = 16.8 Hz, 1H), 2.04-1.99 (m, 1H), 1.43 (d, *J* = 3.6 Hz, 1H), 1.12 (s, 3H), 1.10 (s, 3H), 0.95 (s, 3H); ^{13}C NMR (100 MHz, $CDCl_3$) δ: 173.7, 143.7, 138.2, 136.9, 132.8, 130.1, 128.6, 128.5, 128.2, 127.1, 126.4, 125.6, 121.7, 115.9, 114.4, 47.1, 45.4, 43.6, 43.0, 30.3, 29.5, 26.4, 26.3; IR ν CO (KBr, cm^{-1}): 1677, 1588, 1461; LRMS (EI, 70 eV) m/z (%): 357 (M^+,

92), 342 (100), 238 (11); HRMS *m/z* (ESI) calcd for $C_{25}H_{28}NO$ [M+H]$^+$ 358.2165, found 358.2172.

6a,8,8-Trimethyl-10-phenyl-5-tosyl-6a,7,8,9-tetrahydrophenanthridin-6(5*H*)-one (3da) :

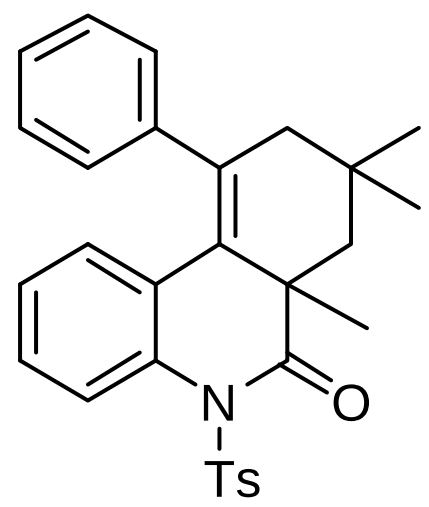

White solid, mp 192.4-194.1°C (uncorrected); ^{1}H NMR (400 MHz, $CDCl_3$) δ: 7.88 (d, *J* = 8.0 Hz, 2H), 7.61 (d, *J* = 8.4 Hz, 1H), 7.24-7.11 (m, 6H), 6.96 (d, *J* = 6.8 Hz, 2H), 6.81 (t, *J* = 7.6 Hz, 1H), 6.52 (d, *J* = 7.6 Hz, 1H), 2.41 (dd, *J* = 14.0, 2.4 Hz, 1H), 2.31 (s, 3H), 1.76 (dd, *J* = 16.4, 2.0 Hz, 1H), 1.50 (s, 3H), 1.21 (d, *J* = 14.0 Hz, 1H), 0.87 (s, 3H), 0.81 (s, 3H), 0.67 (d, *J* = 2.4 Hz, 1H); ^{13}C NMR (100 MHz, $CDCl_3$) δ: 175.8, 143.8, 137.8, 135.67, 130.1, 128.4, 128.3, 128.2, 127.2, 126.5, 123.7, 121.8, 114.5, 47.3, 42.9, 42.7, 30.4, 29.4, 26.7, 25.9; IR ν CO (KBr, cm^{-1}): 1682, 1583, 1472; LRMS (EI, 70 eV) m/z (%): 471 (M$^+$, 21), 315 (100), 274 (33); HRMS *m/z* (ESI) calcd for $C_{29}H_{30}NO_3S$ [M+H]$^+$ 472.1941, found 472.1933.

6a,8,8-Trimethyl-10-phenyl-6a,7,8,9-tetrahydrophenanthridin-6(5*H*)-one (3ea):

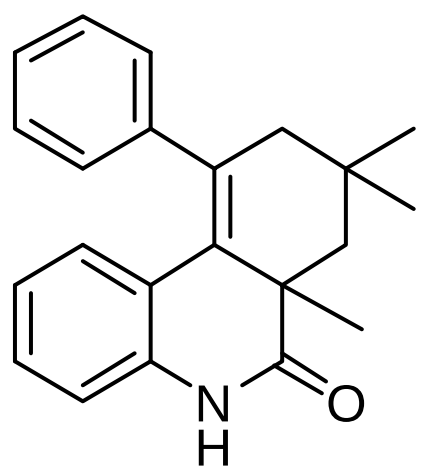

Yellow oil; ^{1}H NMR (400 MHz, $CDCl_3$) δ: 8.04 (s, 1H), 7.23-7.17 (m, 3H), 7.05-6.97 (m, 3H), 6.76 (d, *J* = 8.0 Hz, 1H), 6.61-6.54 (m, 2H), 2.70 (d, *J* = 14.0 Hz, 1H), 2.50 (d, *J* = 17.2 Hz, 1H), 2.00 (d, *J* = 16.8 Hz, 1H), 1.44 (d, *J* = 14.4 Hz, 1H), 1.17 (s, 3H), 1.11 (s, 3H), 0.98 (s, 3H); ^{13}C NMR (100 MHz, $CDCl_3$) δ: 175.8, 143.8, 137.8, 135.7, 130.1, 128.4, 128.3, 128.2, 127.2, 126.5, 123.7, 121.8, 114.5, 47.3, 42.9, 42.7, 30.4, 29.4, 26.7, 25.9; IR ν CO (KBr, cm^{-1}): 1676, 1563, 1452; LRMS (EI, 70 eV) m/z (%): 424 (M$^+$, 27), 317 (99), 302 (100), 246 (17); HRMS *m/z* (ESI) calcd for $C_{22}H_{24}NO$ [M+H]$^+$ 318.1852, found 318.1855.

5,6a,8,8-Tetramethyl-10-(*p*-tolyl)-6a,7,8,9-tetrahydrophenanthridin-6(5*H*)-one (3fa):

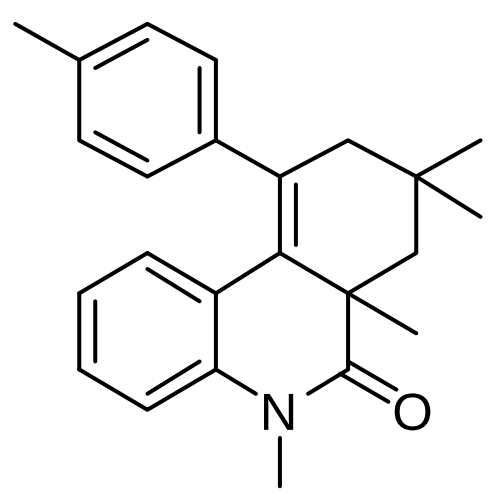

Yellow oil; ^{1}H NMR (400 MHz, $CDCl_3$) δ: 7.21-7.14 (m, 3H), 6.97 (d, *J* = 6.8 Hz, 2H), 6.90 (d, *J* = 8.0 Hz, 1H), 6.82 (d, *J* = 8.4 Hz, 1H), 6.36 (s, 1H), 3.38 (s, 3H), 2.68 (d, *J* = 13.6 Hz, 1H), 2.48 (d, *J* = 15.2 Hz, 1H), 2.01 (d, *J* = 16.8 Hz, 1H), 1.92 (s, 3H), 1.45 (d, *J* = 14.0 Hz, 1H), 1.11 (s, 3H), 1.07 (s, 3H), 0.95 (s, 3H); ^{13}C NMR (100 MHz, $CDCl_3$) δ: 174.2, 143.7, 136.6, 136.5, 130.9, 130.7, 128.8, 128.5, 128.0, 127.6, 126.4, 125.3, 113.4, 47.1, 43.7, 42.9, 30.4, 30.3, 29.5, 26.5, 26.4, 20.4; IR ν CO (KBr, cm^{-1}): 1677, 1595, 1456; LRMS (EI, 70 eV) m/z (%): 345 (M^+, 84), 330 (100), 288 (22); HRMS *m/z* (ESI) calcd for $C_{24}H_{28}NO$ $[M+H]^+$ 346.2165, found 346.2167.

10-(4-Methoxyphenyl)-5,6a,8,8-tetramethyl-6a,7,8,9-tetrahydrophenanthridin-6(5*H*)-one (3ga):

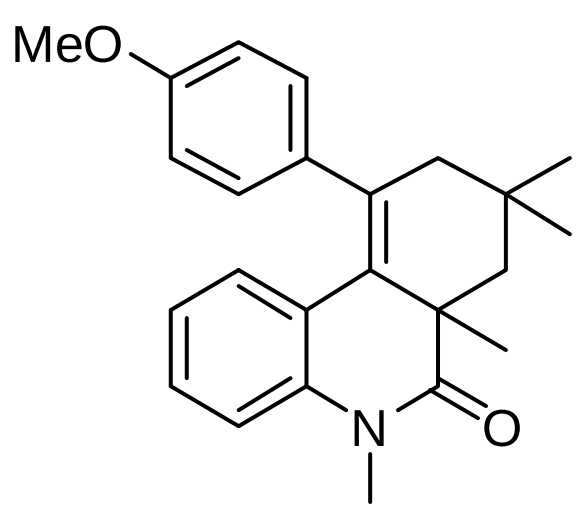

Yellow oil; ^{1}H NMR (400 MHz, $CDCl_3$) δ: 7.21-7.14 (m, 3H), 6.97 (d, *J* = 6.8 Hz, 2H), 6.90 (d, *J* = 8.0 Hz, 1H), 6.82 (d, *J* = 8.4 Hz, 1H), 6.36 (s, 1H), 3.38 (s, 3H), 2.68 (d, *J* = 13.6 Hz, 1H), 2.48 (d, *J* = 15.2 Hz, 1H), 2.01 (d, *J* = 16.8 Hz, 1H), 1.92 (s, 3H), 1.45 (d, *J* = 14.0 Hz, 1H), 1.11 (s, 3H), 1.07 (s, 3H), 0.95 (s, 3H); ^{13}C NMR (100 MHz, $CDCl_3$) δ: 174.2, 143.7, 136.6, 136.5, 130.9, 130.7, 128.8, 128.5, 128.0, 127.6, 126.4, 125.3, 113.4, 47.1, 43.7, 42.9, 30.4, 30.3, 29.5, 26.5, 26.4, 20.4; IR ν CO (KBr, cm^{-1}): 1682, 1593, 1457; LRMS (EI, 70 eV) m/z (%): 361 (M^+, 91), 346 (100), 253 (66); HRMS *m/z* (ESI) calcd for $C_{24}H_{28}NO_2$ $[M+H]^+$ 362.2115, found 362.2119.

10-(4-Bromophenyl)-5,6a,8,8-tetramethyl-6a,7,8,9-tetrahydrophenanthridin-6(5*H*)-one (3ha):

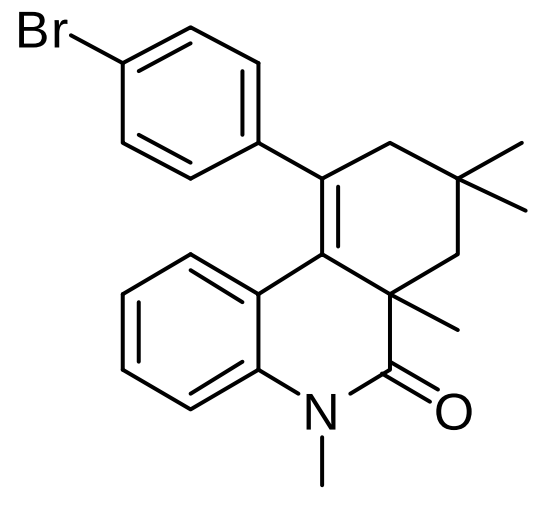

White solid, mp 175.2-176.1°C (uncorrected); ^{1}H NMR (400 MHz, $CDCl_3$) δ: 7.32 (d, *J* = 8.0 Hz, 2H), 7.15-7.11 (m, 1H), 6.95 (d, *J* = 8.0 Hz, 1H), 6.85 (d, *J* = 8.0 Hz, 2H), 6.69 (d, *J* = 7.6 Hz, 1H), 6.59 (d, *J* = 6.8 Hz, 1H), 3.40 (s, 3H), 2.69 (dd, *J* = 14.0, 1.6 Hz, 1H), 2.47 (d, *J* = 15.2 Hz, 1H), 1.96 (dd, *J* = 16.8, 2.0 Hz, 1H), 1.44 (d, *J* = 14.0 Hz, 1H), 1.10 (s, 3H), 1.05 (s, 3H), 0.93 (s, 3H); ^{13}C NMR (100 MHz, $CDCl_3$) δ: 174.0, 142.5, 139.0, 135.2, 131.3, 130.3, 129.9, 129.5, 127.4, 125.1, 121.9, 120.3, 113.8, 46.9, 43.6, 43.0, 30.4, 30.3, 29.5, 26.4, 26.2; IR ν CO (KBr, cm^{-1}): 1680, 1593, 1455; LRMS (EI, 70 eV) m/z (%): 411 (M^{+}+2, 86), 409 (M^{+}, 86), 396 (100), 238 (19); HRMS *m/z* (ESI) calcd for $C_{23}H_{25}BrNO$ $[M+H]^+$ 410.1114, found 410.1117.

10-(4-Chlorophenyl)-5,6a,8,8-tetramethyl-6a,7,8,9-tetrahydrophenanthridin-6(5*H*)-one (3ia):

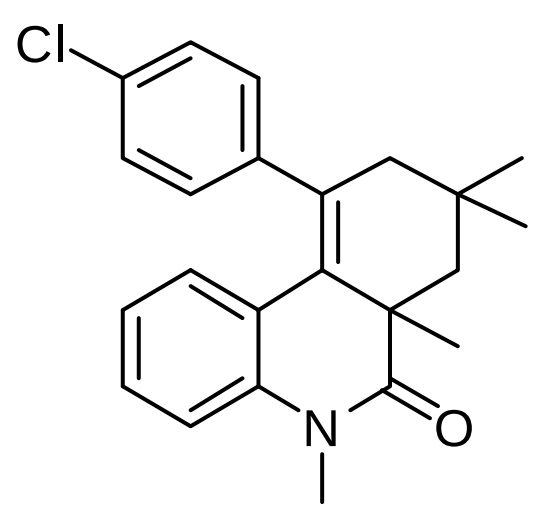

Yellow oil; ^{1}H NMR (400 MHz, $CDCl_3$) δ: 7.18-7.12 (m, 3H), 6.96-6.90 (m, 3H), 6.68 (t, *J* = 7.6 Hz, 1H), 6.59 (d, *J* = 7.2 Hz, 1H), 3.40 (s, 3H), 2.69 (dd, *J* = 14.0, 1.6 Hz, 1H), 2.46 (d, *J* = 16.8 Hz, 1H), 1.96 (dd, *J* = 16.8, 1.6 Hz, 1H), 1.44 (d, *J* = 14.0 Hz, 1H), 1.10 (s, 3H), 1.06 (s, 3H), 0.94 (s, 3H); ^{13}C NMR (100 MHz, $CDCl_3$) δ: 174.1, 142.0, 139.0, 135.2, 132.2, 129.9, 129.9, 129.5, 128.4, 127.4, 125.1, 121.9, 113.8, 46.9, 43.6, 43.0, 30.4, 30.3, 29.5, 26.4, 26.2; IR ν CO (KBr, cm^{-1}): 1679, 1594, 1457; LRMS (EI, 70 eV) m/z (%): 367 (M^{+}+2, 29), 365 (M^{+}, 87), 350 (100), 254 (34); HRMS *m/z* (ESI) calcd for $C_{23}H_{25}ClNO$ $[M+H]^+$ 366.1619, found 366.1615.

10-(4-Fluorophenyl)-5,6a,8,8-tetramethyl-6a,7,8,9-tetrahydrophenanthridin-6(5*H*)-one (3ja):

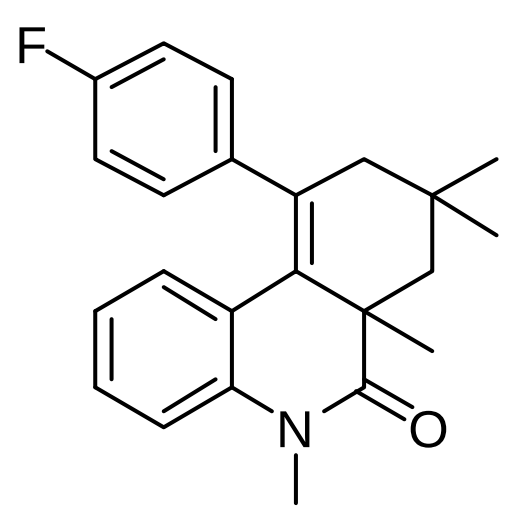

Yellow oil; ^{1}H NMR (400 MHz, $CDCl_3$) δ: 7.13 (t, *J* = 7.6 Hz, 1H), 6.96-6.87 (m, 5H), 6.67 (t, *J* = 7.6 Hz, 1H), 6.57 (d, *J* = 8.0

Hz, 1H), 3.40 (s, 3H), 2.69 (d, *J* = 14.0 Hz, 1H), 2.46 (d, *J* = 16.8 Hz, 1H), 1.97 (d, *J* = 16.8 Hz, 1H), 1.45 (d, *J* = 13.6 Hz, 1H), 1.11 (s, 3H), 1.06 (s, 3H), 0.94 (s, 3H); ^{13}C NMR (100 MHz, $CDCl_3$) δ: 174.2, 162.5 (*J* = 244.1 Hz), 139.5, 139.1, 135.5, 130.1 (*J* = 7.7 Hz), 129.9, 129.2, 127.3, 125.3, 121.8, 115.1 (*J* = 21.0 Hz), 113.8, 47.2, 43.7, 43.0, 30.4, 30.3, 29.5, 26.4, 26.3; ^{19}F NMR (375 MHz, $CDCl_3$) δ: -115.81 (m); IR ν CO (KBr, cm^{-1}): 1681, 1597, 1453; LRMS (EI, 70 eV) m/z (%): 349 (M^+, 83), 334 (100), 292 (30); HRMS *m/z* (ESI) calcd for $C_{23}H_{25}FNO$ $[M+H]^+$ 350.1915, found 350.1919.

4-(5,6a,8,8-Tetramethyl-6-oxo-5,6,6a,7,8,9-hexahydrophenanthridin-10-yl)benzonitrile (3ka):

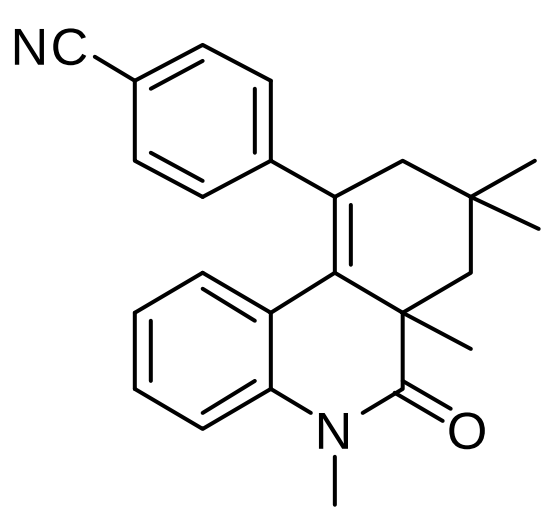

Yellow oil; ^{1}H NMR (400 MHz, $CDCl_3$) δ: 7.49 (d, *J* = 8.0 Hz, 2H), 7.17 (t, *J* = 8.0 Hz, 1H), 7.09 (d, *J* = 7.6 Hz, 2H), 6.98 (d, *J* = 8.0 Hz, 1H), 6.67 (d, *J* = 7.6 Hz, 1H), 6.50 (d, *J* = 8.0 Hz, 1H), 3.41 (s, 3H), 2.72 (d, *J* = 14.0 Hz, 1H), 2.49 (d, *J* = 16.4 Hz, 1H), 1.95 (d, *J* = 16.8 Hz, 1H), 1.45 (d, *J* = 14.0 Hz, 1H), 1.12 (s, 3H), 1.07 (s, 3H), 0.94 (s, 3H); ^{13}C NMR (100 MHz, $CDCl_3$) δ: 173.7, 148.7, 139.0, 134.6, 132.0, 131.1, 129.8, 129.4, 127.9, 124.5, 121.9, 118.9, 114.0, 110.1, 46.4, 43.5, 43.1, 30.4, 30.3, 29.6, 26.4, 26.0; IR ν CO (KBr, cm^{-1}): 1679, 1597, 1454; LRMS (EI, 70 eV) m/z (%): 356 (M^+, 54), 321 (100), 217 (19); HRMS *m/z* (ESI) calcd for $C_{24}H_{25}N_2O$ $[M+H]^+$ 357.1961, found 357.1966.

5,6a,8,8-Tetramethyl-10-(*m*-tolyl)-6a,7,8,9-tetrahydrophenanthridin-6(5*H*)-one (3la):

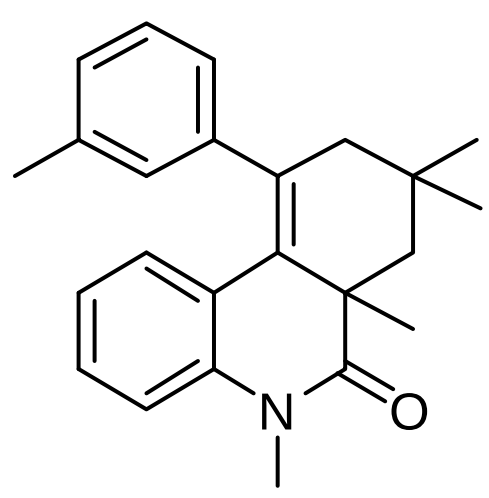

Yellow oil; ^{1}H NMR (400 MHz, $CDCl_3$) δ: 7.12-7.04 (m, 2H), 6.98-6.93 (m, 2H), 6.82 (s, 1H), 6.72 (d, *J* = 7.2 Hz, 1H), 6.66-6.61 (m, 2H), 3.40 (s, 3H), 2.69 (d, *J* = 14.0 Hz, 1H), 2.47

(d, J = 16.8 Hz, 1H), 2.25 (s, 3H), 1.99 (d, J = 16.8 Hz, 1H), 1.44 (d, J = 14.0 Hz, 1H), 1.10 (s, 3H), 1.06 (s, 3H), 0.95 (s, 3H); ^{13}C NMR (100 MHz, $CDCl_3$) δ: 174.3, 143.5, 138.9, 137.7, 136.7, 130.0, 129.0, 128.4, 128.0, 127.1, 127.1, 125.6 (2C), 121.7, 113.6, 47.2, 43.7, 42.9, 30.4, 30.3, 29.5, 26.5, 26.2, 21.4; IR ν CO (KBr, cm^{-1}): 1682, 1591, 1456; LRMS (EI, 70 eV) m/z (%): 345 (M^+, 91), 330 (100), 254 (34); HRMS *m/z* (ESI) calcd for $C_{24}H_{28}NO$ $[M+H]^+$ 346.2165, found 346.2166.

10-(3-Bromophenyl)-5,6a,8,8-tetramethyl-6a,7,8,9-tetrahydrophenanthridin-6(5*H*)-one (3ma):

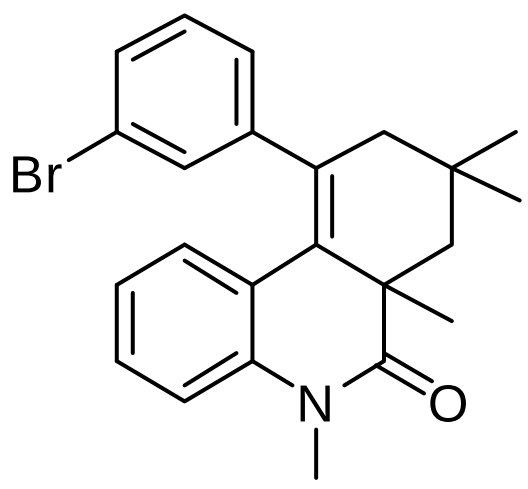

White solid, mp 175.4-176.2°C (uncorrected); ^{1}H NMR (400 MHz, $CDCl_3$) δ: 7.29 (d, J = 8.0 Hz, 1H), 7.18-7.12 (m, 2H), 7.04 (t, J = 8.0 Hz, 1H), 6.95 (d, J = 8.4 Hz, 1H), 6.85 (d, J = 7.2 Hz, 1H), 6.68 (t, J = 7.6 Hz, 1H), 6.60 (d, J = 6.8 Hz, 1H), 3.40 (s, 3H), 2.70 (dd, J = 14.0, 2.0 Hz, 1H), 2.46 (d, J = 16.8 Hz, 1H), 1.97 (dd, J = 16.8, 1.6 Hz, 1H), 1.43 (d, J = 14.0 Hz, 1H), 1.10 (s, 3H), 1.06 (s, 3H), 0.94 (s, 3H); ^{13}C NMR (100 MHz, $CDCl_3$) δ: 174.0, 145.7, 138.9, 134.9, 131.3, 129.9, 129.8, 129.7, 129.5, 127.5, 127.3, 124.8, 122.1, 121.8, 113.8, 46.9, 43.6, 43.0, 30.4, 30.3, 29.5, 26.5, 26.1; IR ν CO (KBr, cm^{-1}): 1679, 1594, 1456; LRMS (EI, 70 eV) m/z (%): 411 (M^++2, 90), 409 (M^+, 90), 394 (100), 238 (10); HRMS *m/z* (ESI) calcd for $C_{23}H_{25}BrNO$ $[M+H]^+$ 410.1114, found 410.1117.

5,6a,8,8-Tetramethyl-10-(pyridin-3-yl)-6a,7,8,9-tetrahydrophenanthridin-6(5*H*)-one (3na):

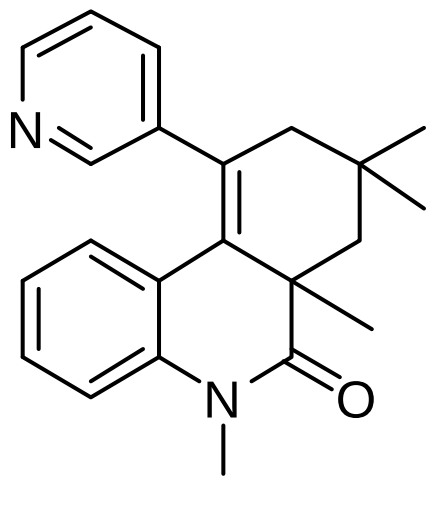

Yellow oil; ^{1}H NMR (400 MHz, $CDCl_3$) δ: 8.41 (d, J = 4.0 Hz, 1H), 8.23 (s, 1H), 7.30 (t, J = 7.2 Hz, 1H), 7.14 (t, J = 6.8 Hz, 2H), 6.97 (d, J = 8.0 Hz, 1H), 6.67 (t, J = 7.6 Hz, 1H), 6.54 (d, J = 7.2 Hz, 1H), 3.41 (s, 3H), 2.72 (dd, J = 14.0, 1.6 Hz, 1H), 2.51 (d, J =

16.8 Hz, 1H), 1.99 (dd, J = 16.8, 1.6 Hz, 1H), 1.47 (d, J = 14.0 Hz, 1H), 1.13 (s, 3H), 1.08 (s, 3H), 0.96 (s, 3H); ^{13}C NMR (100 MHz, $CDCl_3$) δ: 173.8, 149.5, 147.4, 139.2, 139.1, 136.0, 132.7, 131.2, 129.8, 127.7, 124.6, 123.0, 122.0, 114.0, 46.6, 43.5, 43.1, 30.4, 30.2, 29.5, 26.3, 26.1; IR ν CO (KBr, cm^{-1}): 1684, 1595, 1456; LRMS (EI, 70 eV) m/z (%): 332 (M^+, 85), 317 (100), 254 (28); HRMS *m/z* (ESI) calcd for $C_{22}H_{25}N_2O$ $[M+H]^+$ 333.1961, found 333.1964.

10-Hexyl-5,6a,8,8-tetramethyl-6a,7,8,9-tetrahydrophenanthridin-6(5*H*)-one (3oa):

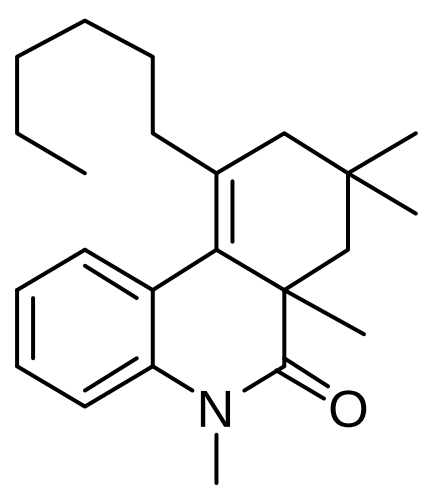

Yellow oil; ^{1}H NMR (400 MHz, $CDCl_3$) δ: 7.26 (t, J = 8.0 Hz, 2H), 7.06 (t, J = 7.6 Hz, 1H), 6.98 (d, J = 8.0 Hz, 1H), 3.30 (s, 3H), 2.60 (dd, J = 14.8, 1.6 Hz, 1H), 2.45-2.38 (m, 1H), 2.10-1.98 (m, 2H), 1.75 (d, J = 16.0 Hz, 1H), 1.56-1.48 (m, 1H), 1.43-1.40 (m, 1H), 1.33-1.25 (m, 7H), 1.03 (s, 3H), 0.94 (s, 3H), 0.87 (t, J = 6.8 Hz, 6H); ^{13}C NMR (100 MHz, $CDCl_3$) δ: 175.0, 139.0, 136.6, 128.4, 127.1, 126.3, 126.1, 122.0, 113.9, 44.5, 44.0, 42.8, 34.7, 31.6, 30.6, 30.3, 29.3, 29.1, 28.5, 26.4, 26.1, 22.6, 14.0; IR ν CO (KBr, cm^{-1}): 1681, 1597, 1452; LRMS (EI, 70 eV) m/z (%): 339 (M^+, 43), 254 (100), 198 (11); HRMS *m/z* (ESI) calcd for $C_{23}H_{34}NO$ $[M+H]^+$ 340.2635, found 340.2641.

2,5,6a,8,8-Pentamethyl-10-phenyl-6a,7,8,9-tetrahydrophenanthridin-6(5*H*)-one (3pa):

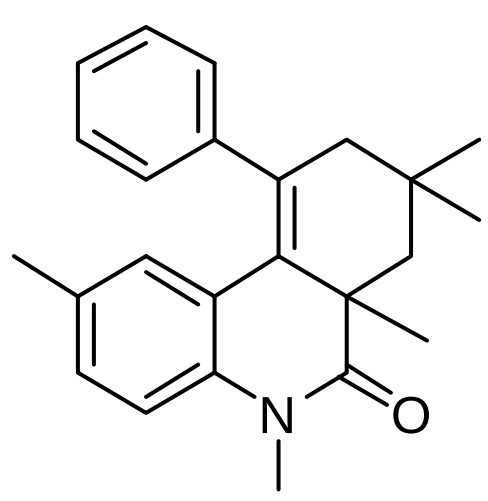

Yellow oil; ^{1}H NMR (400 MHz, $CDCl_3$) δ: 7.21-7.12 (m, 3H), 6.97 (d, J = 6.0 Hz, 2H), 6.90 (d, J = 8.0 Hz, 1H), 6.82 (d, J = 8.4 Hz, 1H), 6.34 (s, 1H), 3.38 (s, 3H), 2.69 (d, J = 13.6 Hz, 1H), 2.48 (d, J = 16.8 Hz, 1H), 2.01 (d, J = 16.8 Hz, 1H), 1.92 (s, 3H), 1.45 (d, J = 14.0 Hz, 1H), 1.11 (s, 3H), 1.07 (s, 3H), 0.95 (s, 3H); ^{13}C NMR (100 MHz, $CDCl_3$) δ: 174.2, 143.7, 136.6, 136.5, 130.9, 130.7, 128.8, 128.5, 128.0, 127.6, 126.4,

125.3, 113.4, 47.1, 43.7, 42.9, 30.4, 30.3, 29.5, 26.5, 26.4, 20.4; IR ν CO (KBr, cm^{-1}): 1680, 1594, 1455; LRMS (EI, 70 eV) m/z (%): 345 (M^+, 90), 330 (100), 288 (24); HRMS *m/z* (ESI) calcd for $C_{24}H_{28}NO$ $[M+H]^+$ 346.2165, found 346.2167.

2-Chloro-5,6a,8,8-tetramethyl-10-phenyl-6a,7,8,9-tetrahydrophenanthridin-6(5*H*)-one (3qa):

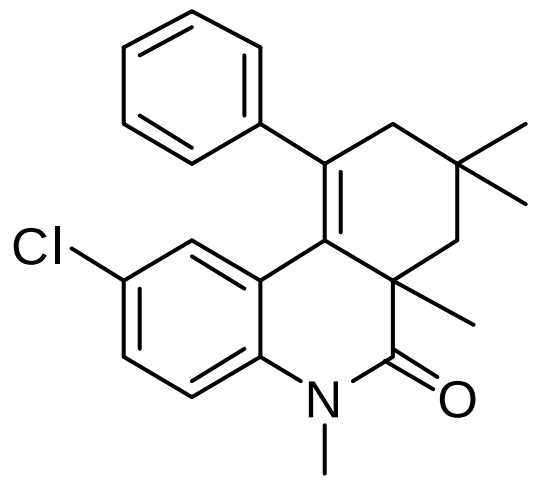

Yellow oil; ^{1}H NMR (400 MHz, $CDCl_3$) δ: 7.24-7.20 (m, 3H), 7.08-7.05 (m, 1H), 6.96 (d, *J* = 6.0 Hz, 2H), 6.86 (d, *J* = 8.8 Hz, 1H), 6.51 (d, *J* = 2.0 Hz, 1H), 3.38 (s, 3H), 2.68 (dd, *J* = 14.0, 2.0 Hz, 1H), 2.49 (d, *J* = 17.2 Hz, 1H), 2.03 (dd, *J* = 17.2, 2.0 Hz, 1H), 1.46 (d, *J* = 13.6 Hz, 1H), 1.11 (s, 3H), 1.07 (s, 3H), 0.94 (s, 3H); ^{13}C NMR (100 MHz, $CDCl_3$) δ: 174.0, 142.8, 138.3, 137.6, 129.7, 128.3, 128.2, 127.5, 127.0, 127.0, 126.9, 126.9, 114.8, 47.1, 43.6, 42.8, 30.5, 30.2, 29.5, 26.4, 26.3; IR ν CO (KBr, cm^{-1}): 1681, 1596, 1455; LRMS (EI, 70 eV) m/z (%): 367 (M^++2, 29), 365 (M^+, 86), 350 (100), 288 (25); HRMS *m/z* (ESI) calcd for $C_{23}H_{25}ClNO$ $[M+H]^+$ 366.1619, found 366.1621.

3,5,6a,8,8-Pentamethyl-10-phenyl-6a,7,8,9-tetrahydrophenanthridin-6(5*H*)-one (3ra):

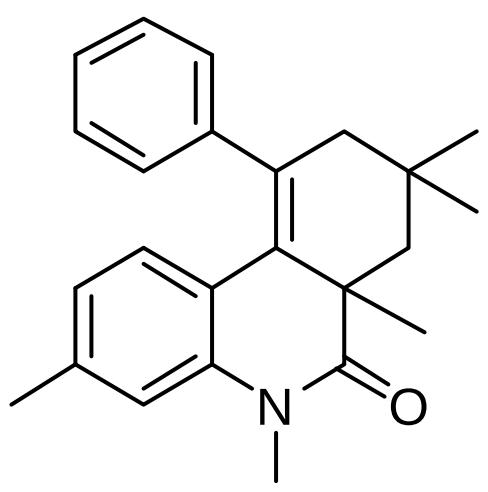

Yellow oil; ^{1}H NMR (400 MHz, $CDCl_3$) δ: 7.22-7.14 (m, 3H), 6.98 (d, *J* = 7.2 Hz, 2H), 6.75 (s, 1H), 6.46 (s, 2H), 3.39 (s, 3H), 2.69 (d, *J* = 13.6 Hz, 1H), 2.47 (d, *J* = 16.8 Hz, 1H), 2.28 (s, 3H), 1.99 (d, *J* = 16.8 Hz, 1H), 1.45 (d, *J* = 14.0 Hz, 1H), 1.11 (s, 3H), 1.06 (s, 3H), 0.95 (s, 3H); ^{13}C NMR (100 MHz, $CDCl_3$) δ: 174.4, 143.8, 138.9, 136.9, 135.9, 129.7, 128.5 (2C), 128.1, 126.3, 122.6, 122.5, 114.5, 47.2, 43.7, 43.0, 30.4, 30.2, 29.5, 26.5, 26.3, 21.5; IR ν CO (KBr, cm^{-1}): 1680, 1594, 1457; LRMS (EI, 70

eV) m/z (%): 345 (M^+, 88), 330 (100), 217 (16); HRMS *m/z* (ESI) calcd for $C_{24}H_{28}NO$ $[M+H]^+$ 346.2165, found 346.2167.

5-Methyl-6a-neopentyl-11-phenyl-5,6a-dihydro-6*H*-indeno[1,2-*c*]quinolin-6-one (3sa):

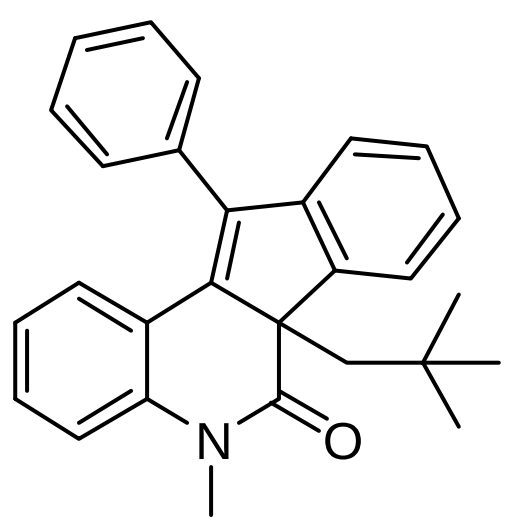

Yellow oil; ^{1}H NMR (400 MHz, $CDCl_3$) δ: 8.01-7.96 (m, 1H), 7.45-7.25 (m, 8H), 7.20-7.18 (m, 1H), 7.13-7.09 (m, 2H), 6.90 (t, *J* = 7.6 Hz, 1H), 3.42 (s, 3H), 2.31 (d, *J* = 14.0 Hz, 1H), 1.86 (d, *J* = 14.0 Hz, 1H), 0.53 (s, 9H); ^{13}C NMR (100 MHz, $CDCl_3$) δ: 170.5, 145.0, 143.9, 139.9, 139.6, 137.5, 134.3, 128.9, 128.7, 128.2, 127.8, 127.6, 127.5, 127.1, 125.3, 122.6, 122.4, 120.5, 114.9, 61.2, 49.4, 33.3, 31.1, 30.6; IR ν CO (KBr, cm^{-1}): 1684, 1591, 1455; LRMS (EI, 70 eV) m/z (%): 393 (M^+, 78), 354 (100), 256 (23); HRMS *m/z* (ESI) calcd for $C_{28}H_{28}NO$ $[M+H]^+$ 394.2165, found 394.2171.

3-(8-Ethyl-5,6a,9-trimethyl-6-oxo-10-phenyl-5,6,6a,7,8,9-hexahydrophenanthridin-8-yl)propanenitrile (3ab):

d.r. > 20:1;

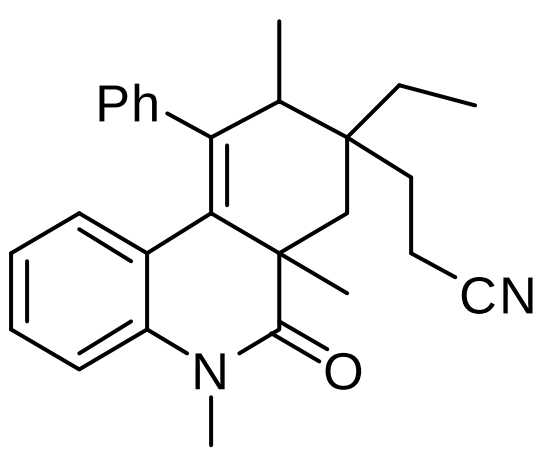

White solid, mp 181.2-182.4°C (uncorrected); ^{1}H NMR (400 MHz, $CDCl_3$) δ: 7.25-7.19 (m, 3H), 7.09 (t, *J* = 6.8 Hz, 3H), 6.91 (d, *J* = 8.0 Hz, 1H), 6.75 (d, *J* = 7.2 Hz, 1H), 6.63 (t, *J* = 7.6 Hz, 1H), 3.41 (s, 3H), 2.84 (d, *J* = 14.8 Hz, 1H), 2.78-2.70 (m, 1H), 2.18-2.09 (m, 1H), 2.00-1.89 (m, 2H), 1.60-1.52 (m, 1H), 1.44-1.35 (m, 2H), 1.26 (d, *J* = 6.4 Hz, 4H), 1.08 (s, 3H), 0.77 (t, *J* = 7.6 Hz, 3H); ^{13}C NMR (100 MHz, $CDCl_3$) δ: 173.9, 143.4, 142.0, 138.4, 130.0, 129.6, 128.8, 128.2, 127.5, 126.6, 125.1, 122.1, 121.0, 113.8, 44.3, 42.7, 36.8, 34.9, 30.9, 29.5, 27.1, 26.7, 15.5, 12.1, 6.4; IR ν CO (KBr, cm^{-1}): 1681, 1582, 1456; LRMS (EI, 70 eV) m/z (%): 384 (M^+, 81), 352

(100), 291 (19); HRMS *m/z* (ESI) calcd for $C_{28}H_{24}O_4$ $[M+H]^+$ 385.2274, found 385.2281.

8,8-Diethyl-5,6a-dimethyl-10-phenyl-9-vinyl-6a,7,8,9-tetrahydrophenanthridin-6(5*H*)-one (3ac):

d.r. > 20:1;

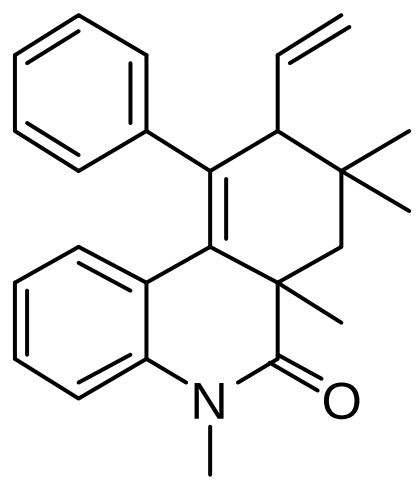

Yellow oil; ^{1}H NMR (400 MHz, $CDCl_3$) δ: 7.13-7.07 (m, 6H), 6.92 (d, *J* = 8.0 Hz, 1H), 6.69-6.66 (m, 1H), 6.61 (t, *J* = 7.6 Hz, 1H), 6.03-5.94 (m, 1H), 5.32 (dd, *J* = 10.0, 2.0 Hz, 1H), 5.17 (d, *J* = 16.8 Hz, 1H), 3.41 (s, 3H), 2.80 (dd, *J* = 14.4, 1.6 Hz, 1H), 2.50 (d, *J* = 8.0 Hz, 1H), 1.51 (d, *J* = 14.4 Hz, 1H), 1.06 (s, 3H), 1.00 (s, 3H), 0.97 (s, 3H); ^{13}C NMR (100 MHz, $CDCl_3$) δ: 173.8, 143.7, 138.9, 138.8, 138.5, 130.4, 130.3, 128.8, 127.8, 127.3, 126.3, 125.6, 121.8, 118.0, 113.5, 58.3, 42.9, 38.8, 32.5, 30.6, 28.2, 26.9, 26.4; IR ν CO (KBr, cm^{-1}): 1683, 1589, 1453; LRMS (EI, 70 eV) m/z (%): 357 (M^+, 100), 314 (16), 260 (24); HRMS *m/z* (ESI) calcd for $C_{27}H_{32}NO$ $[M+H]^+$ 386.2478, found 386.2474.

8,8-Diethyl-5,6a-dimethyl-10-(*p*-tolyl)-9-vinyl-6a,7,8,9-tetrahydrophenanthridin-6(5*H*)-one (3fc):

d.r. > 20:1;

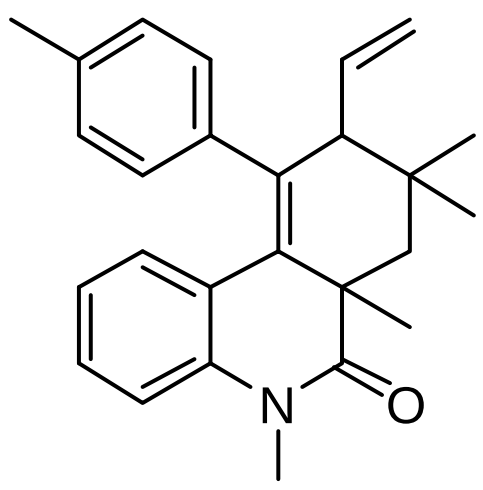

Yellow solid, mp 176.5-177.4°C (uncorrected); ^{1}H NMR (400 MHz, $CDCl_3$) δ: 7.10 (t, *J* = 7.6 Hz, 1H), 7.03-6.89 (m, 5H), 6.71 (d, *J* = 7.6 Hz, 1H), 6.64 (t, *J* = 7.6 Hz, 1H), 6.04-5.92 (m, 1H), 5.32 (d, *J* = 10.0 Hz, 1H), 5.17 (d, *J* = 17.2 Hz, 1H), 3.41 (s, 3H), 2.78 (d, *J* = 14.4 Hz, 1H), 2.48 (d, *J* = 8.4 Hz, 1H), 2.27 (s, 3H), 1.50 (d, *J* = 14.2 Hz, 1H), 1.05 (s, 3H), 0.97 (d, *J* = 5.6 Hz, 6H); ^{13}C NMR (100 MHz, $CDCl_3$) δ: 173.9, 140.9, 139.1, 138.9, 138.4, 135.9, 130.4, 130.0, 128.7, 128.6, 127.2, 125.8, 121.9,

117.9, 113.5, 58.5, 42.9, 38.9, 32.5, 30.6, 28.2, 26.9, 26.4, 21.1; IR ν CO (KBr, cm^{-1}): 1684, 1592, 1458; LRMS (EI, 70 eV) m/z (%): 371 (M^+, 100), 314 (27), 274 (16); HRMS *m/z* (ESI) calcd for $C_{28}H_{34}NO$ $[M+H]^+$ 400.2635, found 400.2639.

4-(8,8-Diethyl-5,6a-dimethyl-6-oxo-9-vinyl-5,6,6a,7,8,9-hexahydrophenanthridin-10-yl)benzonitrile (3kc):

d.r. > 20:1;

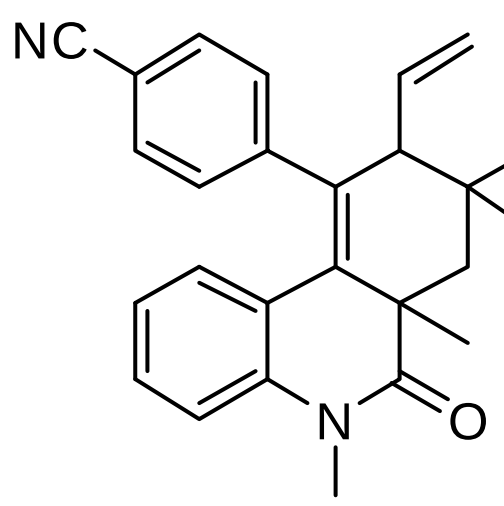

Yellow oil; ^{1}H NMR (400 MHz, $CDCl_3$) δ: 7.44 (d, J = 7.6 Hz, 2H), 7.16 (t, *J* = 8.0 Hz, 3H), 6.96 (d, *J* = 8.0 Hz, 1H), 6.66 (t, *J* = 7.6 Hz, 1H), 6.56 (d, *J* = 7.6 Hz, 1H), 6.09-5.90 (m, 1H), 5.36 (d, *J* = 10.0 Hz, 1H), 5.17 (d, J = 17.2 Hz, 1H), 3.41 (s, 3H), 2.81 (d, *J* = 14.4 Hz, 1H), 2.44 (d, *J* = 8.4 Hz, 1H), 1.51 (d, *J* = 14.4 Hz, 1H), 1.07 (s, 3H), 0.98 (d, *J* = 4.4 Hz, 6H); ^{13}C NMR (100 MHz, $CDCl_3$) δ: 173.3, 148.7, 138.9, 138.7, 136.6, 132.7, 131.8, 130.1, 129.7, 128.1, 124.6, 122.1, 118.9, 118.5, 113.9, 110.1, 57.8, 43.1, 38.7, 32.6, 30.6, 28.1, 26.9, 26.4; IR ν CO (KBr, cm^{-1}): 1680, 1592, 1456; LRMS (EI, 70 eV) m/z (%): 382 (M^+, 91), 367 (100), 285 (11); HRMS *m/z* (ESI) calcd for $C_{28}H_{31}N_2O$ $[M+H]^+$ 411.2431, found 411.2437.

10-Cyclopropyl-8,8-diethyl-5,6a-dimethyl-9-vinyl-6a,7,8,9-tetrahydrophenanthridin-6(5*H*)-one (3tc):

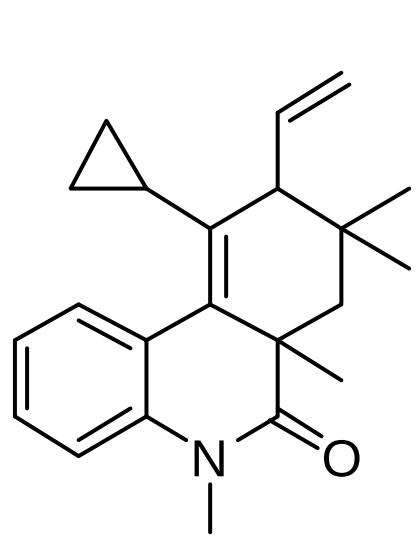

Yellow oil; ^{1}H NMR (400 MHz, $CDCl_3$) δ: 7.68 (d, *J* = 7.6 Hz, 1H), 7.26 (d, *J* = 14.8 Hz, 1H), 7.07 (t, *J* = 7.6 Hz, 1H), 7.01 (d, *J* = 8.0 Hz, 1H), 5.76-5.67 (m, 1H), 5.10 (d, *J* = 10.4 Hz, 1H), 4.84 (d, *J* = 17.2 Hz, 1H), 3.36 (s, 3H), 2.31 (d, *J* = 14.4 Hz, 1H), 1.92 (d, *J* = 7.2 Hz, 1H), 1.87-1.80 (m, 1H), 1.40 (d, *J* = 14.4 Hz, 1H), 1.07 (s, 3H), 1.03 (s, 3H), 0.98 (s, 3H), 0.79-0.65 (m, 2H), 0.60-0.53 (m, 1H), 0.50-0.44 (m, 1H); ^{13}C NMR (100 MHz, $CDCl_3$) δ: 175.7, 139.6, 139.5, 138.4, 136.7, 129.4, 127.2, 126.4, 121.8, 117.5,

114.0, 48.6, 42.6, 39.0, 32.8, 30.2, 29.5, 28.4, 25.5, 14.1, 7.2, 6.8; IR ν CO (KBr, cm^{-1}): 1677, 1591, 1452; LRMS (EI, 70 eV) m/z (%): 321 (M^+, 91), 306 (100), 250 (54); HRMS *m/z* (ESI) calcd for $C_{24}H_{32}NO$ $[M+H]^+$ 350.2478, found 350.2482.

6a,8,8-Trimethyl-10-phenyl-6a,7,8,9-tetrahydro-6*H*-benzo[*c*]chromen-6-one (3ua):

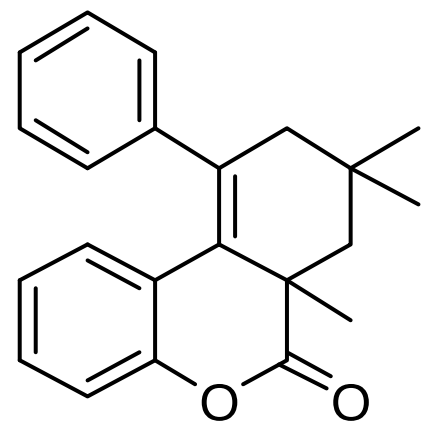

Yellow oil; ^1H NMR (400 MHz, $CDCl_3$) δ: 7.25-7.19 (m, 3H), 7.11 (t, *J* = 8.0 Hz, 1H), 7.01 (d, *J* = 7.6 Hz, 3H), 6.71 (t, *J* = 7.6 Hz, 1H), 6.55 (d, *J* = 7.6 Hz, 1H), 2.67 (d, *J* = 14.0 Hz, 1H), 2.49 (d, *J* = 16.8 Hz, 1H), 2.04 (d, *J* = 17.2 Hz, 1H), 1.55 (d, *J* = 14.4 Hz, 1H), 1.22 (s, 3H), 1.13 (s, 3H), 1.00 (s, 3H); ^{13}C NMR (100 MHz, $CDCl_3$) δ: 172.4, 150.0, 142.8, 139.1, 129.6, 128.5, 128.1, 127.0, 124.9, 123.2, 123.1, 115.8, 47.1, 43.8, 42.3, 30.1, 29.5, 26.5, 26.3; IR ν CO (KBr, cm^{-1}): 1758, 1457, 1093; LRMS (EI, 70 eV) m/z (%): 318 (M^+, 79), 275 (100), 213 (17); HRMS *m/z* (ESI) calcd for $C_{22}H_{23}O_2$ $[M+H]^+$ 319.1693, found 319.1699.

Methyl 7,7-dimethyl-5-phenyl-6,7,8,9-tetrahydro-8aH-fluorene-8a-carboxylate (3va):

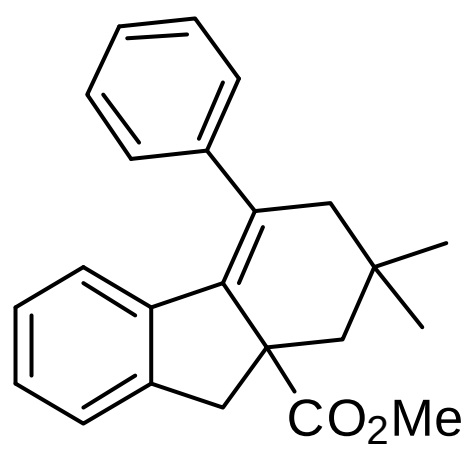

Yellow oil; ^1H NMR (400 MHz, $CDCl_3$) δ: 7.38-7.30 (m, 3H), 7.23 (d, *J* = 6.0 Hz, 2H), 7.12 (d, *J* = 6.8 Hz, 1H), 7.02 (t, *J* = 6.8 Hz, 1H), 6.83 (d, *J* = 7.6 Hz, 1H), 6.55 (d, *J* = 8.0 Hz, 1H), 3.57 (s, 3H), 3.31 (d, *J* = 15.6 Hz, 1H), 3.00 (d, *J* = 15.6 Hz, 1H), 2.56 (d, *J* = 13.2 Hz, 1H), 2.33 (d, *J* = 17.6 Hz, 1H), 2.15 (d, *J* = 17.6 Hz, 1H), 1.65 (d, *J* = 13.6 Hz, 1H), 1.05 (s, 6H); ^{13}C NMR (100 MHz, $CDCl_3$) δ: 177.5, 142.7, 142.2, 140.4, 136.1, 134.3, 129.1, 128.5, 128.1, 126.9, 126.1, 124.4, 123.6, 55.1, 52.1, 47.6, 45.5, 32.2, 30.9, 28.2, 26.2; IR ν CO (KBr, cm^{-1}): 1720, 1447, 1099; LRMS (EI, 70

eV) m/z (%): 332 (M^+, 86), 273 (100), 215 (50); HRMS *m/z* (ESI) calcd for $C_{24}H_{28}NO$ $[M+H]^+$ 333.1849, found 333.1854.

Methyl 7,7-diethyl-5-phenyl-6-vinyl-6,7,8,9-tetrahydro-8aH-fluorene-8a-carboxylate (3vc):

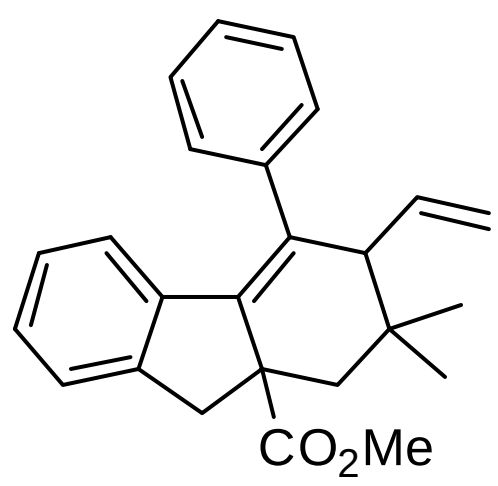

Yellow oil; ^1H NMR (400 MHz, $CDCl_3$) δ: 7.36-7.27 (m, 4H), 7.19-7.15 (m, 1H), 7.12 (d, *J* = 7.6 Hz, 1H), 7.01 (t, *J* = 7.2 Hz, 1H), 6.80 (t, *J* = 7.6 Hz, 1H), 6.42 (d, *J* = 7.6 Hz, 1H), 5.90-5.81 (m, 1H), 5.01 (dd, *J* = 10.0, 1.6 Hz, 1H), 4.87 (dd, *J* = 17.2, 2.0 Hz, 1H), 3.59 (s, 3H), 3.43 (d, *J* = 15.2 Hz, 1H), 3.02 (d, *J* = 15.2 Hz, 1H), 2.64 (d, *J* = 9.6 Hz, 1H), 2.35 (d, *J* = 14.0 Hz, 1H), 1.88 (d, *J* = 14.0 Hz, 1H), 1.12 (s, 3H), 0.97 (s, 3H); ^{13}C NMR (100 MHz, $CDCl_3$) δ: 177.8, 142.0, 141.9, 140.3, 136.9, 136.6, 135.9, 128.5, 128.2, 127.1, 126.8, 126.2, 124.3, 123.7, 117.4, 60.2, 55.2, 52.3, 44.3, 42.7, 34.9, 29.8, 26.4; IR ν CO (KBr, cm^{-1}): 1722, 1449, 1096; LRMS (EI, 70 eV) m/z (%): 358 (M^+, 100), 299 (54), 239 (16); HRMS *m/z* (ESI) calcd for $C_{27}H_{31}O_2[M+H]^+$ 387.2319, found 387.2317.

2,2,3a,5-Tetramethyl-1-phenyl-2,3,3a,5-tetrahydro-4*H*-cyclopenta[*c*]quinolin-4-one (4ad):

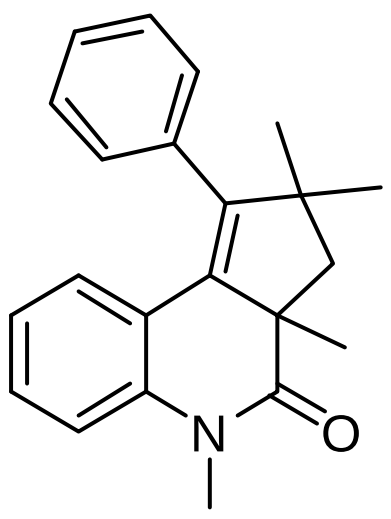

Yellow oil; ^1H NMR (400 MHz, $CDCl_3$) δ: 7.33 (d, *J* = 6.4 Hz, 3H), 7.17-7.12 (m, 3H), 6.98 (d, *J* = 8.4 Hz, 1H), 6.75-6.68 (m, 2H), 3.40 (s, 3H), 2.58 (d, *J* = 13.6 Hz, 1H), 2.08 (d, *J* = 13.6 Hz, 1H), 1.40 (s, 3H), 1.34 (s, 3H), 0.97 (s, 3H); ^{13}C NMR (100 MHz, $CDCl_3$) δ: 175.8, 148.1, 139.9, 136.7, 132.8, 129.0, 128.3, 127.9, 127.5, 127.1, 122.2, 121.7, 114.7, 51.7, 49.5, 47.8, 29.9, 29.6, 29.2, 26.3; IR ν CO (KBr, cm^{-1}): 1677, 1596, 1455; LRMS (EI, 70 eV) m/z (%): 317 (M^+, 35), 202 (100); HRMS *m/z* (ESI) calcd for $C_{22}H_{24}NO$ $[M+H]^+$ 318.1852, found 318.1851.

3a',5'-Dimethyl-1'-phenyl-3',3a'-dihydrospiro[cyclopentane-1,2'-cyclopenta[*c*]quinolin]-4'(5'*H*)-one (4ae):

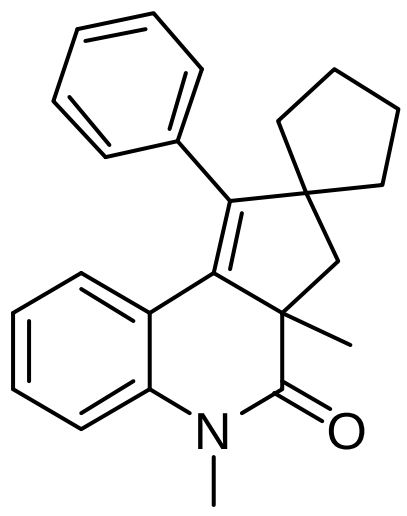

Yellow oil; ^{1}H NMR (400 MHz, $CDCl_3$) δ: 7.33 (d, *J* = 6.8 Hz, 3H), 7.13 (d, *J* = 6.8 Hz, 3H), 6.98 (d, *J* = 8.4 Hz, 1H), 6.75 (d, *J* = 7.6 Hz, 1H), 6.69 (t, *J* = 7.6 Hz, 1H), 3.40 (s, 3H), 2.36 (d, *J* = 14.4 Hz, 1H), 2.07 (d, *J* = 13.2 Hz, 1H), 1.88-1.80 (m, 1H), 1.73-1.52 (m, 4H), 1.45-1.36 (m, 1H), 1.34-1.18 (m, 5H); ^{13}C NMR (100 MHz, $CDCl_3$) δ: 175.8, 146.0, 139.8, 136.9, 133.9, 129.4, 128.3, 127.9 127.4, 127.1, 122.2, 121.6, 114.7, 58.8, 52.0, 48.9, 39.0, 38.4, 29.9, 26.5, 24.2, 23.9; IR ν CO (KBr, cm^{-1}): 1683, 1593, 1457; LRMS (EI, 70 eV) m/z (%): 343 (M^+, 100), 300 (44), 285 (10); HRMS *m/z* (ESI) calcd for $C_{24}H_{26}NO$ $[M+H]^+$ 344.2009, found 344.2013.

3a',5'-Dimethyl-1'-phenyl-3',3a'-dihydrospiro[cyclohexane-1,2'-cyclopenta[*c*]quinolin]-4'(5'*H*)-one (4af):

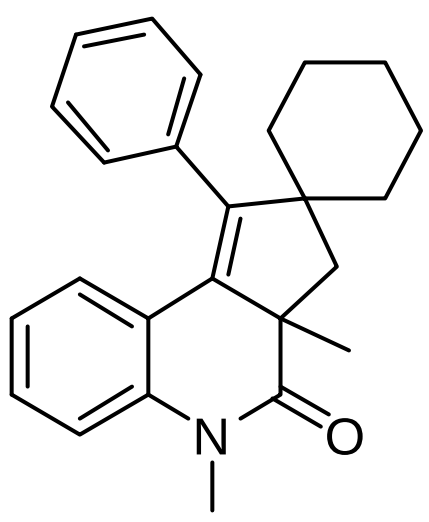

Yellow oil; ^{1}H NMR (400 MHz, $CDCl_3$) δ: 7.33 (d, *J* = 6.4 Hz, 3H), 7.16-7.09 (m, 3H), 6.97 (d, *J* = 8.0 Hz, 1H), 6.69 (d, *J* = 4.0 Hz, 2H), 3.40 (s, 3H), 2.46 (d, *J* = 14.0 Hz, 1H), 2.28 (d, *J* = 13.6 Hz, 1H), 1.68 (d, *J* = 6.4 Hz, 4H), 1.62-1.40 (m, 4H), 1.32 (s, 3H), 1.17-1.09 (m, 1H), 1.01-0.92 (m, 1H); ^{13}C NMR (100 MHz, $CDCl_3$) δ: 175.9, 148.7, 139.8, 136.8, 133.2, 129.5, 128.1, 127.8, 127.5, 127.0, 122.2, 121.8, 114.6, 52.2, 51.9, 43.6, 37.6, 35.9, 30.0, 27.3, 25.4, 23.5, 22.4; IR ν CO (KBr, cm^{-1}): 1681, 1597, 1458; LRMS (EI, 70 eV) m/z (%): 357 (M^+, 100), 286 (35); HRMS *m/z* (ESI) calcd for $C_{25}H_{28}NO$ $[M+H]^+$ 358.2165, found 358.2166.

3A,5-dimethyl-1-phenyl-3,3a-dihydro-2*H*-cyclopenta[*c*]quinoline-2,4(5*H*)-dione (5):

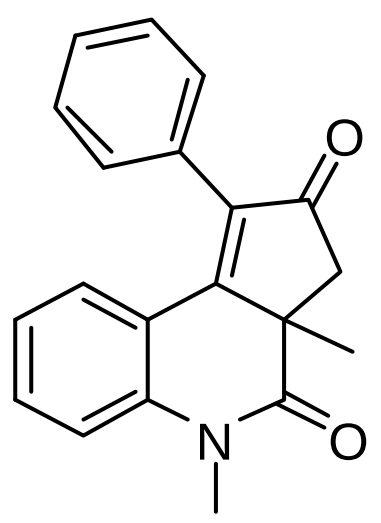

Yellow oil; ^{1}H NMR (400 MHz, $CDCl_3$) δ: 7.48-7.33 (m, 4H), 7.29 (d, *J* = 6.8 Hz, 2H), 7.22 (d, *J* = 7.6 Hz, 1H), 7.16 (d, *J* = 8.4 Hz, 1H), 6.93 (t, *J* = 7.6 Hz, 1H), 3.48 (s, 3H), 3.29 (d, *J* = 18.8 Hz, 1H), 2.66 (d, *J* = 18.8 Hz, 1H), 1.42 (s, 3H); ^{13}C NMR (100 MHz, $CDCl_3$) δ: 203.7, 172.3, 164.1, 139.9, 136.7, 131.9, 130.6, 129.1, 128.6, 128.5, 128.4, 122.8, 119.4, 115.5, 48.0, 45.2, 30.3, 27.1; IR ν CO (KBr, cm^{-1}): 1720, 1681, 1597; LRMS (EI, 70 eV) m/z (%): 303 (M^+, 100), 275 (20), 217 (16); HRMS *m/z* (ESI) calcd for $C_{20}H_{17}NO_2$ $[M+H]^+$ 304.1332, found 304.1336.

1-(*tert*-Butoxy)-2,2,6,6-tetramethylpiperidine (6):

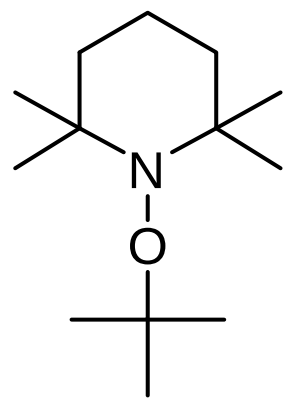

Colourless oil; ^{1}H NMR (400 MHz, $CDCl_3$) δ: 1.59-1.37 (m, 6H), 1.28 (s, 9H), 1.12 (s, 6H), 1.07 (s, 6H); ^{13}C NMR (100 MHz, $CDCl_3$) δ: 59.1, 40.9, 34.8, 29.4, 20.4, 17.2; IR ν CO (KBr, cm^{-1}): 1724, 1680, 1595; LRMS (EI, 70 eV) m/z (%): 213 (M^+, 1), 157 (20), 142 (100); HRMS *m/z* (ESI) calcd for $C_{13}H_{27}NO$ $[M+H]^+$ 214.2165, found 214.2169.

5-Methyl-12-phenylbenzo[*j*]phenanthridin-6(5*H*)-one (7):

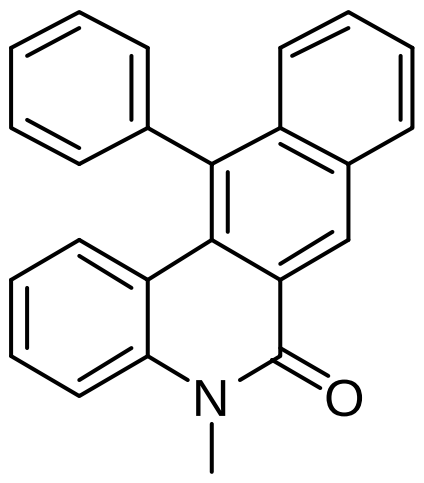

Yellow oil; ^{1}H NMR (400 MHz, $CDCl_3$) δ: 9.28 (s, 1H), 8.12 (d, *J* = 8.0 Hz, 1H), 7.66-7.51 (m, 5H), 7.51-7.44 (m, 1H), 7.43-7.29 (m, 4H), 7.24 (d, *J* = 10.8 Hz, 1H), 6.78-6.74 (m, 1H), 3.82 (s, 3H); ^{13}C NMR (100 MHz, $CDCl_3$) δ: 162.3, 140.9, 138.6, 136.5, 135.5, 131.9, 130.5 (2C), 129.6, 129.4, 129.1, 128.5, 128.1, 127.9, 127.6, 126.9, 126.4, 124.6, 121.4, 120.6, 114.8, 30.6; IR ν CO (KBr, cm^{-1}): 1724, 1680, 1595; LRMS (EI, 70 eV) m/z (%): 335 (M^+, 100), 207 (10), 167 (12); HRMS *m/z* (ESI) calcd for $C_{24}H_{17}NO$ $[M+H]^+$ 336.1383, found 336.1387.

(C) Reference

[1] X. Mu, T. Wu, H.-y. Wang, Y.-l. Guo, G. Liu *J. Am. Chem. Soc.* **2012**, *134*, 878; N. Sakiyama, K. Noguchi, K. Tanaka, *Angew. Chem. Int. Ed.* **2012**, *51*, 5976.

(D) Additional experiments

(1) A mixture were obtained when 2-benzyl-*N*-methyl-*N*-(2-(phenylethynyl)-phenyl)-acrylamide was used as the substrate. Compounds **8** and **9** could not be separated by silica gel flash column chromatography.

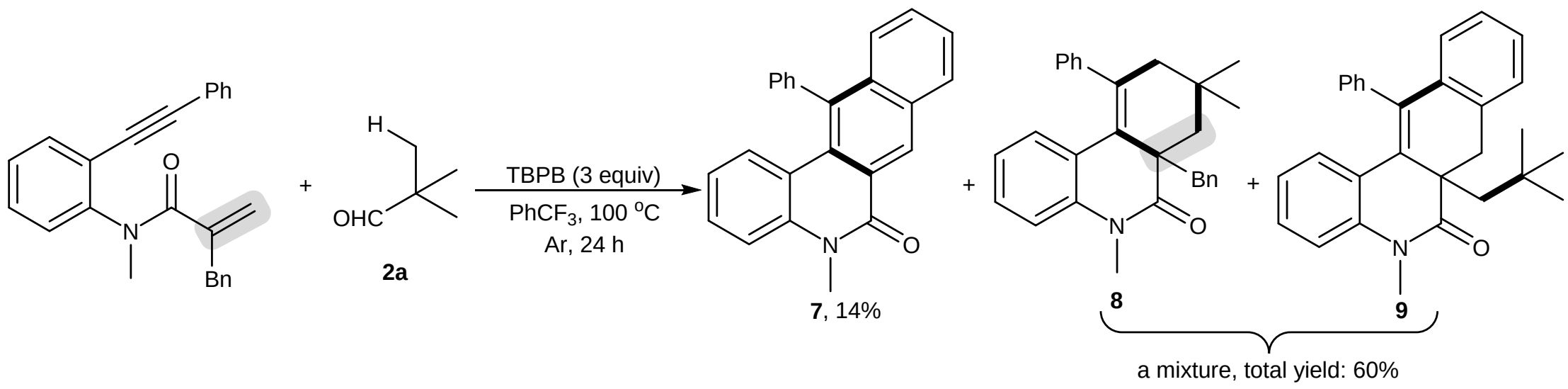

GC-MS anylysis of compounds **8** and **9**

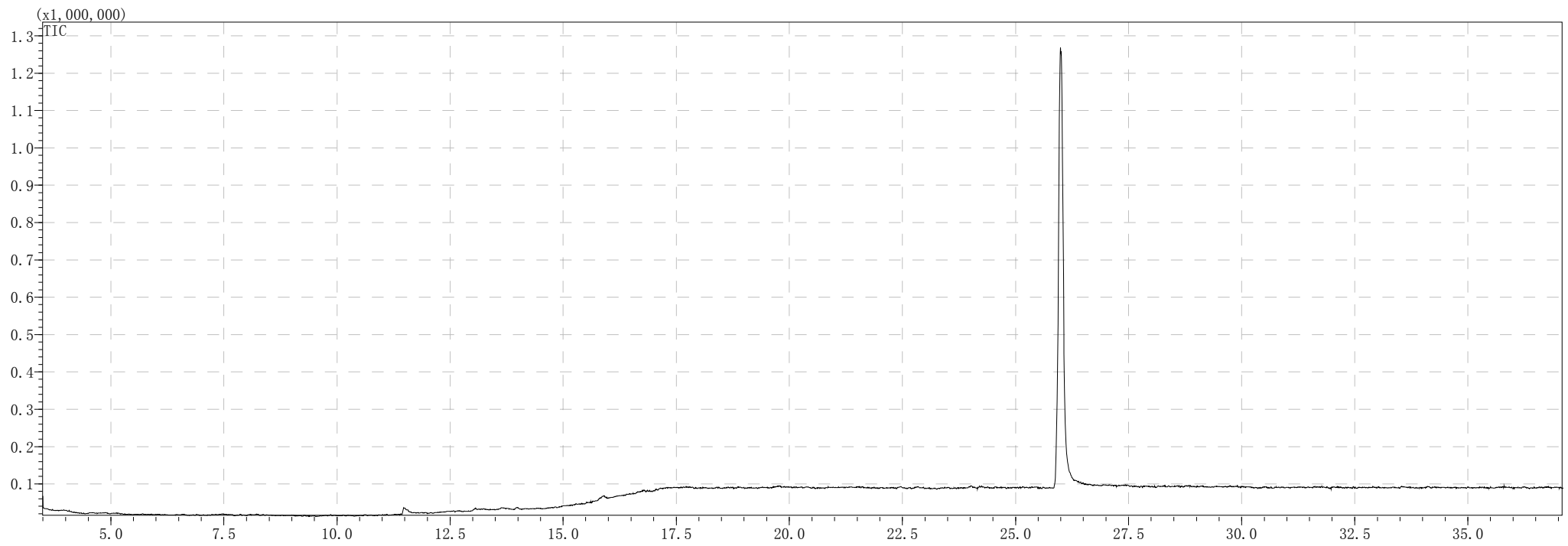

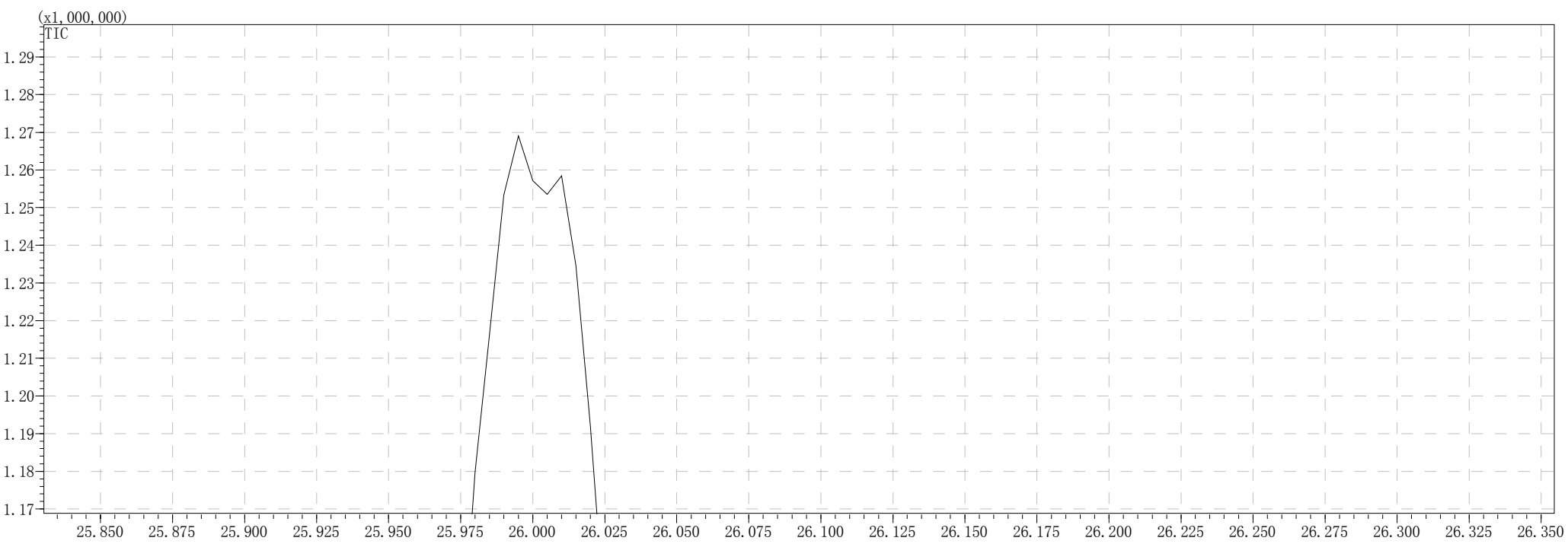

^{1}H NMR of compounds **8** and **9**

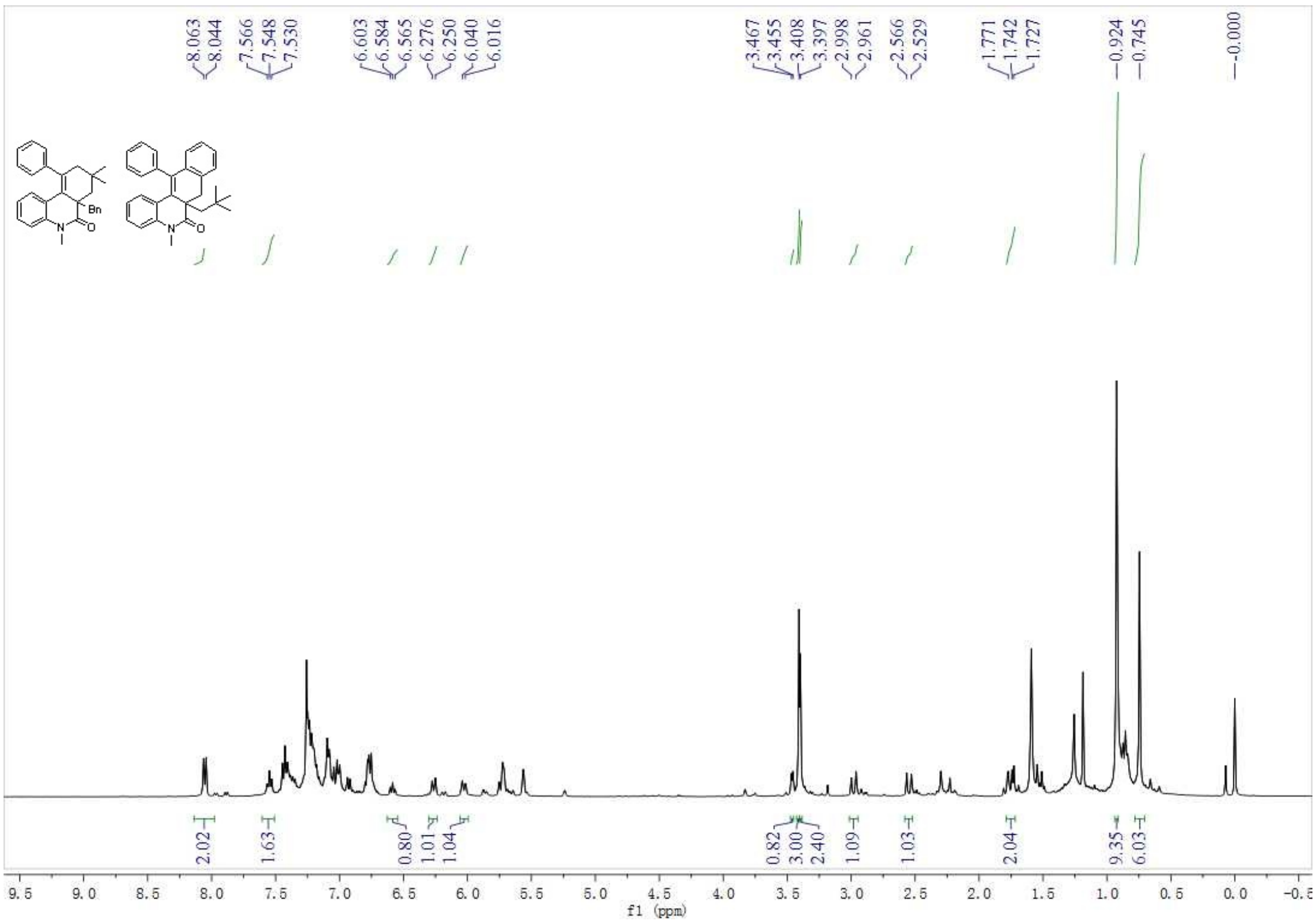

(2) 4-Methyl-*N*-(2-methylallyl)-*N*-(3-phenylprop-2-yn-1-yl)-benzenesulfonamide (**1w**) was not a suitable substrate under the optimal conditions. The result showed that the phenyl tether between the double bond and alkyne was essential.

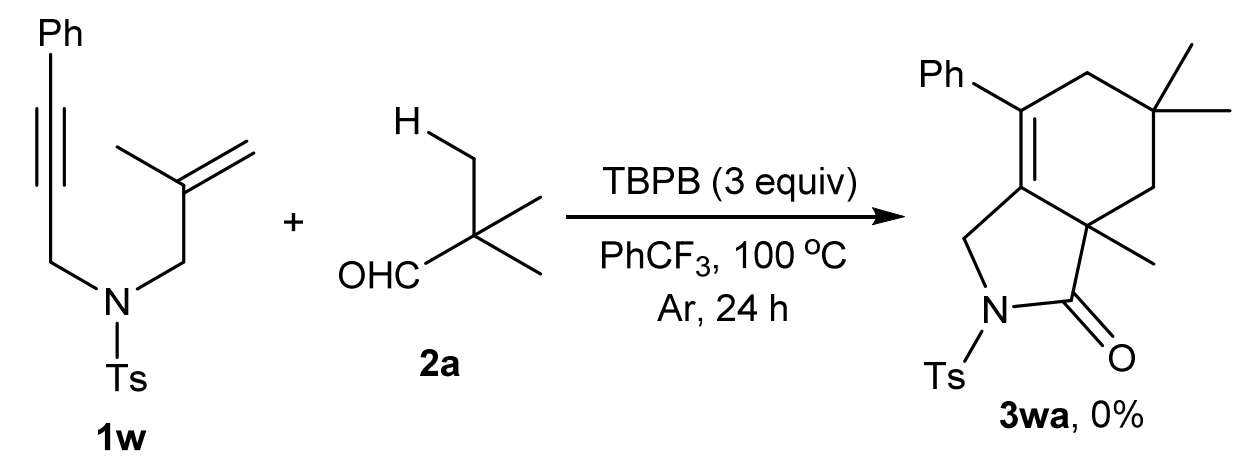

(E)Spectra

5,6a,8,8-Tetramethyl-10-phenyl-6a,7,8,9-tetrahydrophenanthridin-6(5*H*)-one (3aa)

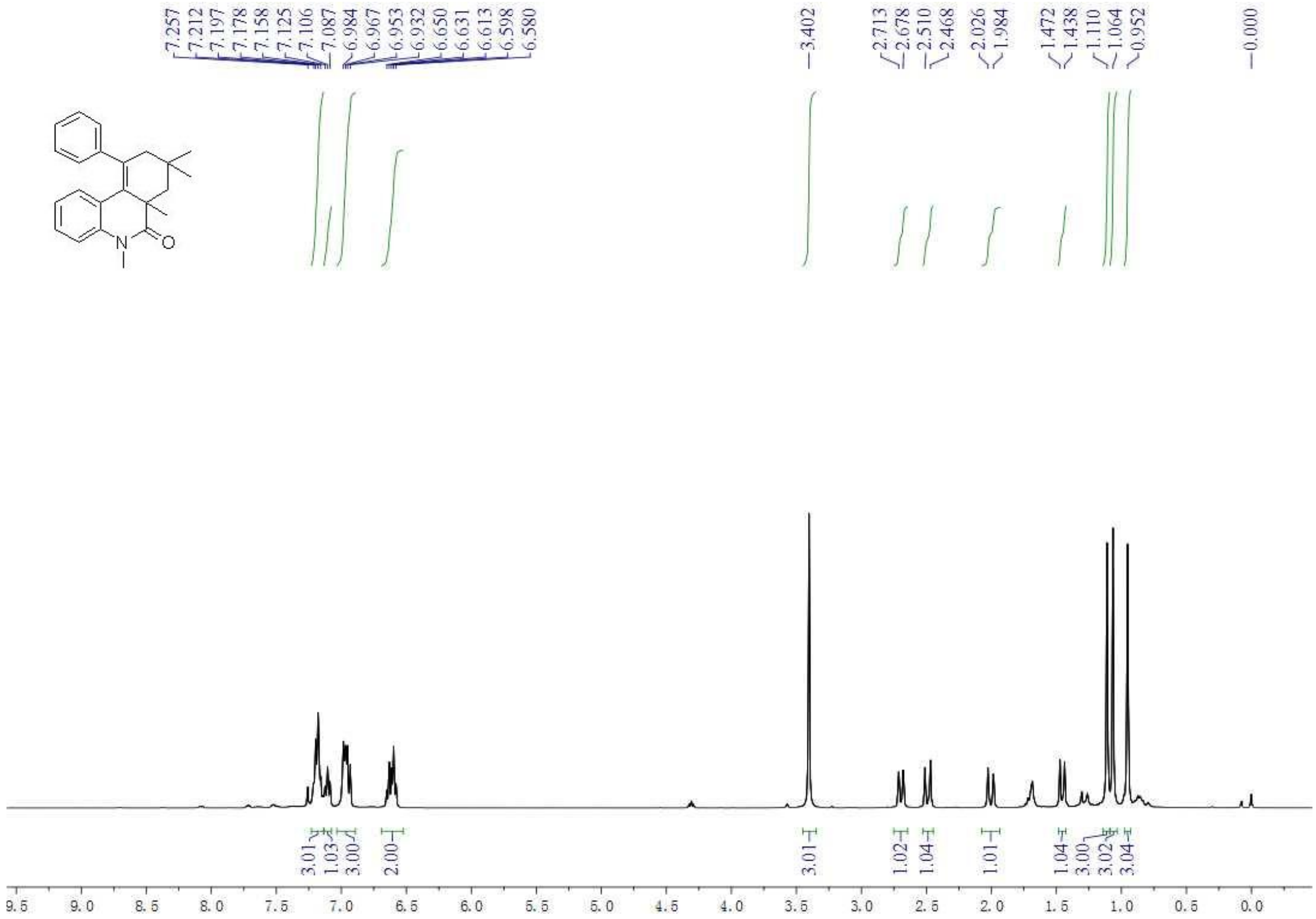

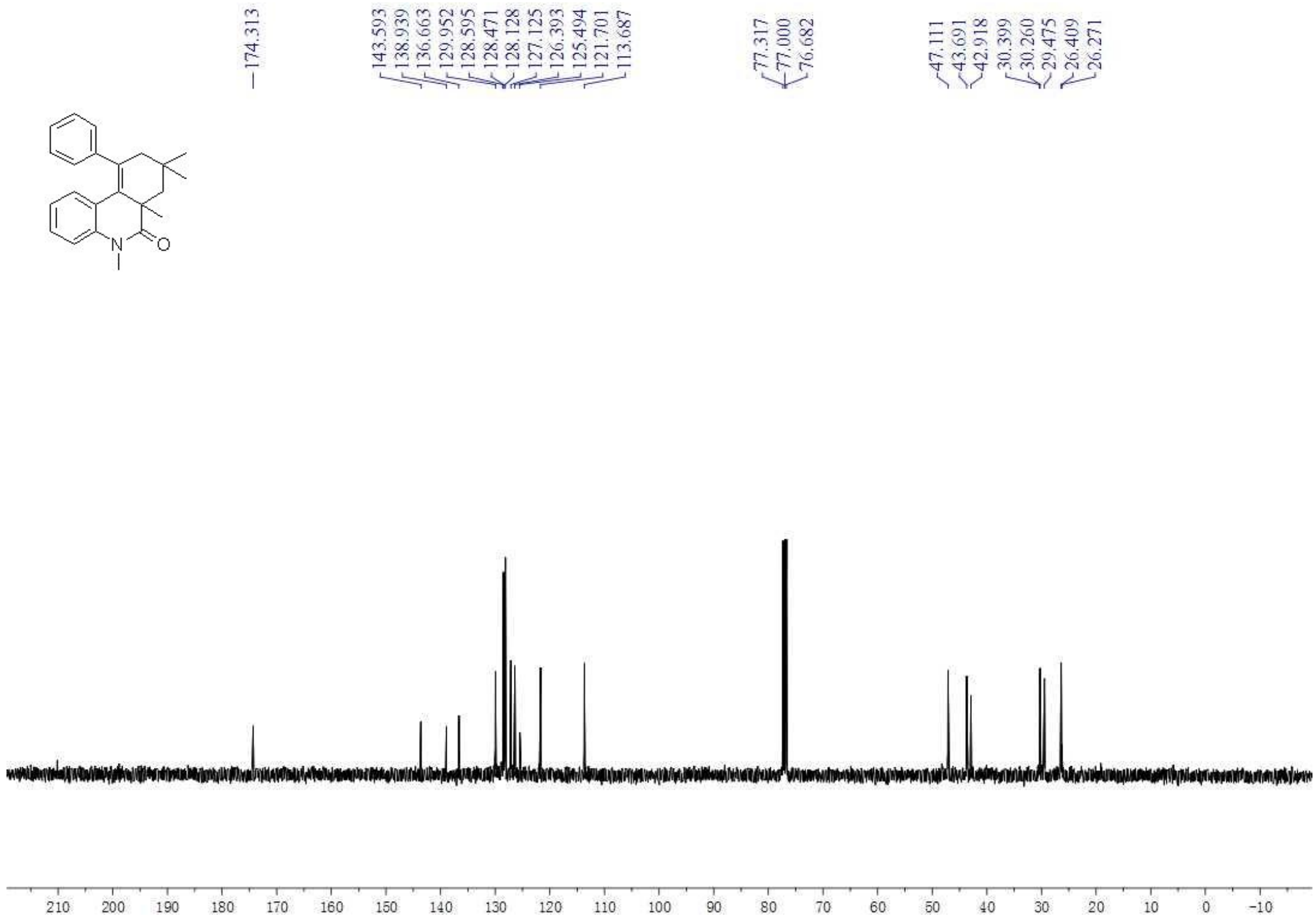

5-Benzyl-6a,8,8-trimethyl-10-phenyl-6a,7,8,9-tetrahydrophenanthridin-6(5*H*)-one (3ba)

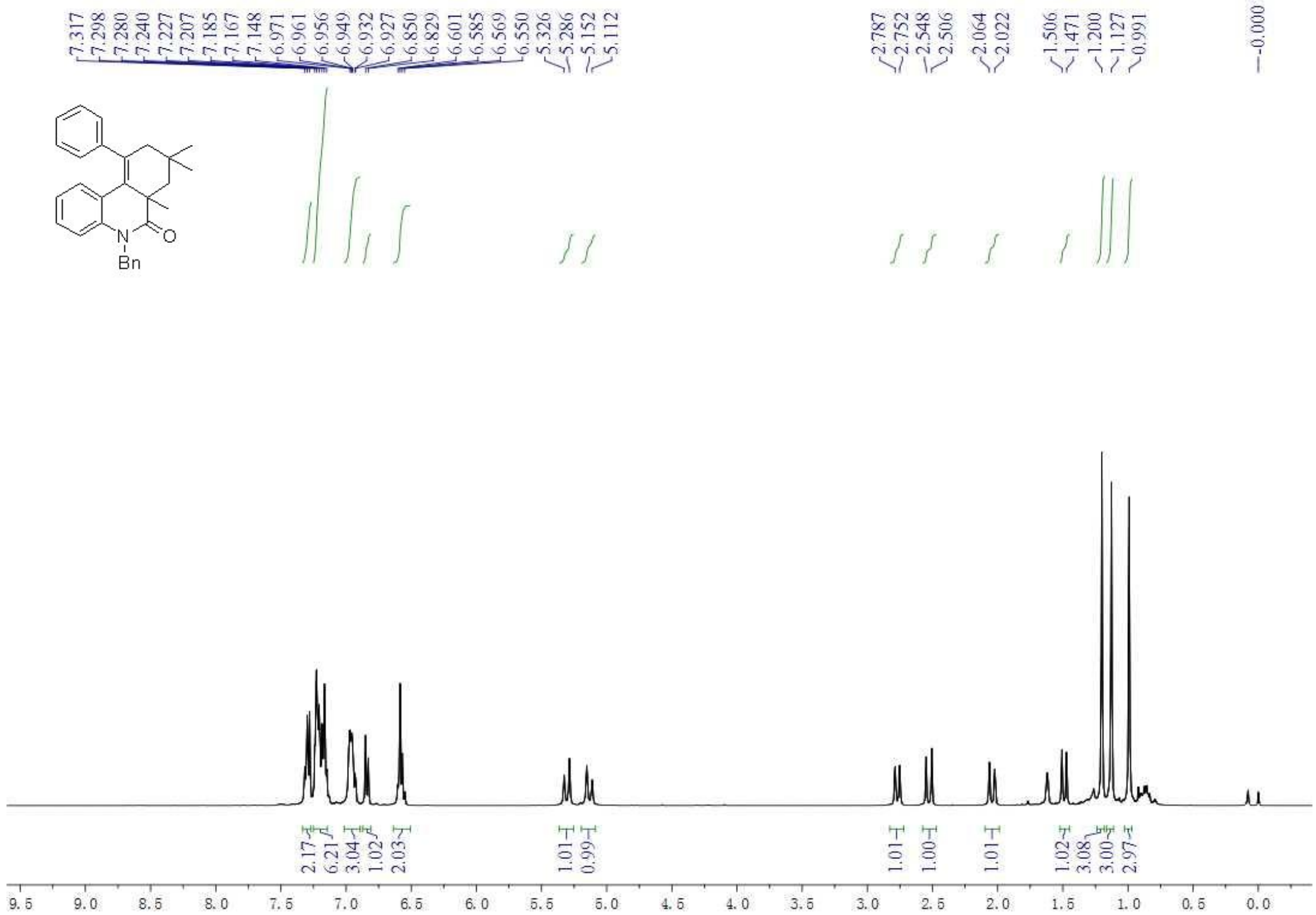

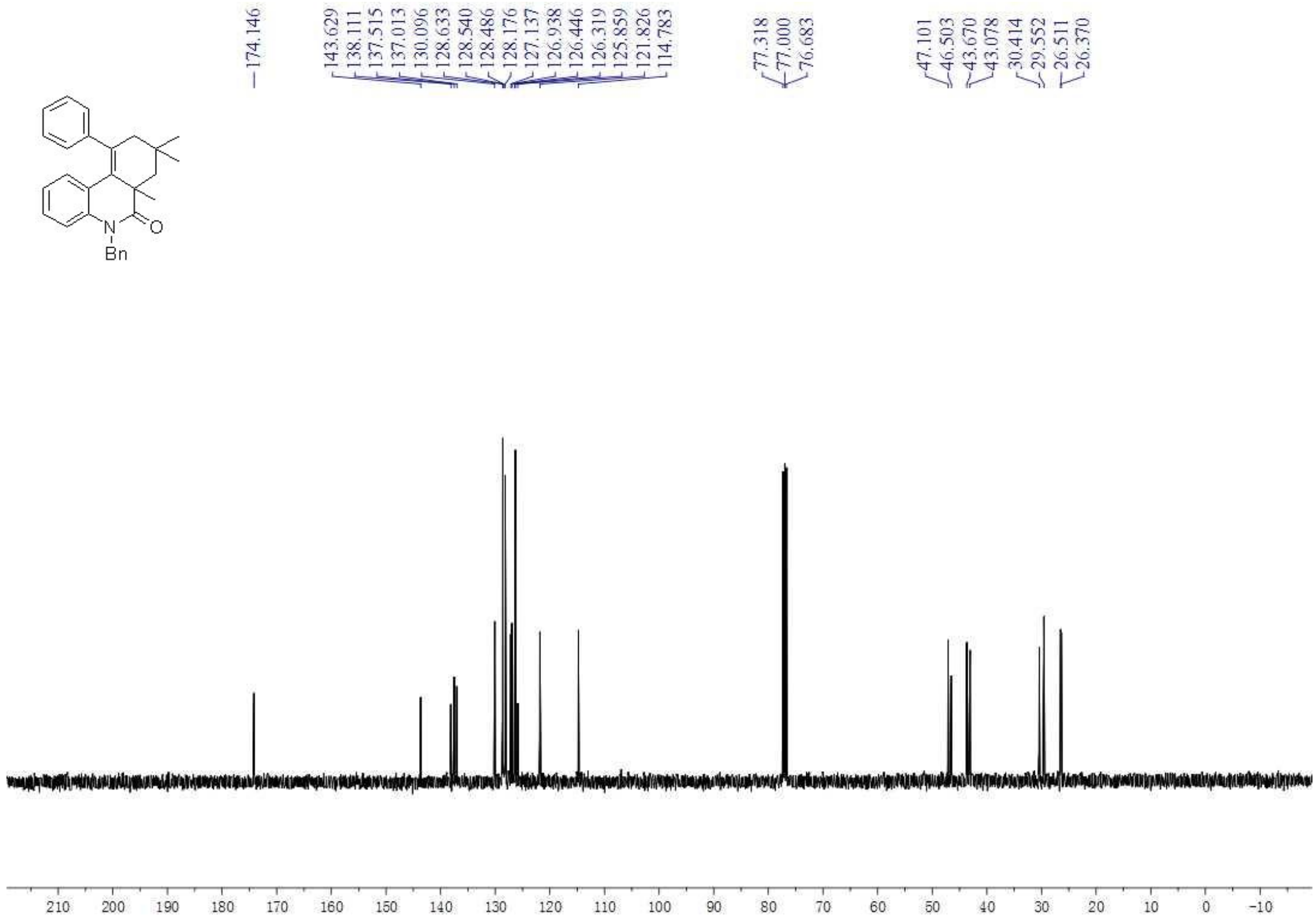

5-Allyl-6a,8,8-trimethyl-10-phenyl-6a,7,8,9-tetrahydrophenanthridin-6(5*H*)-one (3ca)

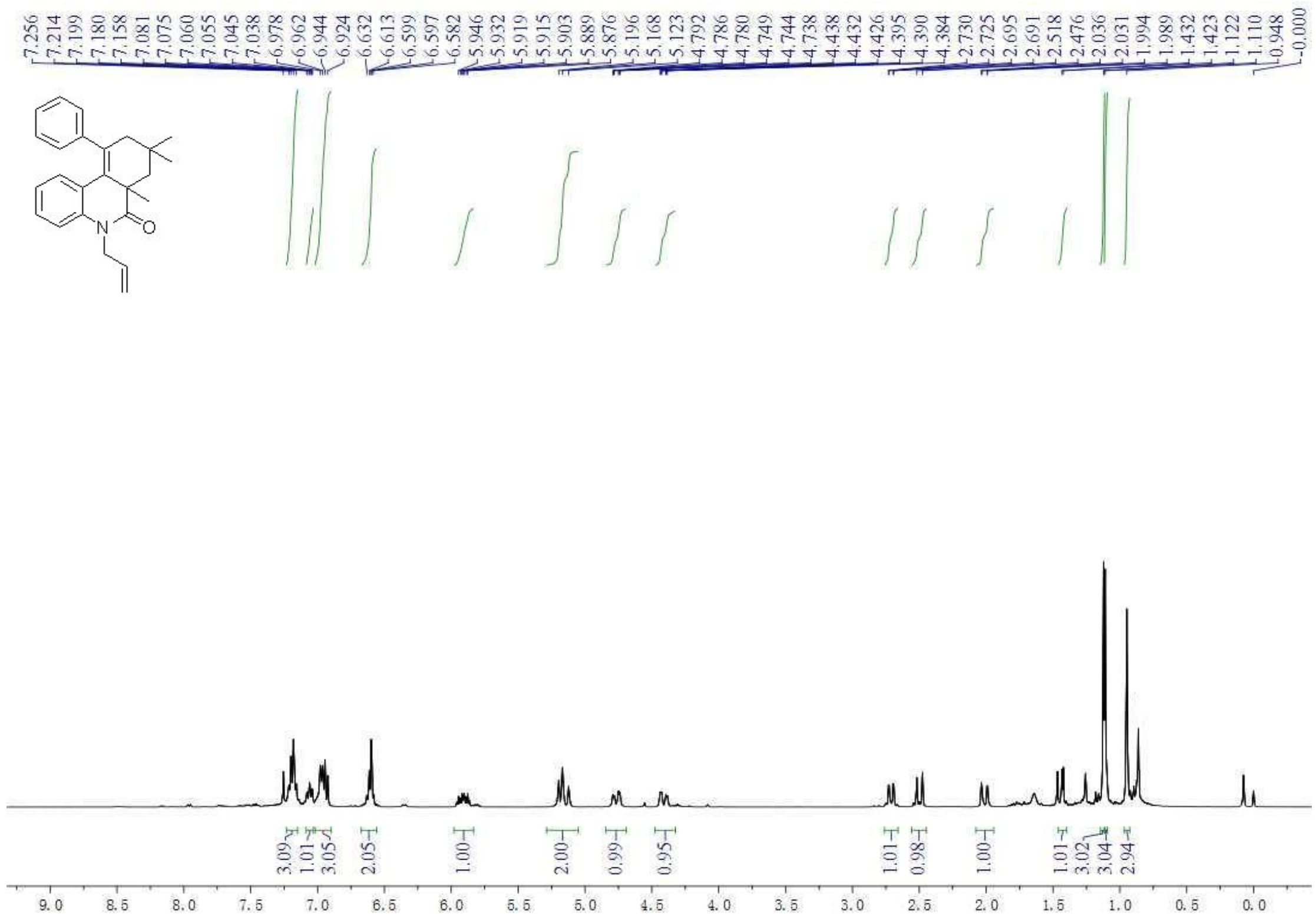

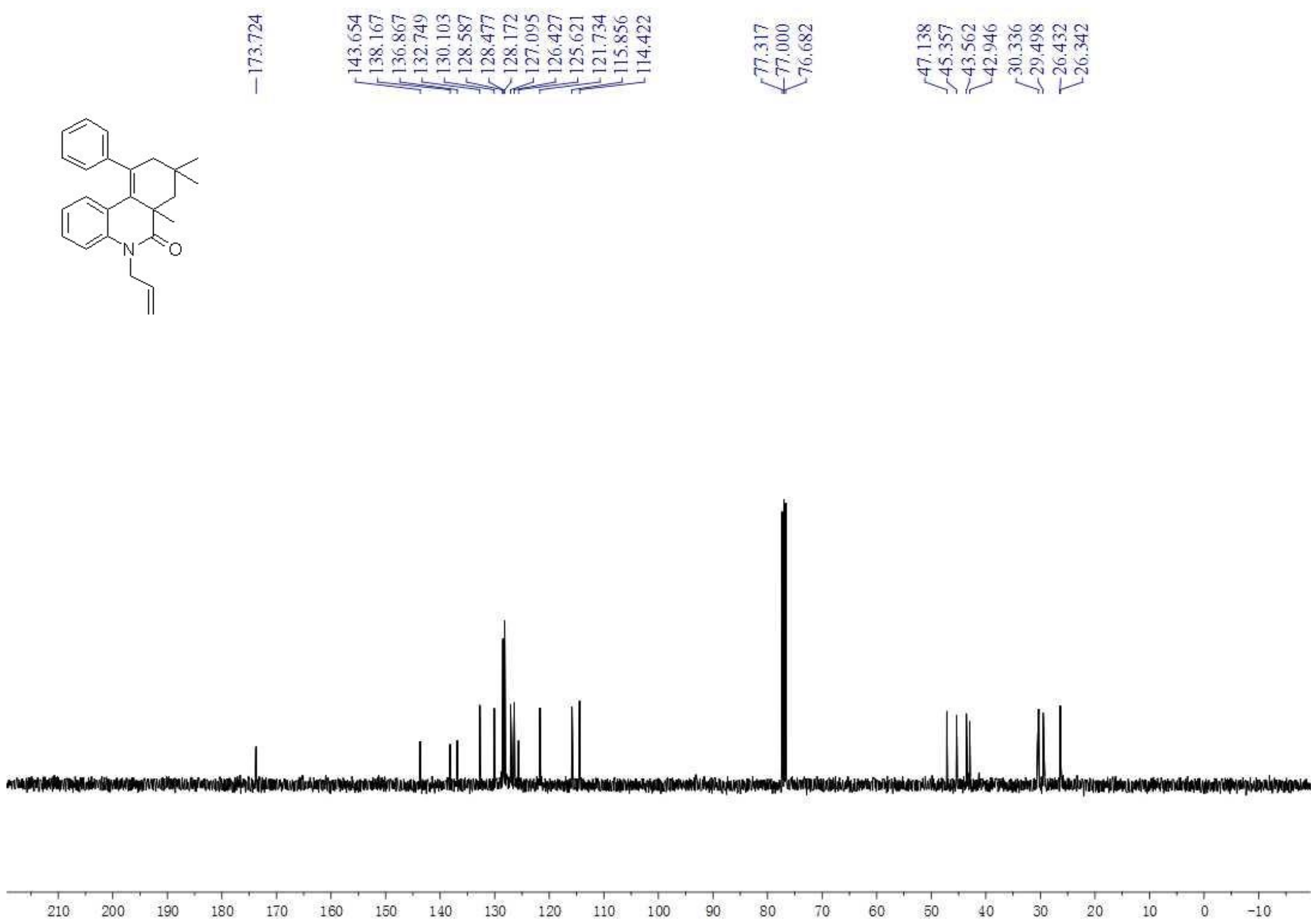

6a,8,8-Trimethyl-10-phenyl-5-tosyl-6a,7,8,9-tetrahydrophenanthridin-6(5*H*)-one (3da)

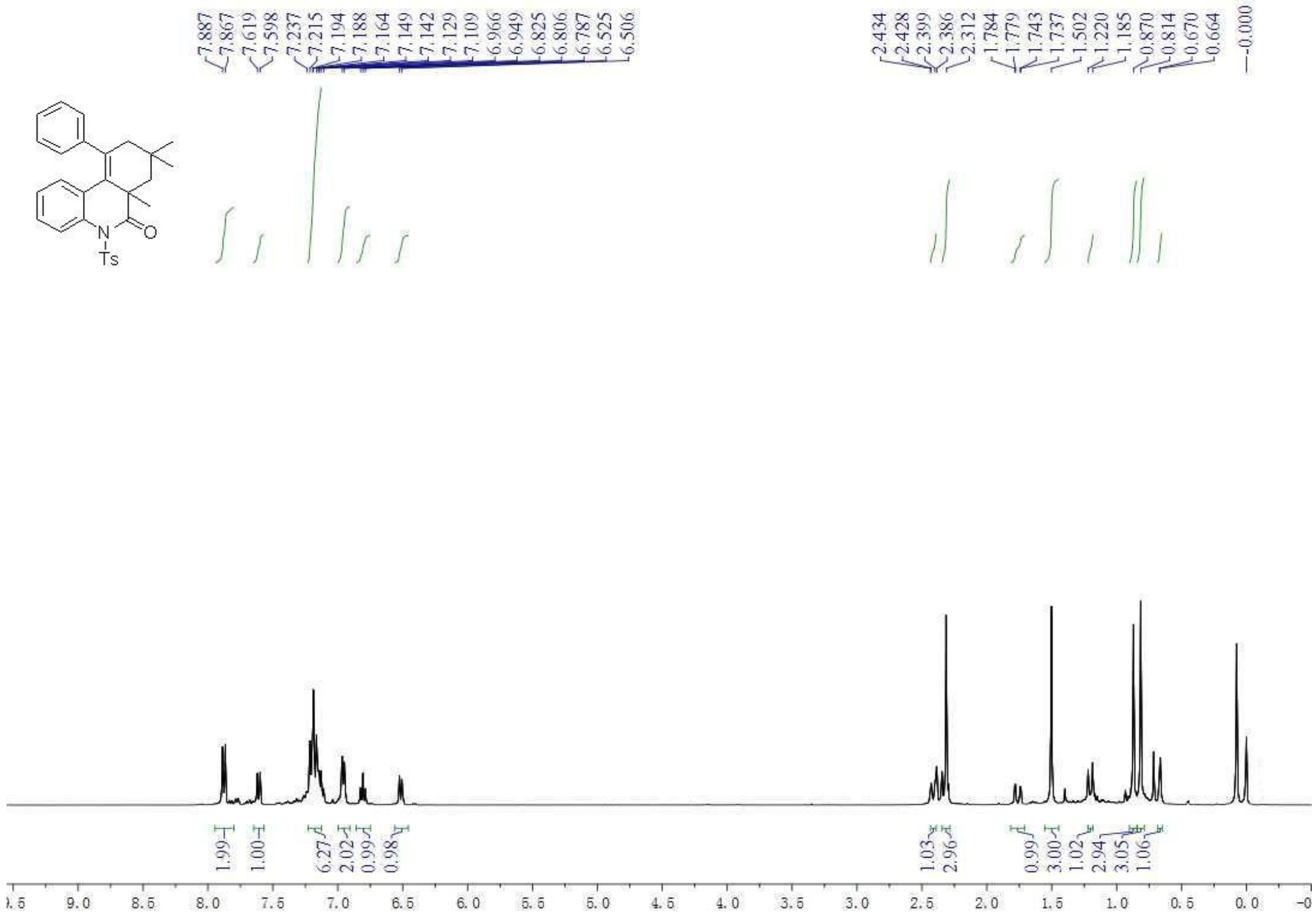

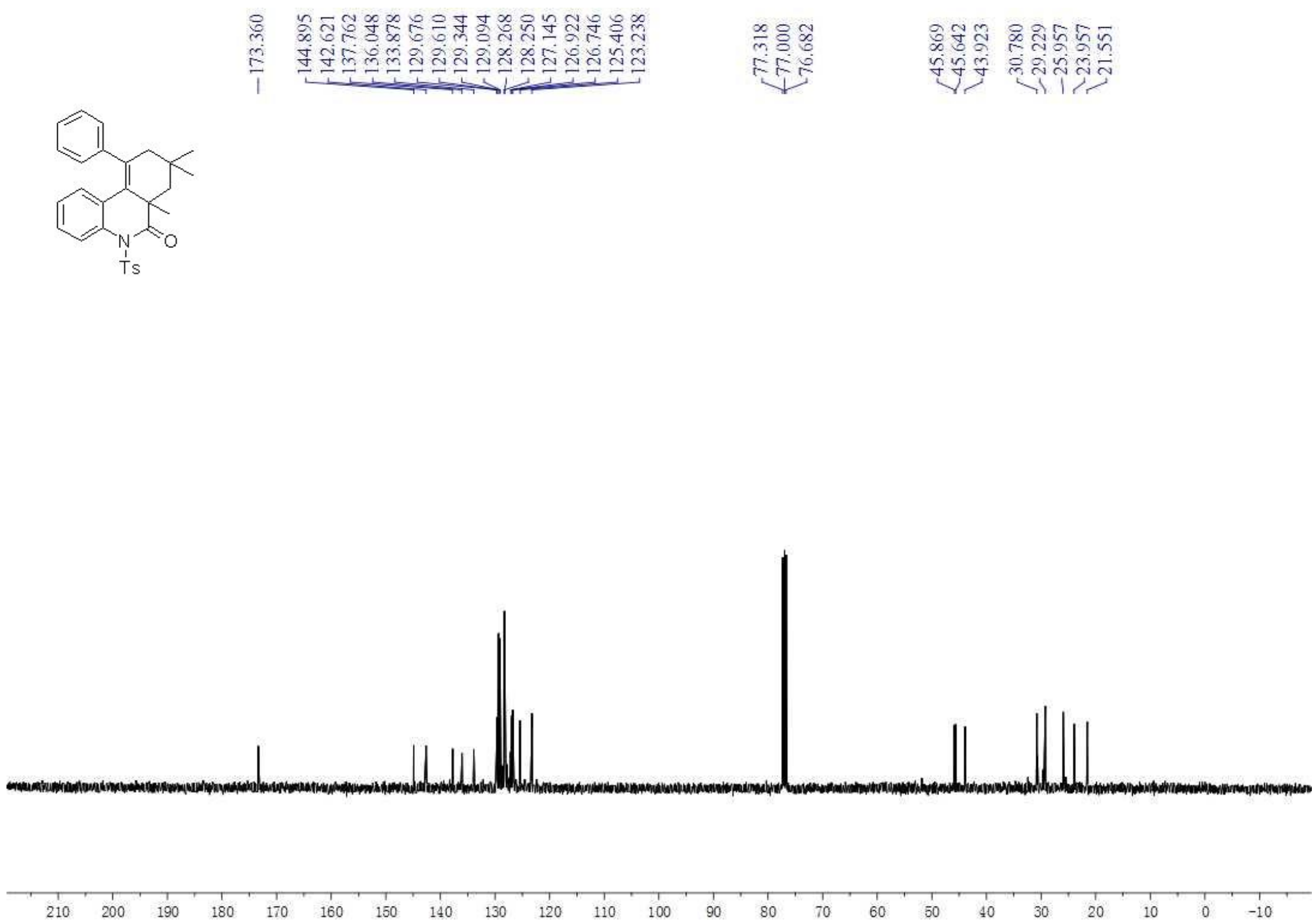

6a,8,8-Trimethyl-10-phenyl-6a,7,8,9-tetrahydrophenanthridin-6(5*H*)-one (3ea)

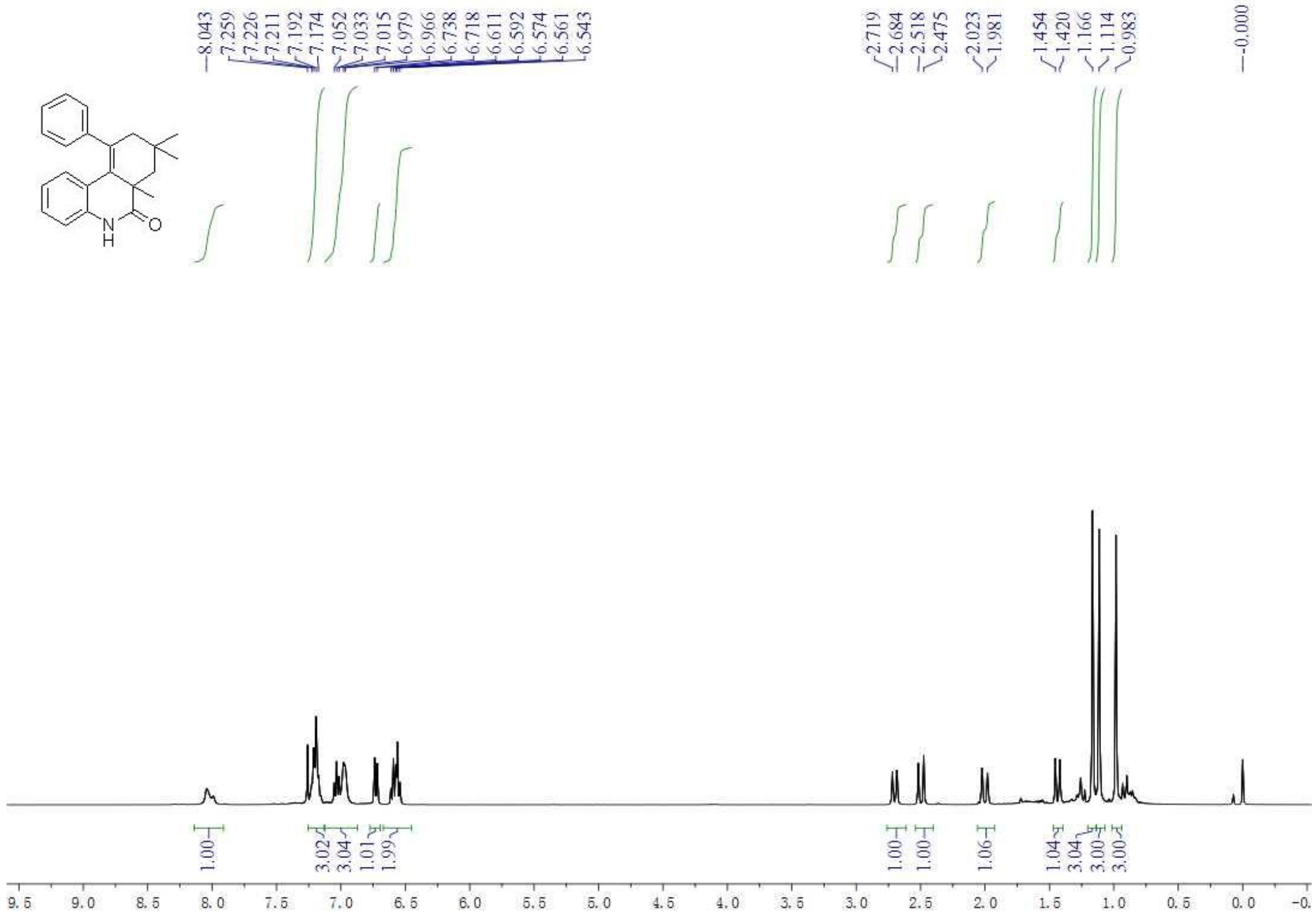

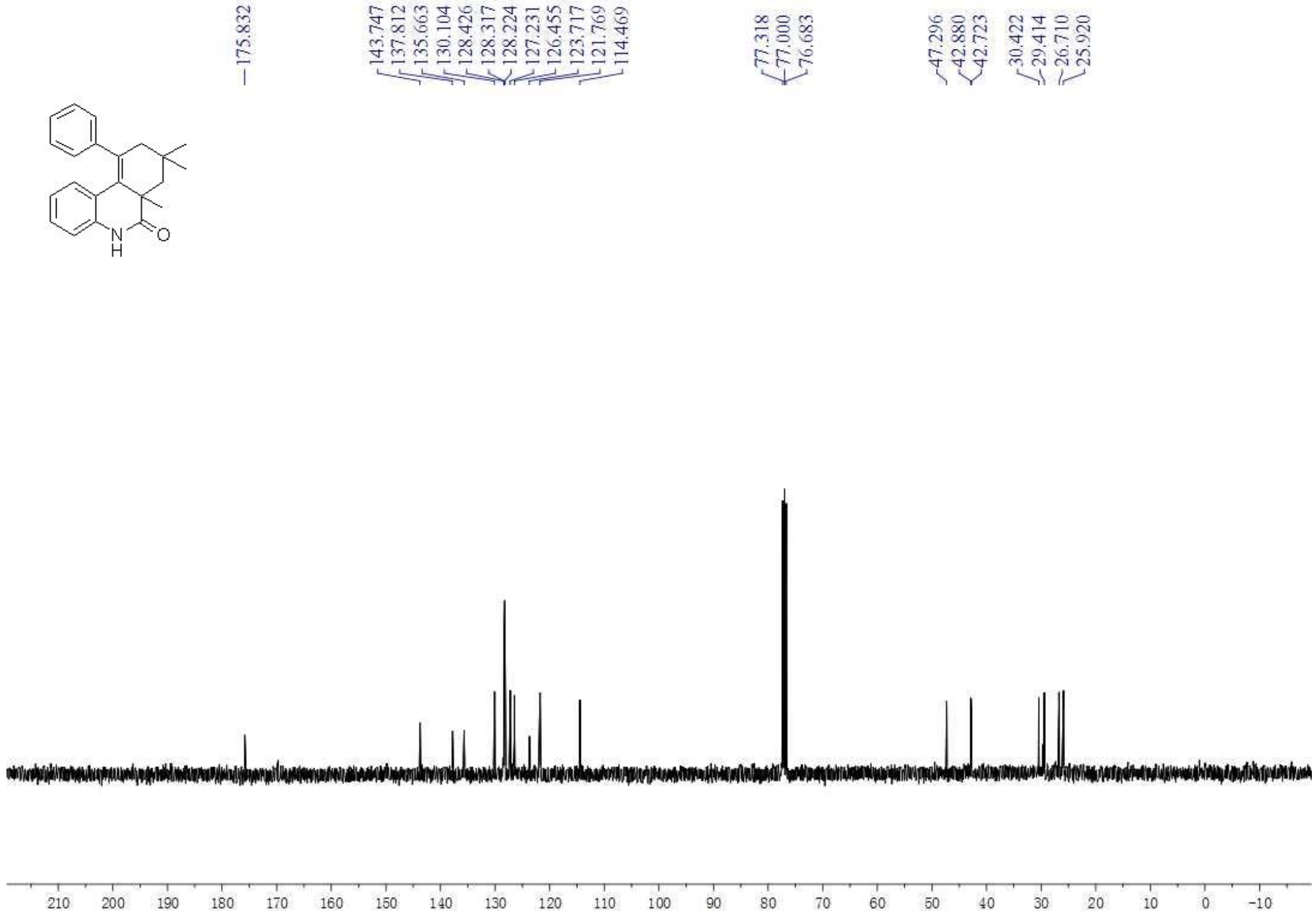

5,6a,8,8-Tetramethyl-10-(*p*-tolyl)-6a,7,8,9-tetrahydrophenanthridin-6(5*H*)-one (3fa)

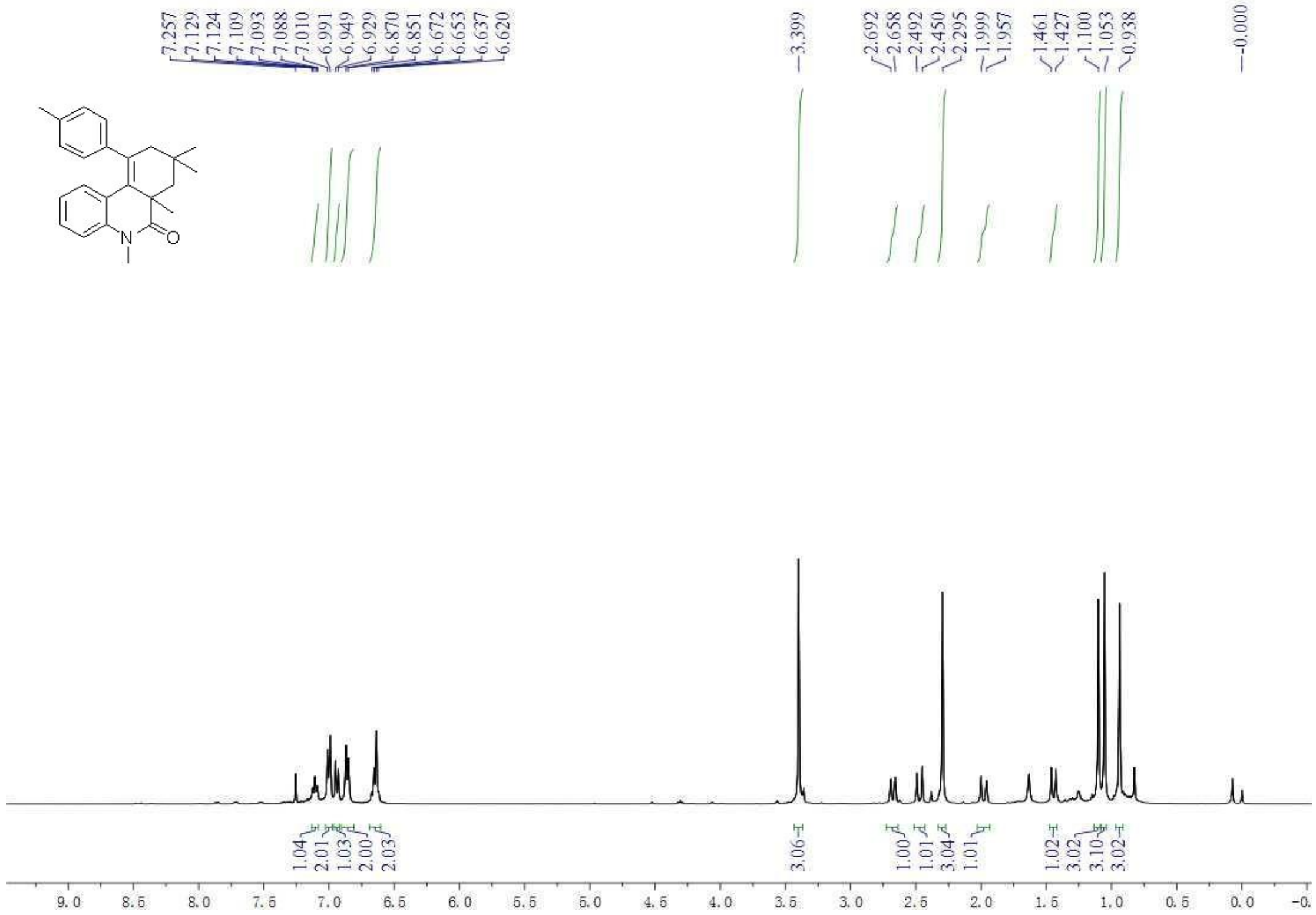

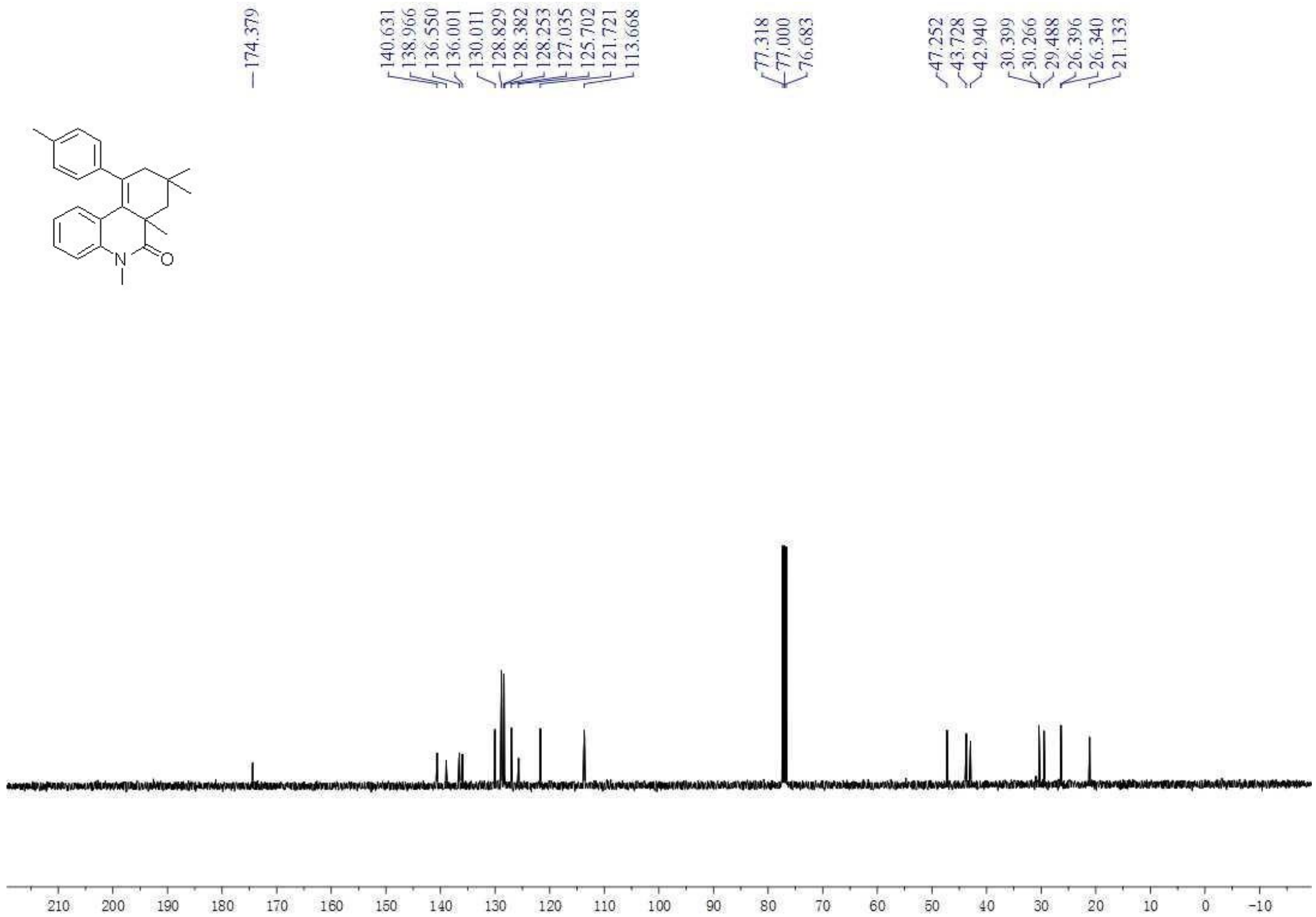

10-(4-Methoxyphenyl)-5,6a,8,8-tetramethyl-6a,7,8,9-tetrahydrophenanthridin-6(5*H*)-one (3ga)

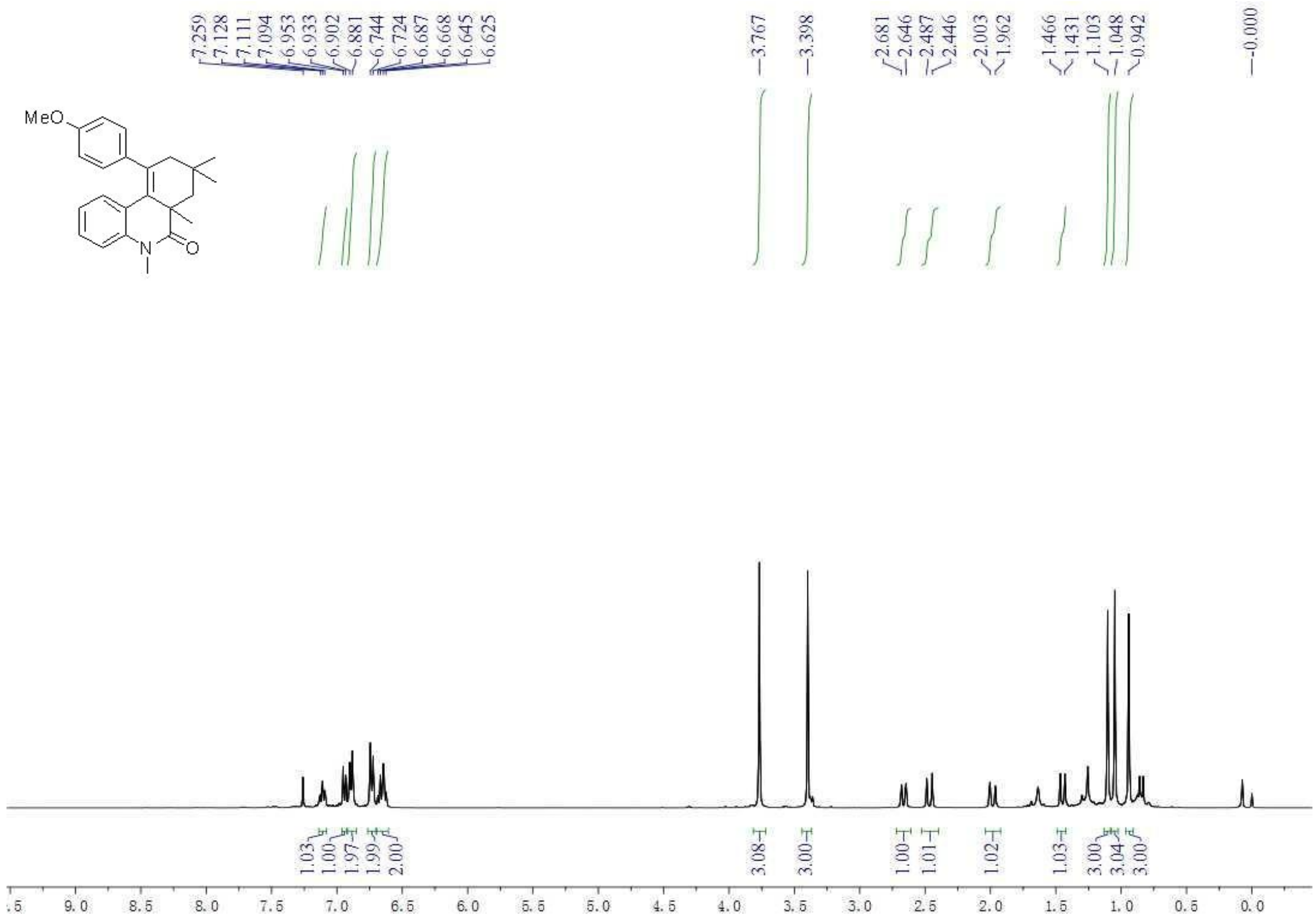

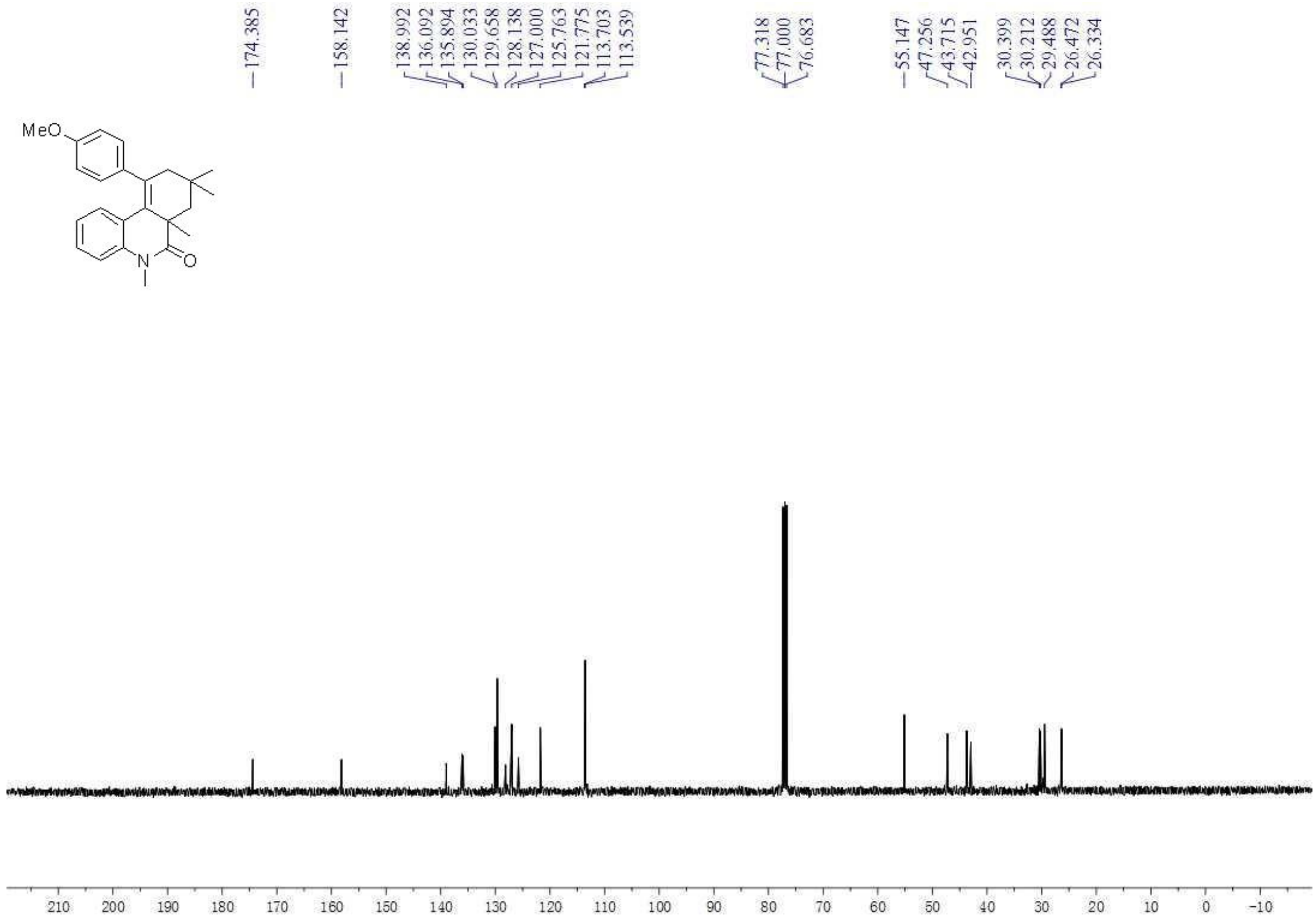

10-(4-Bromophenyl)-5,6a,8,8-tetramethyl-6a,7,8,9-tetrahydrophenanthridin-6(5*H*)-one (3ha)

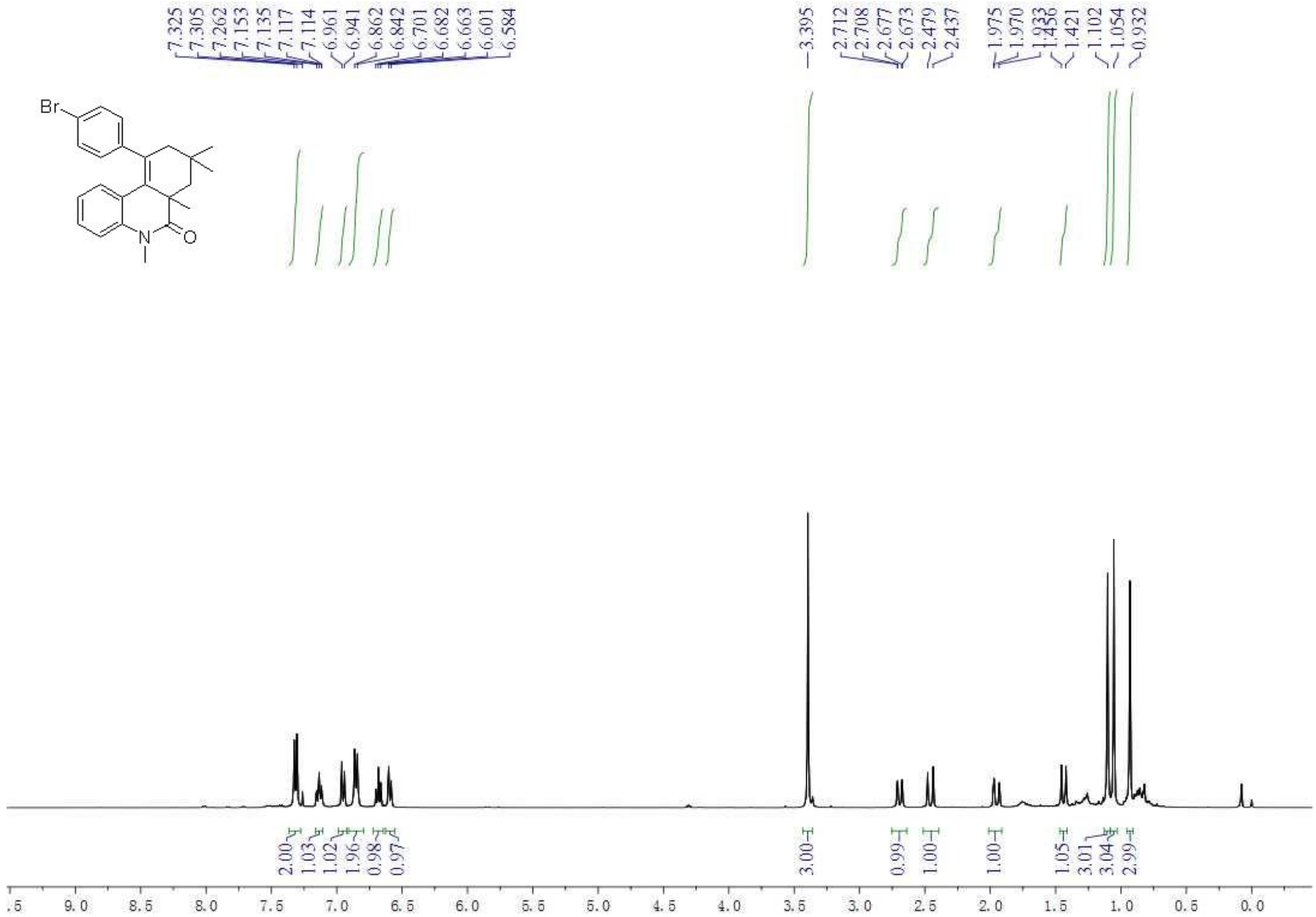

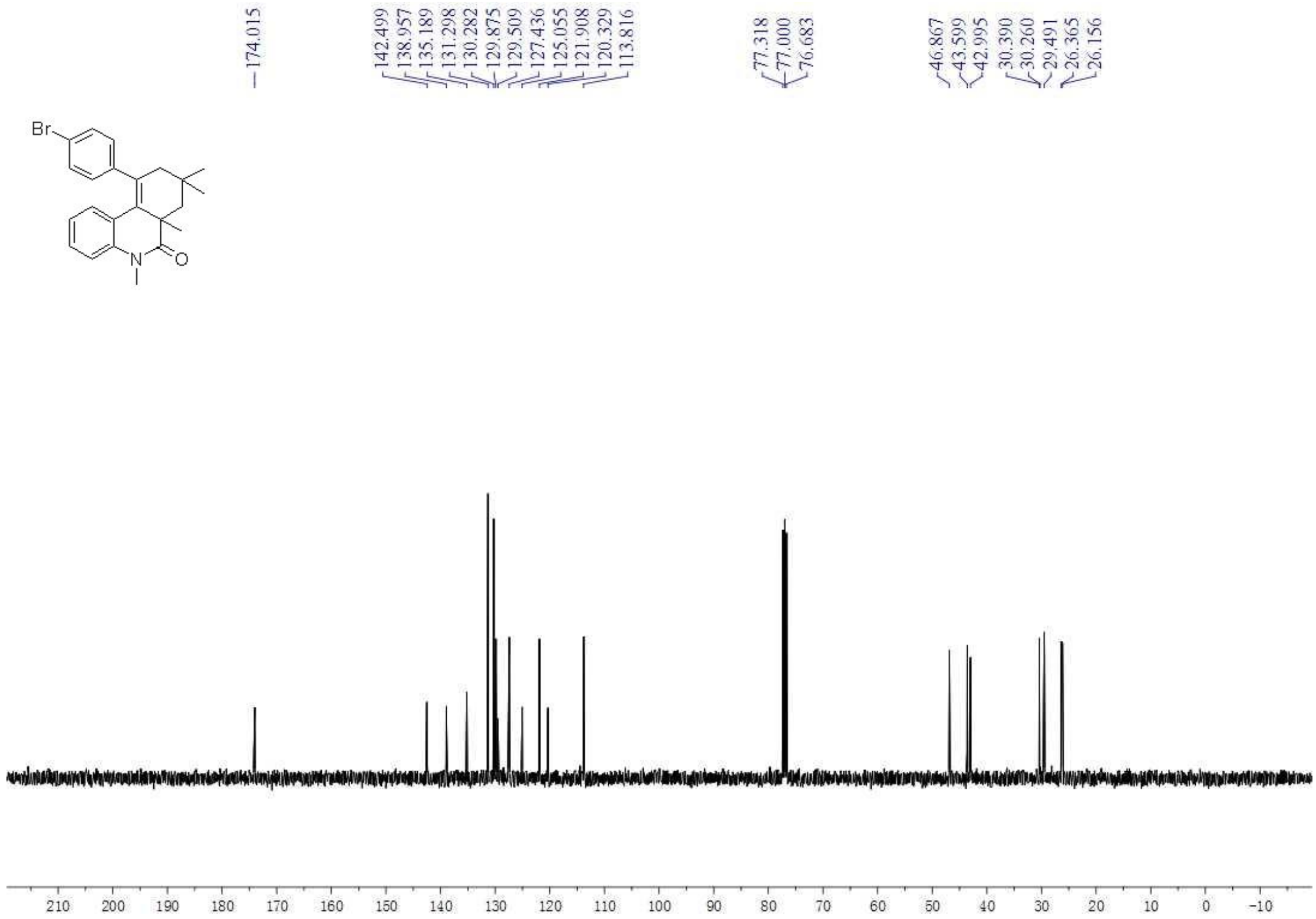

10-(4-Chlorophenyl)-5,6a,8,8-tetramethyl-6a,7,8,9-tetrahydrophenanthridin-6(5 *H*)-one (3ia)

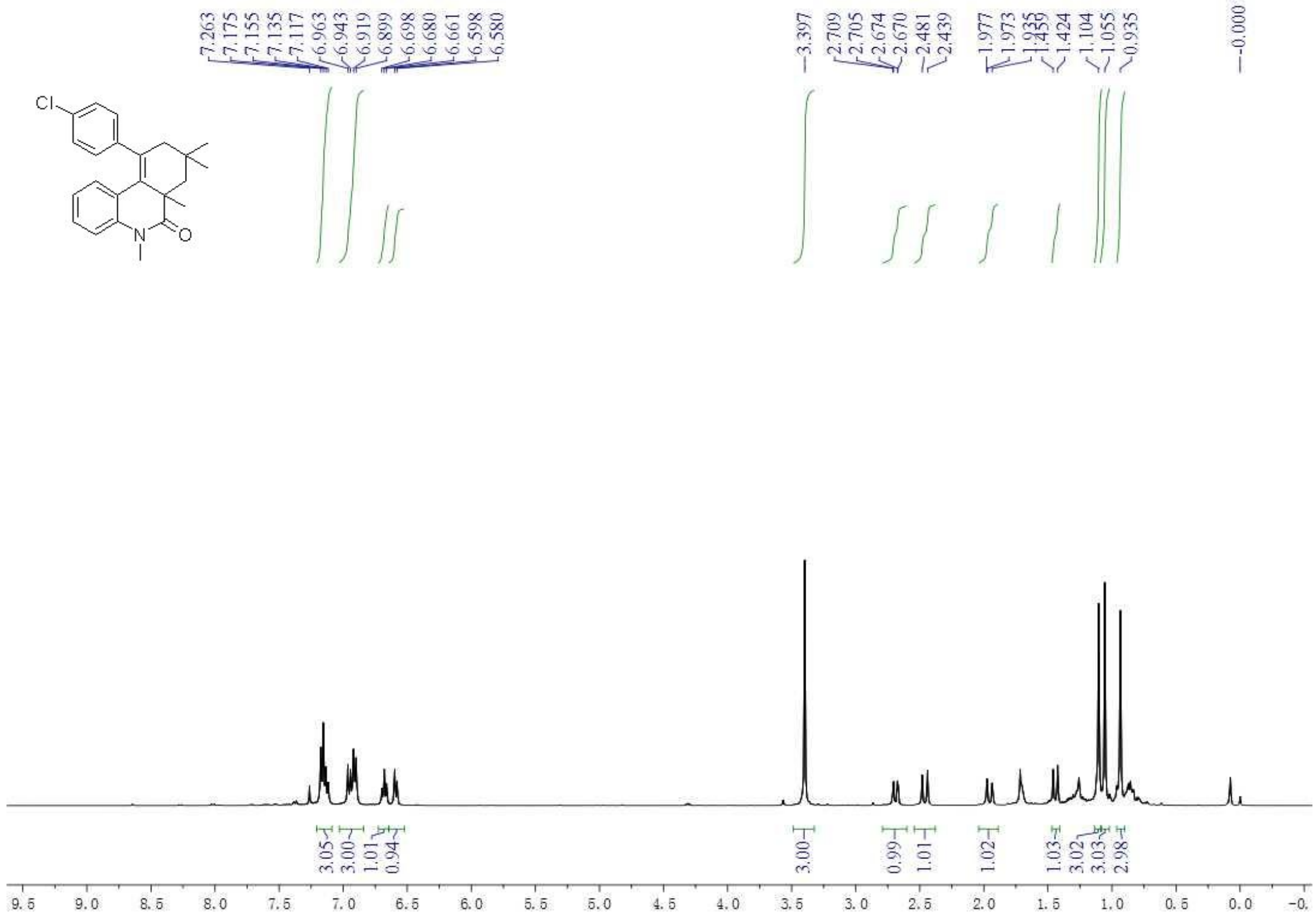

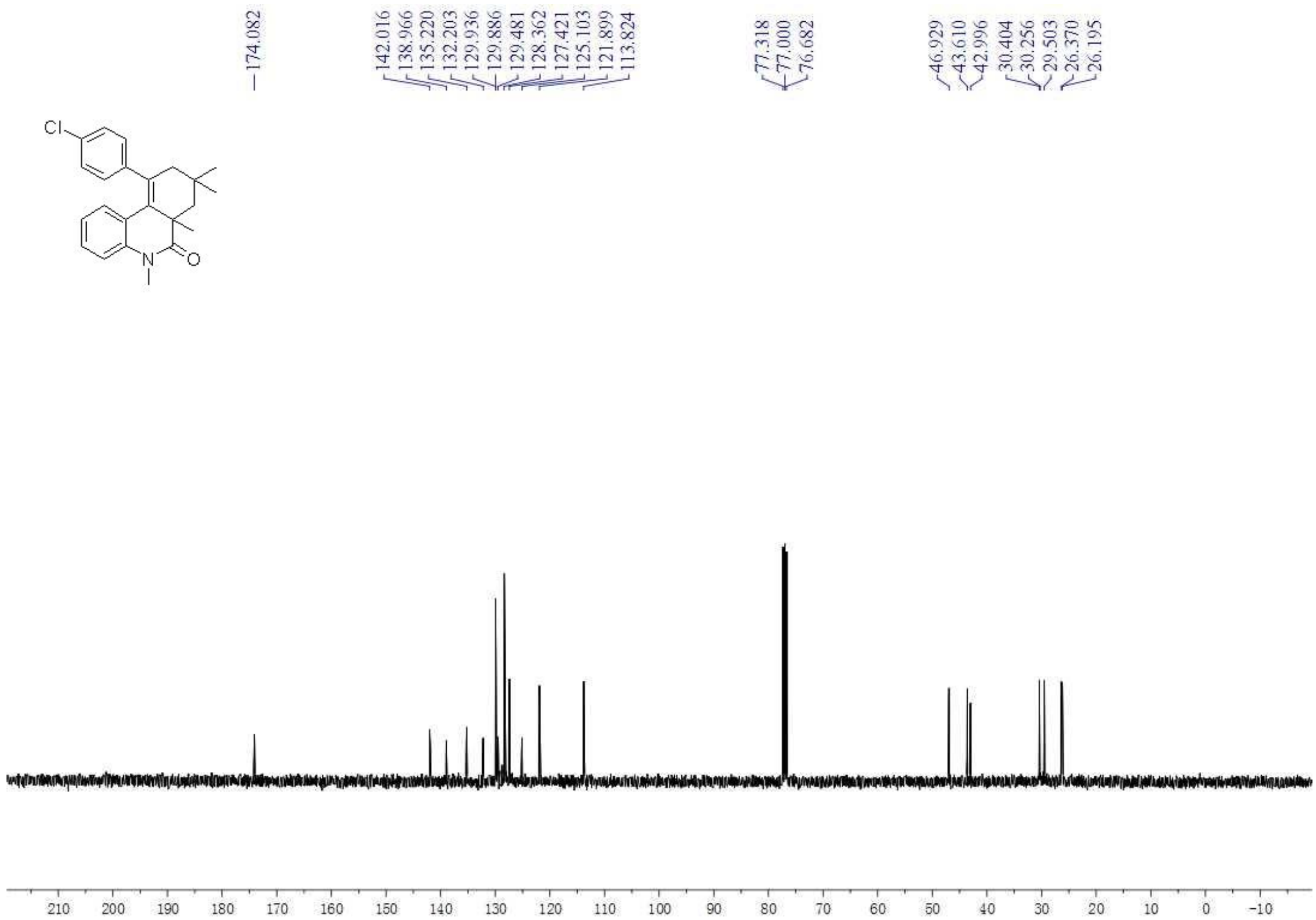

10-(4-Fluorophenyl)-5,6a,8,8-tetramethyl-6a,7,8,9-tetrahydrophenanthridin-6(5H)-one (3ja)

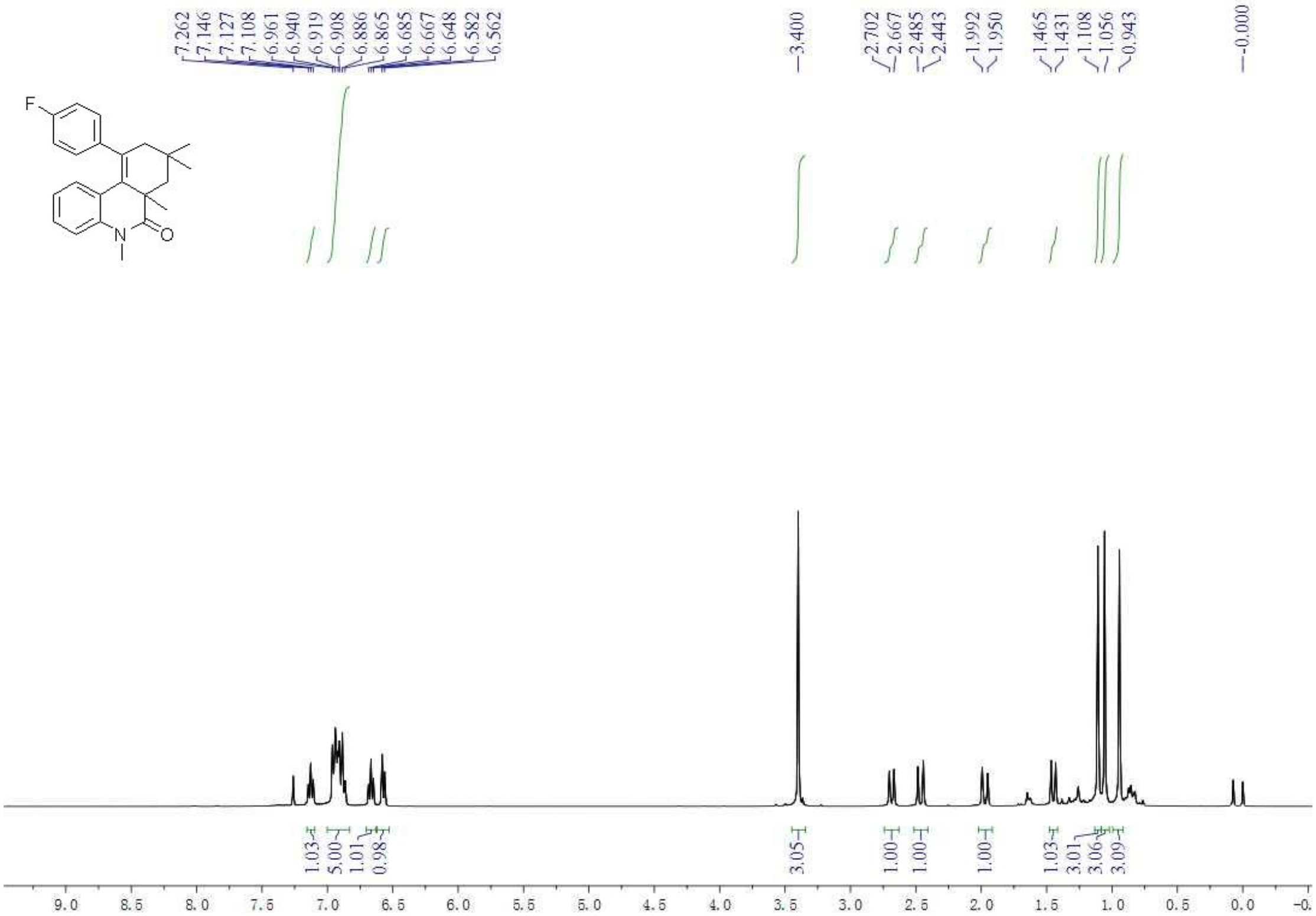

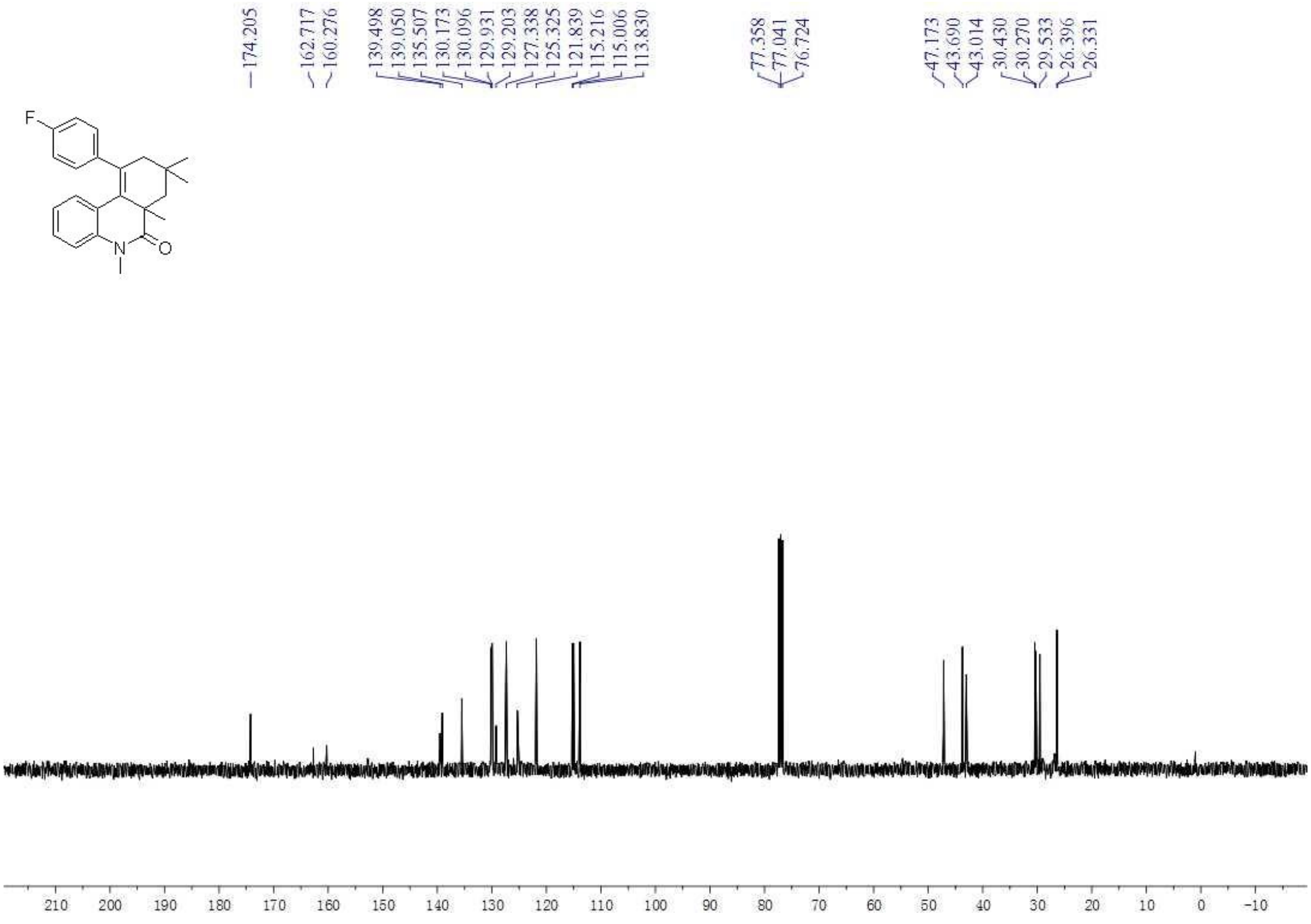

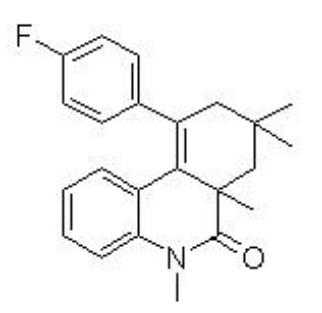
F
N
O

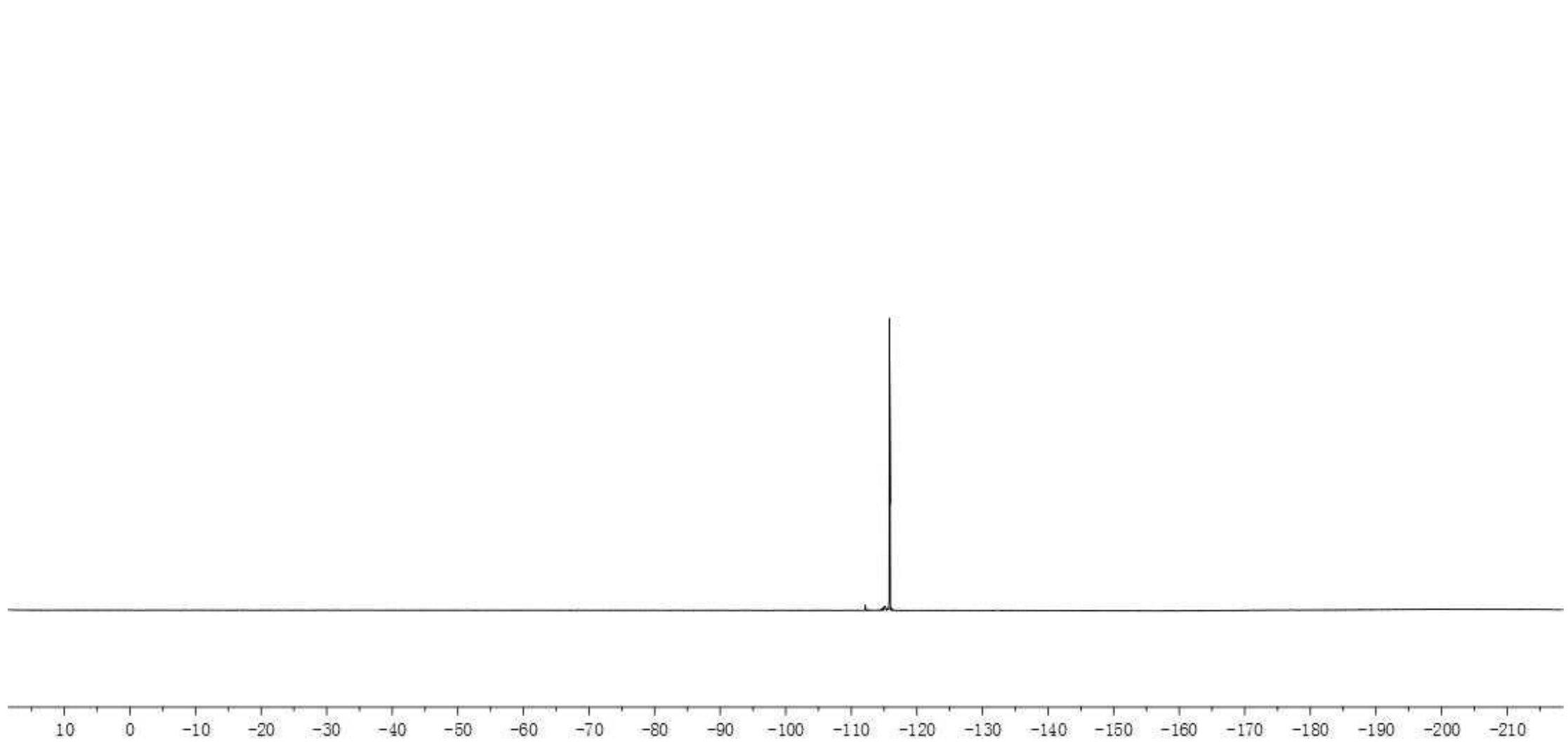
-115.813
10
0
-10
-20
-30
-40
-50
-60
-70
-80
-90
-100
-110
-120
-130
-140
-150
-160
-170
-180
-190
-200
-210

4-(5,6a,8,8-Tetramethyl-6-oxo-5,6,6a,7,8,9-hexahydrophenanthridin-10-yl)benzo nitrile (3ka)

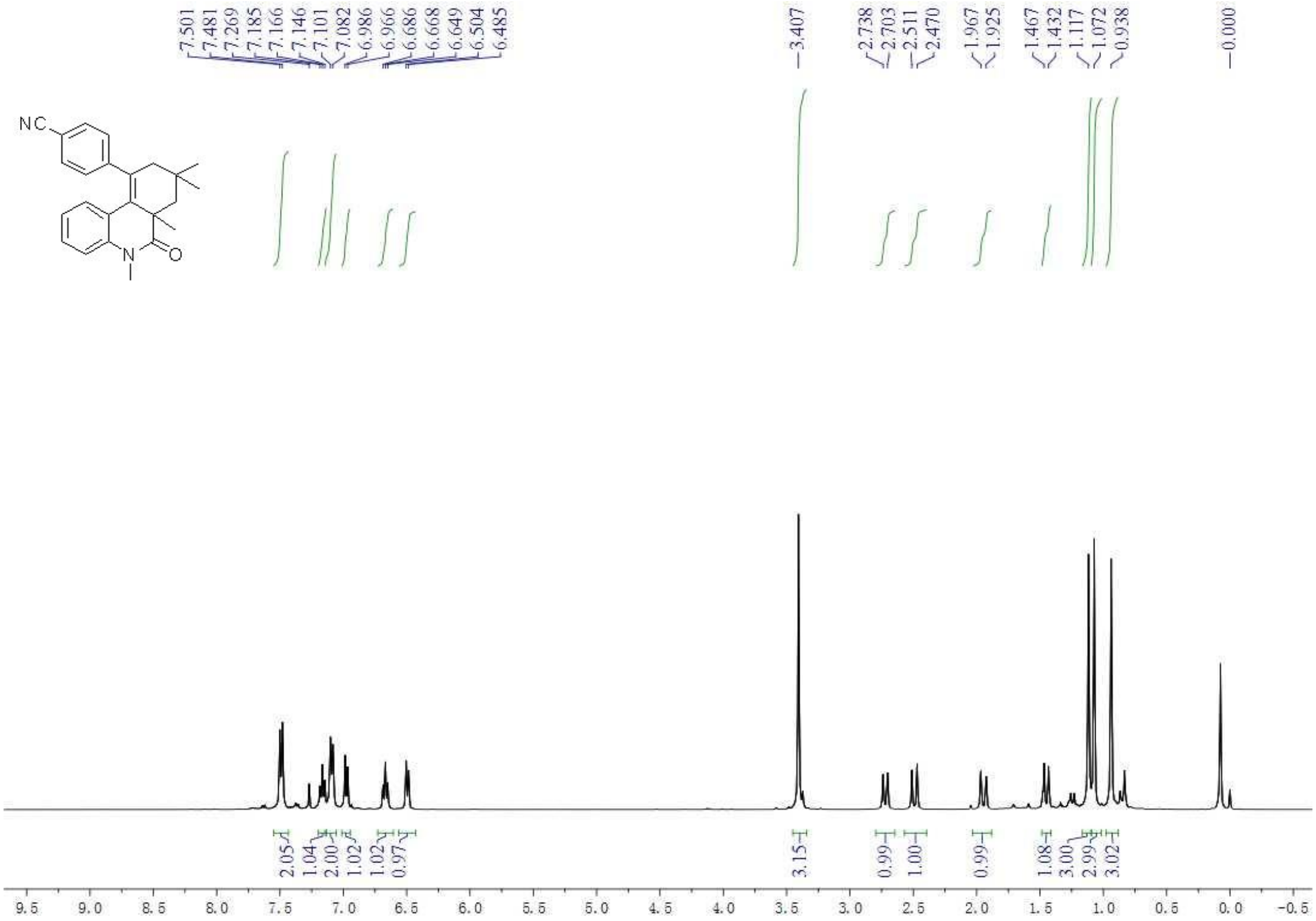

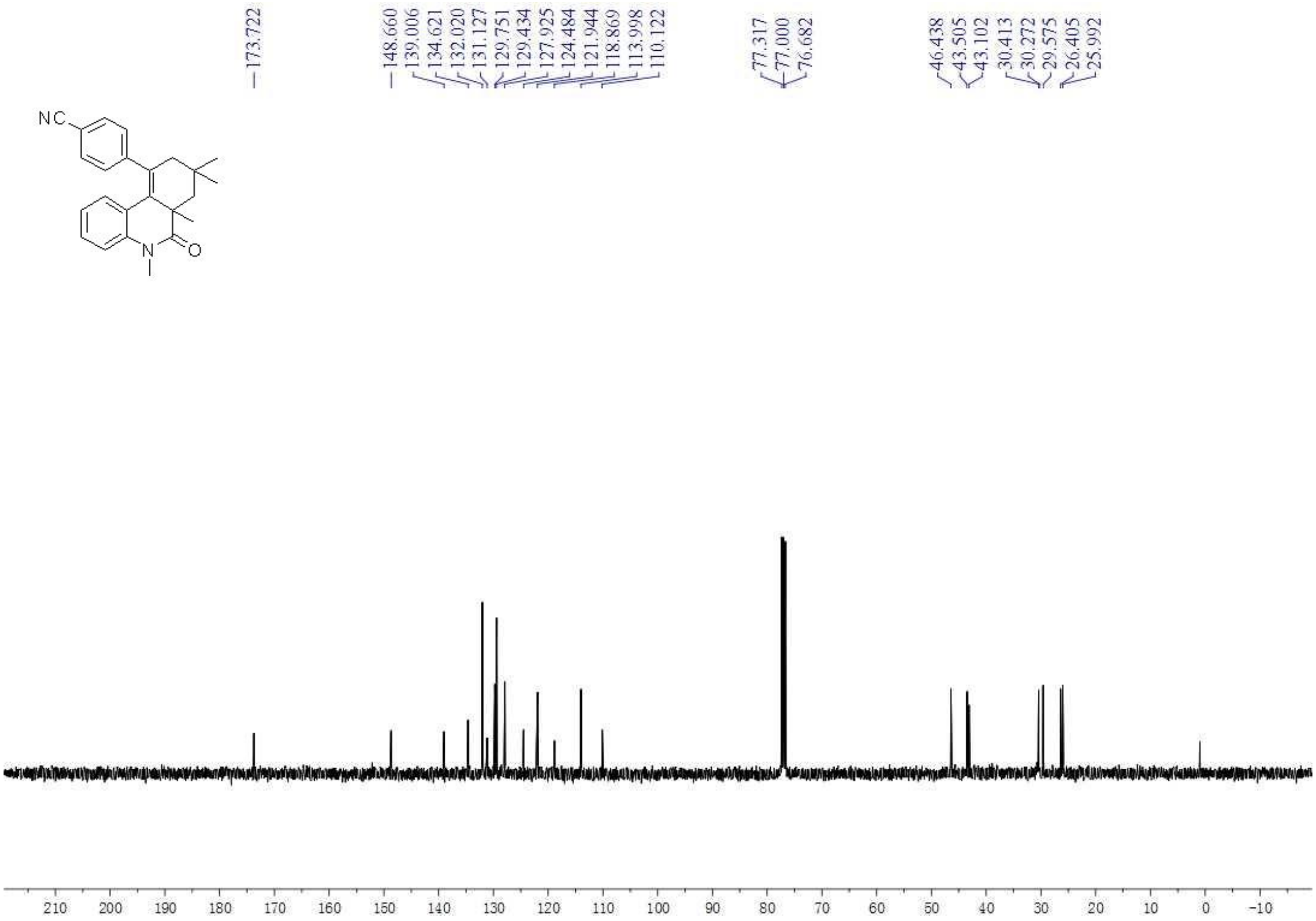

5,6a,8,8-Tetramethyl-10-(*m*-tolyl)-6a,7,8,9-tetrahydrophenanthridin-6(5*H*)-one (3la)

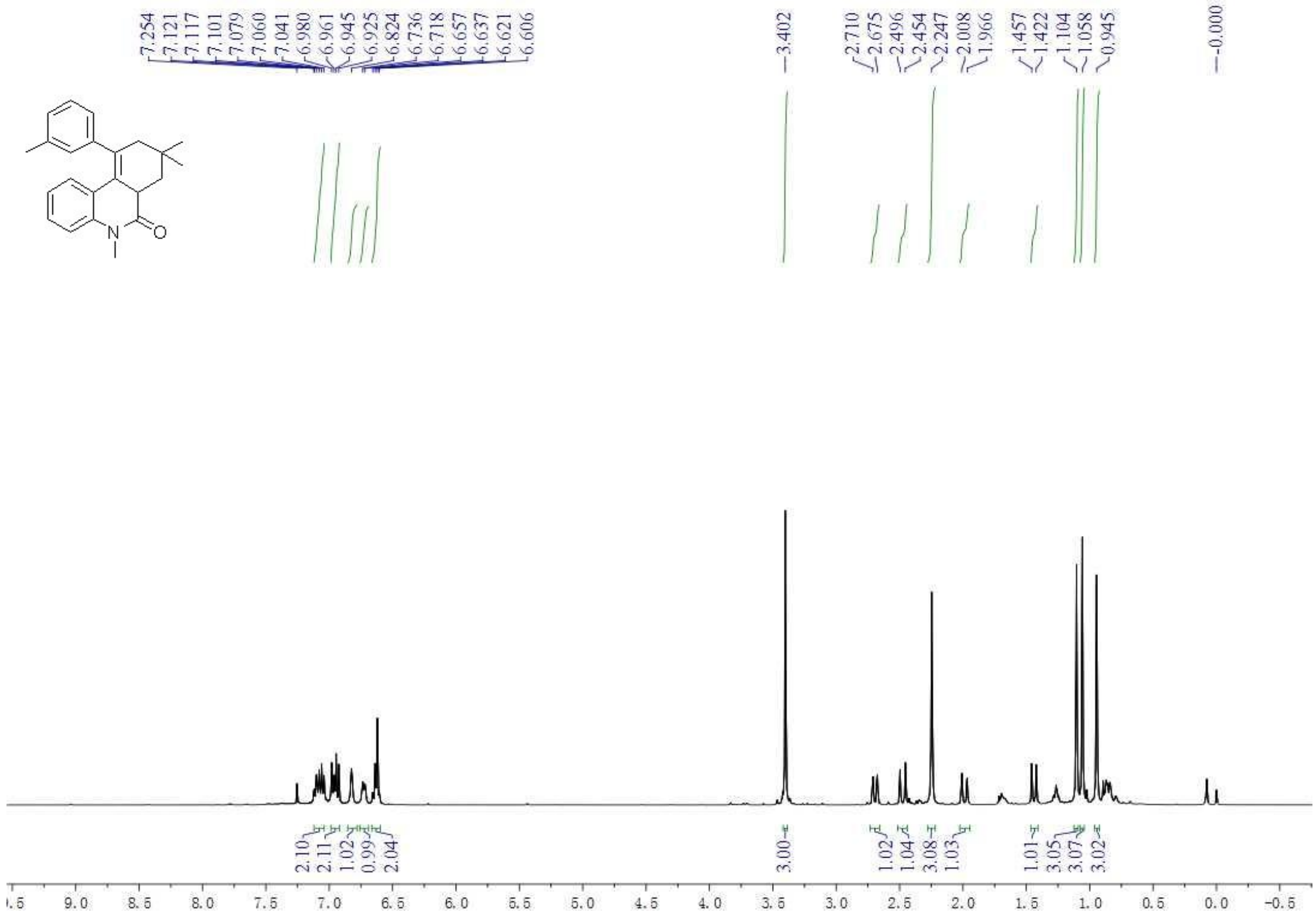

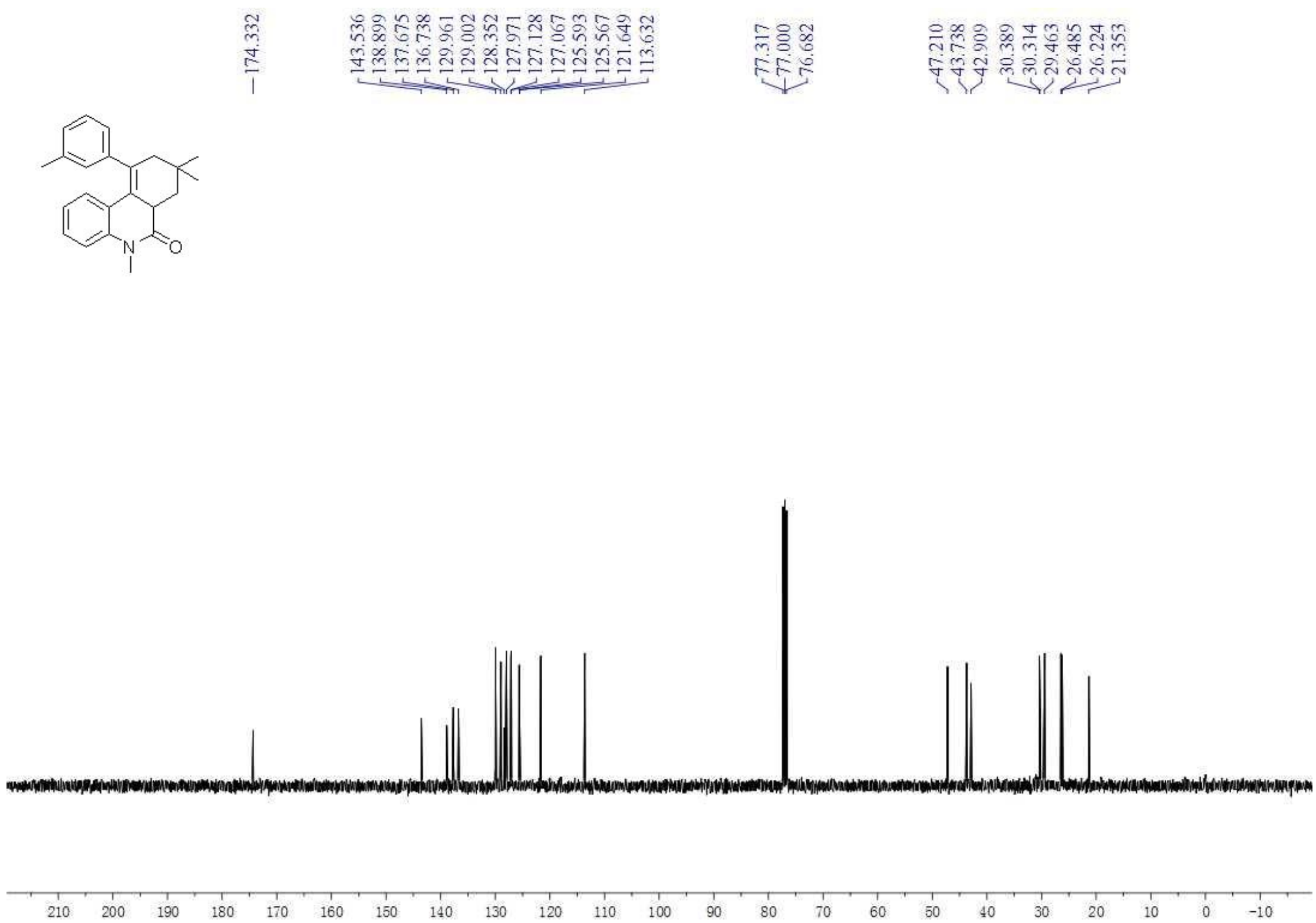

10-(3-Bromophenyl)-5,6a,8,8-tetramethyl-6a,7,8,9-tetrahydrophenanthridin-6(5H)-one (3ma)

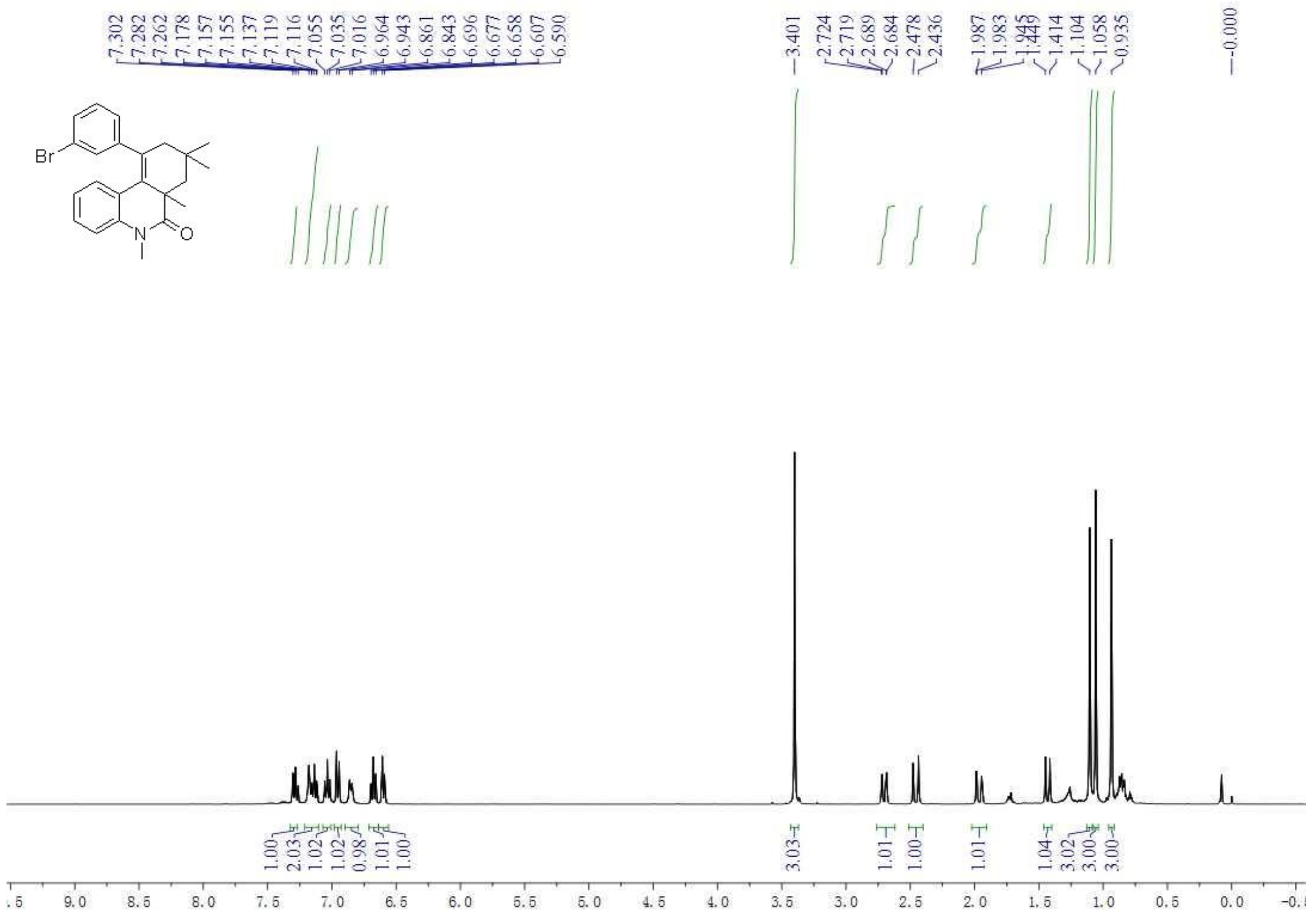

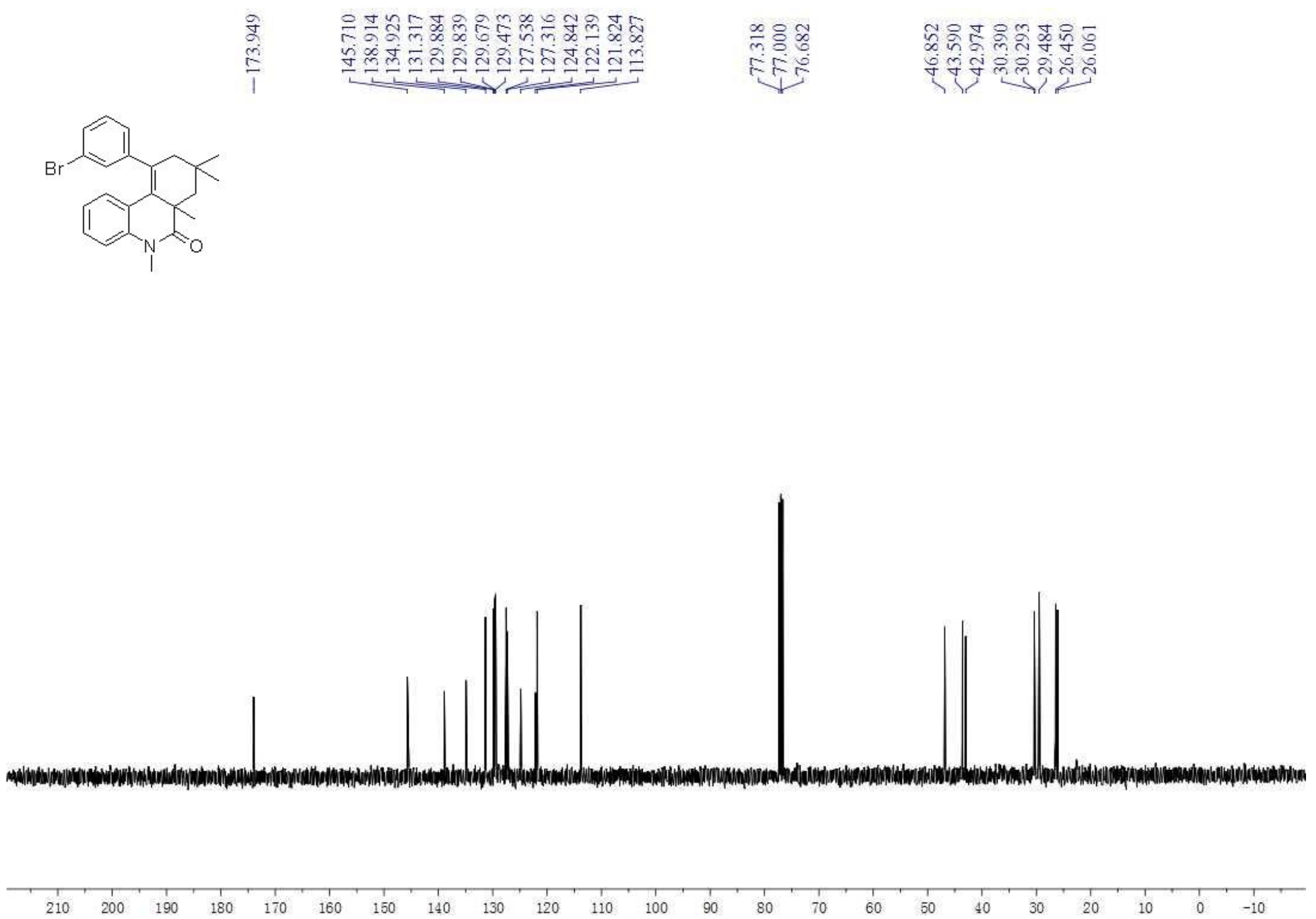

5,6a,8,8-Tetramethyl-10-(pyridin-3-yl)-6a,7,8,9-tetrahydrophenanthridin-6(5*H*)-one (3na)

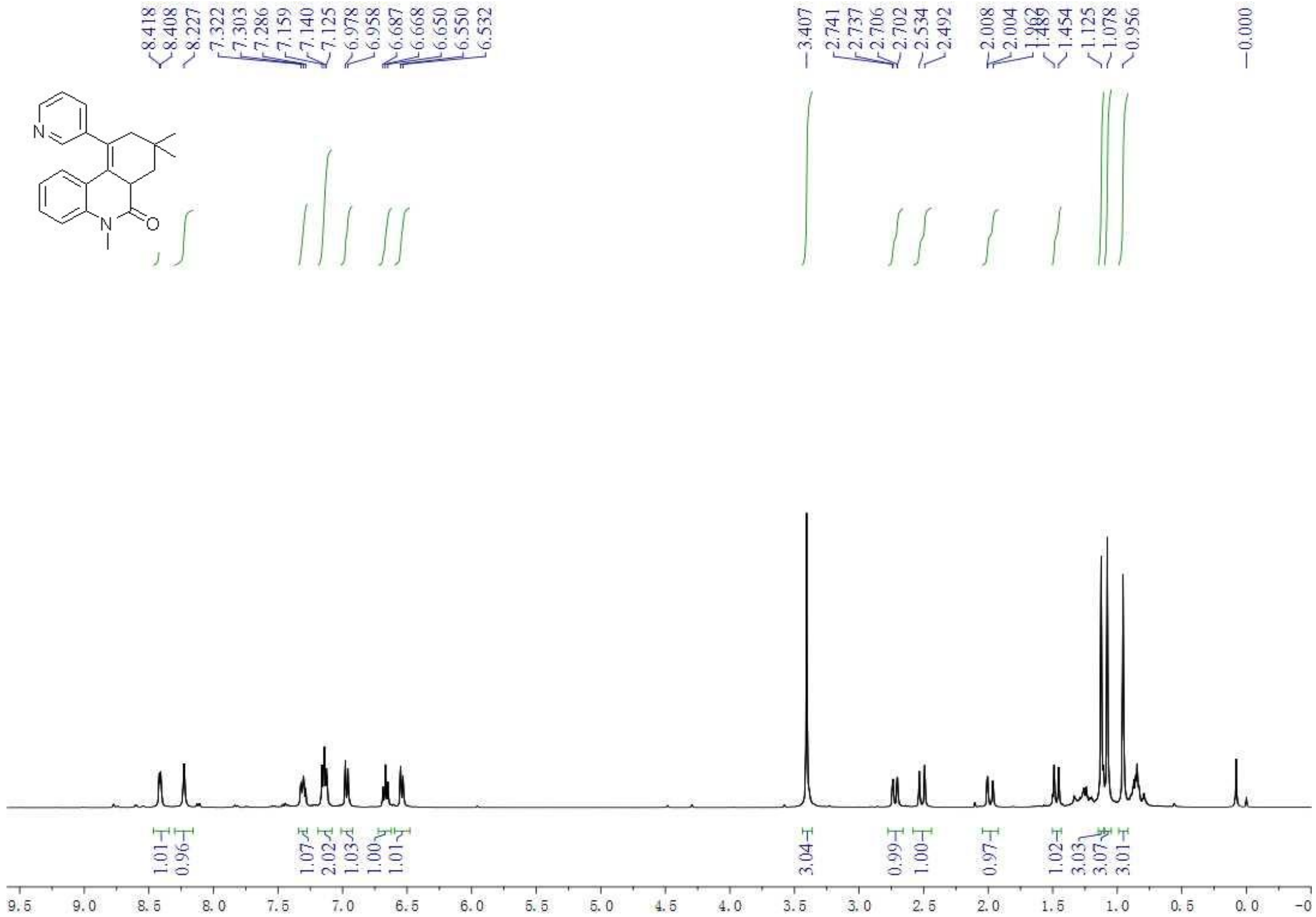

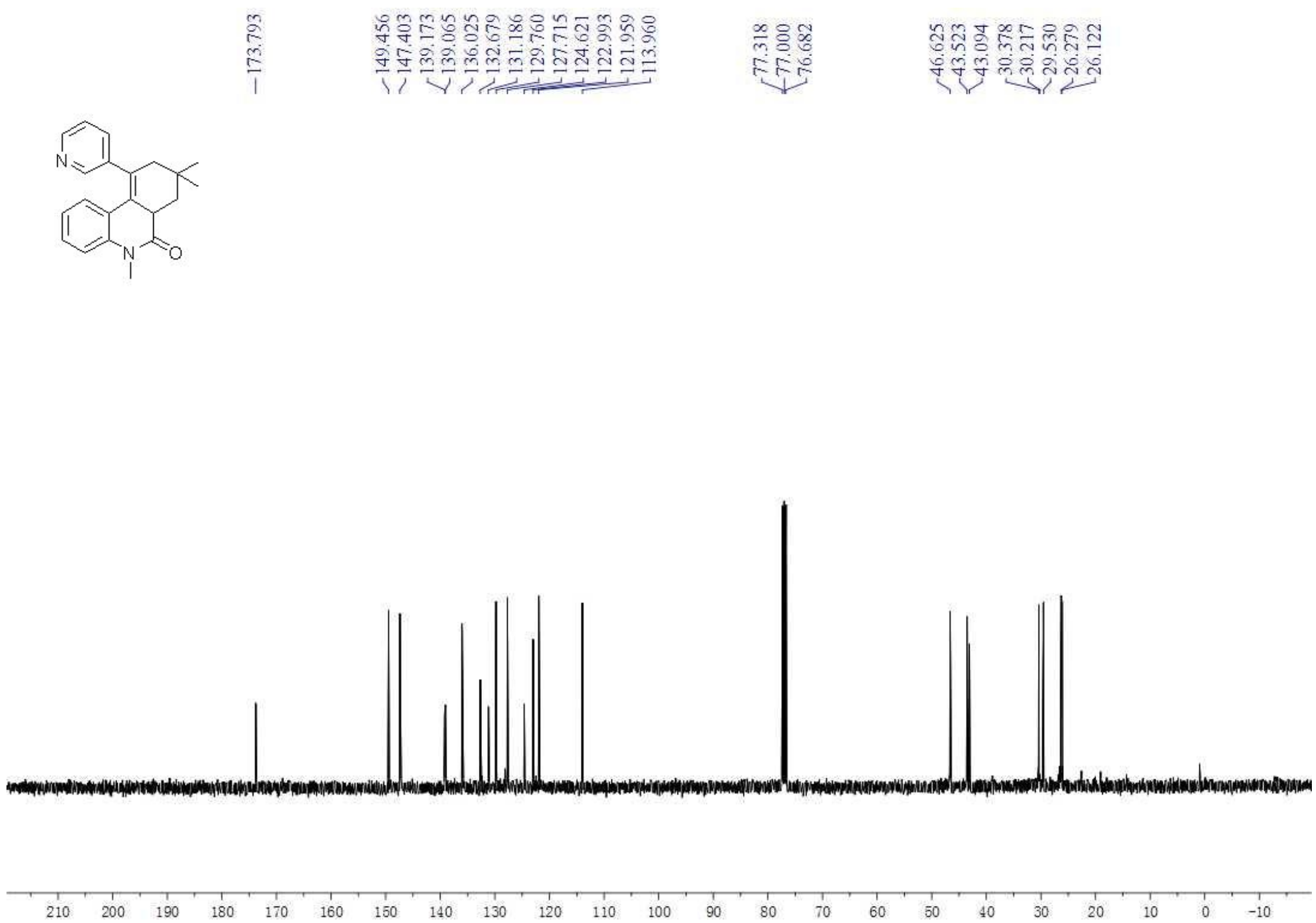

10-Hexyl-5,6a,8,8-tetramethyl-6a,7,8,9-tetrahydrophenanthridin-6(5*H*)-one (3oa)

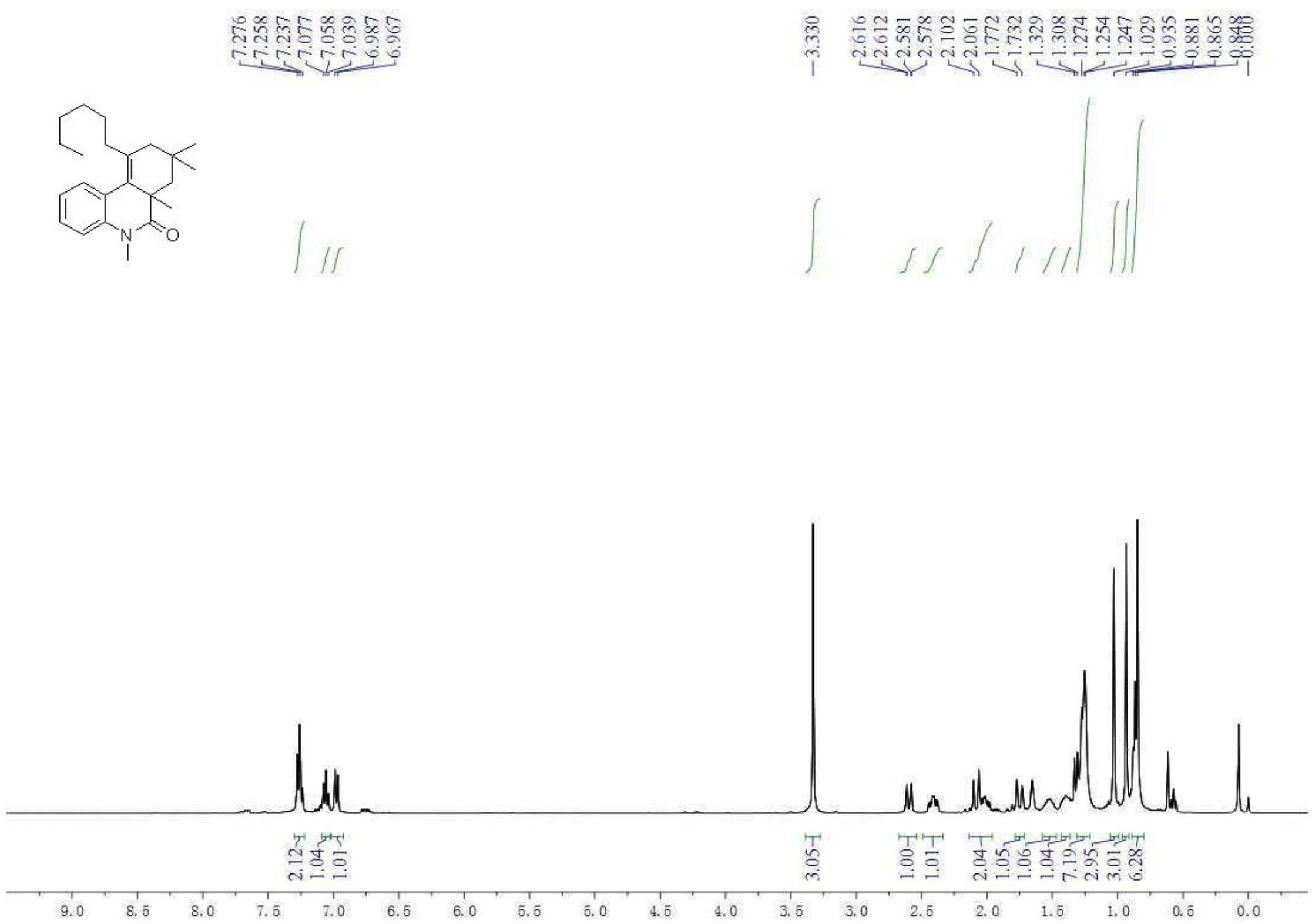

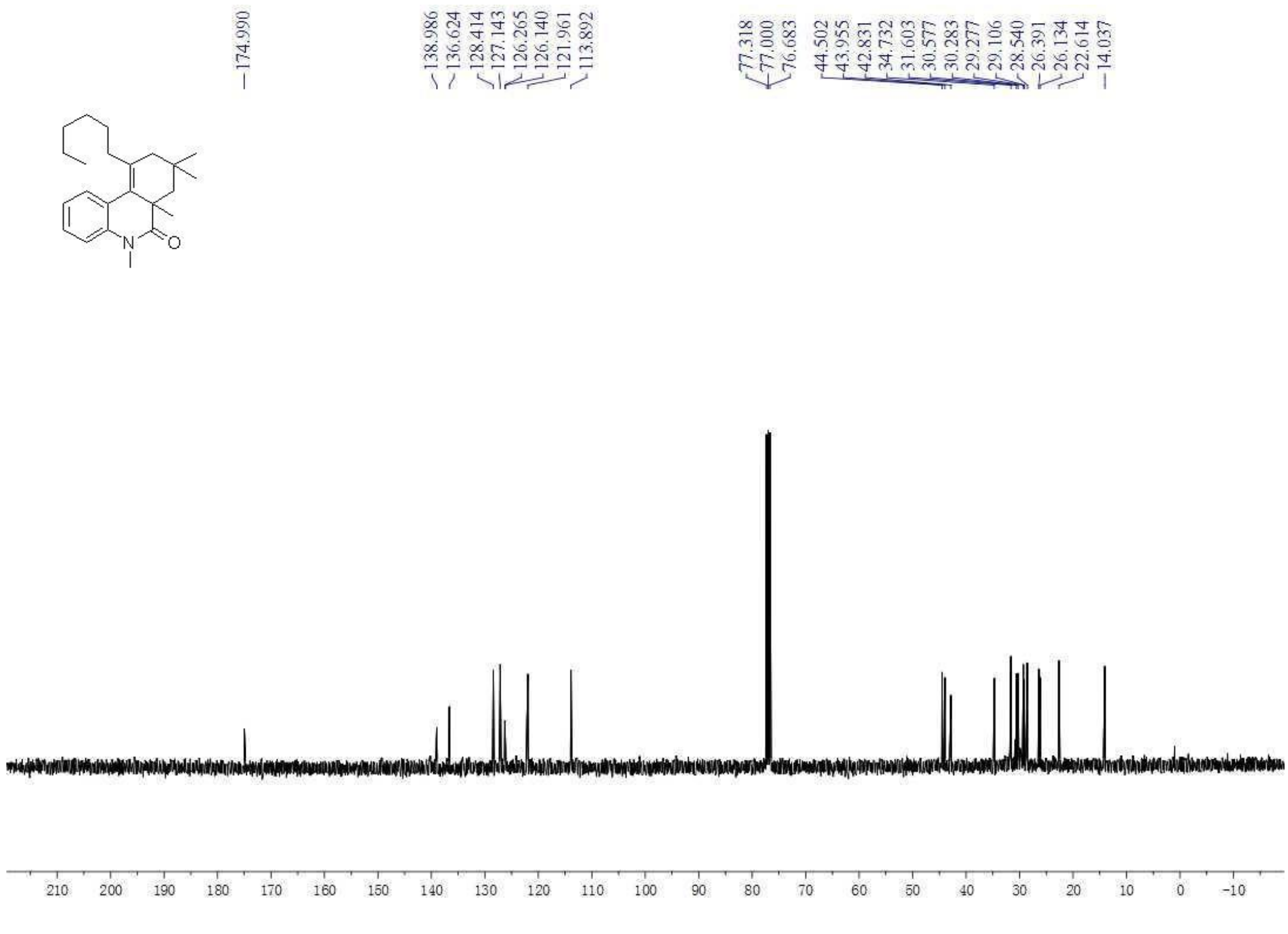

2,5,6a,8,8-Pentamethyl-10-phenyl-6a,7,8,9-tetrahydrophenanthridin-6(5*H*)-one (3pa)

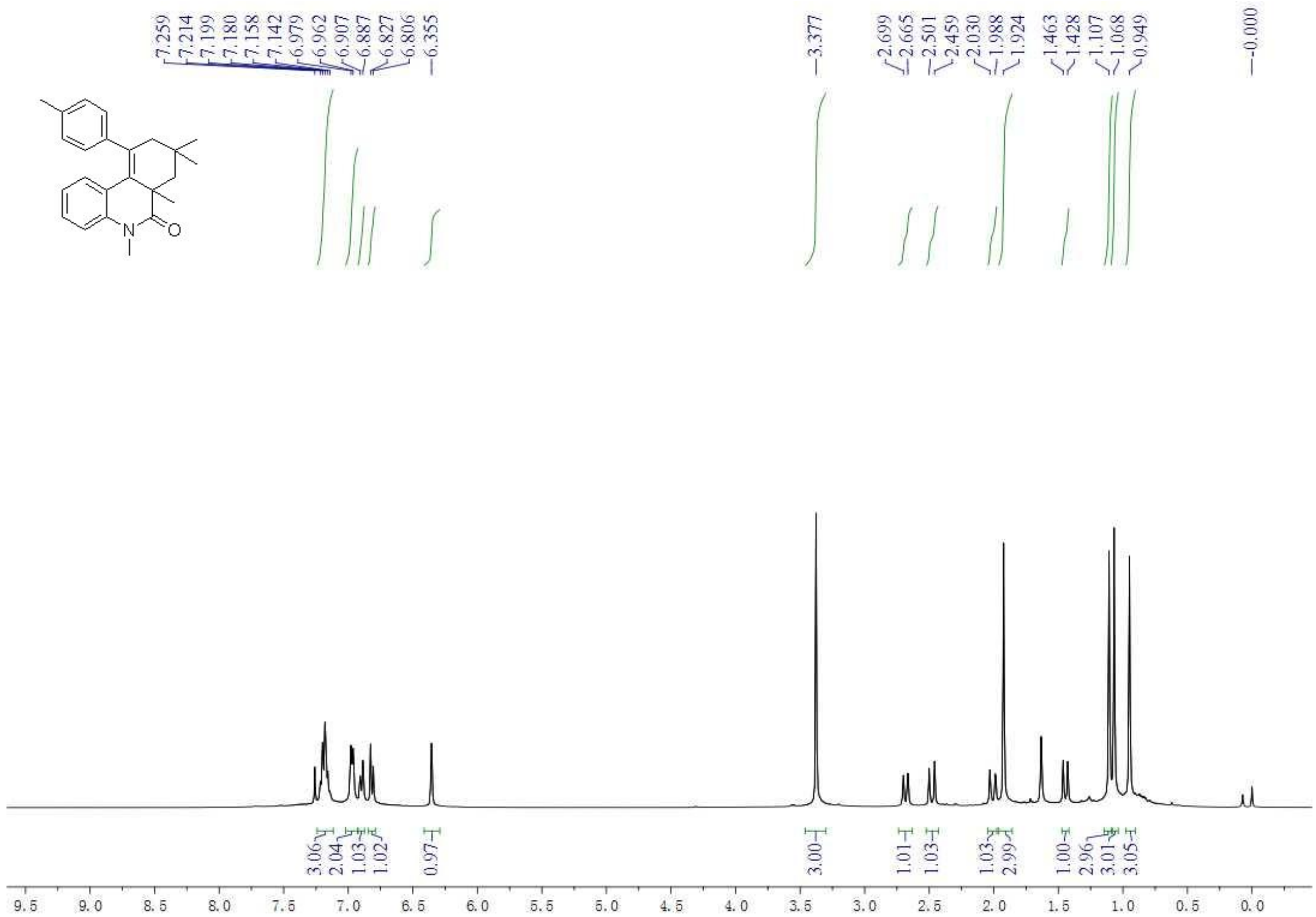

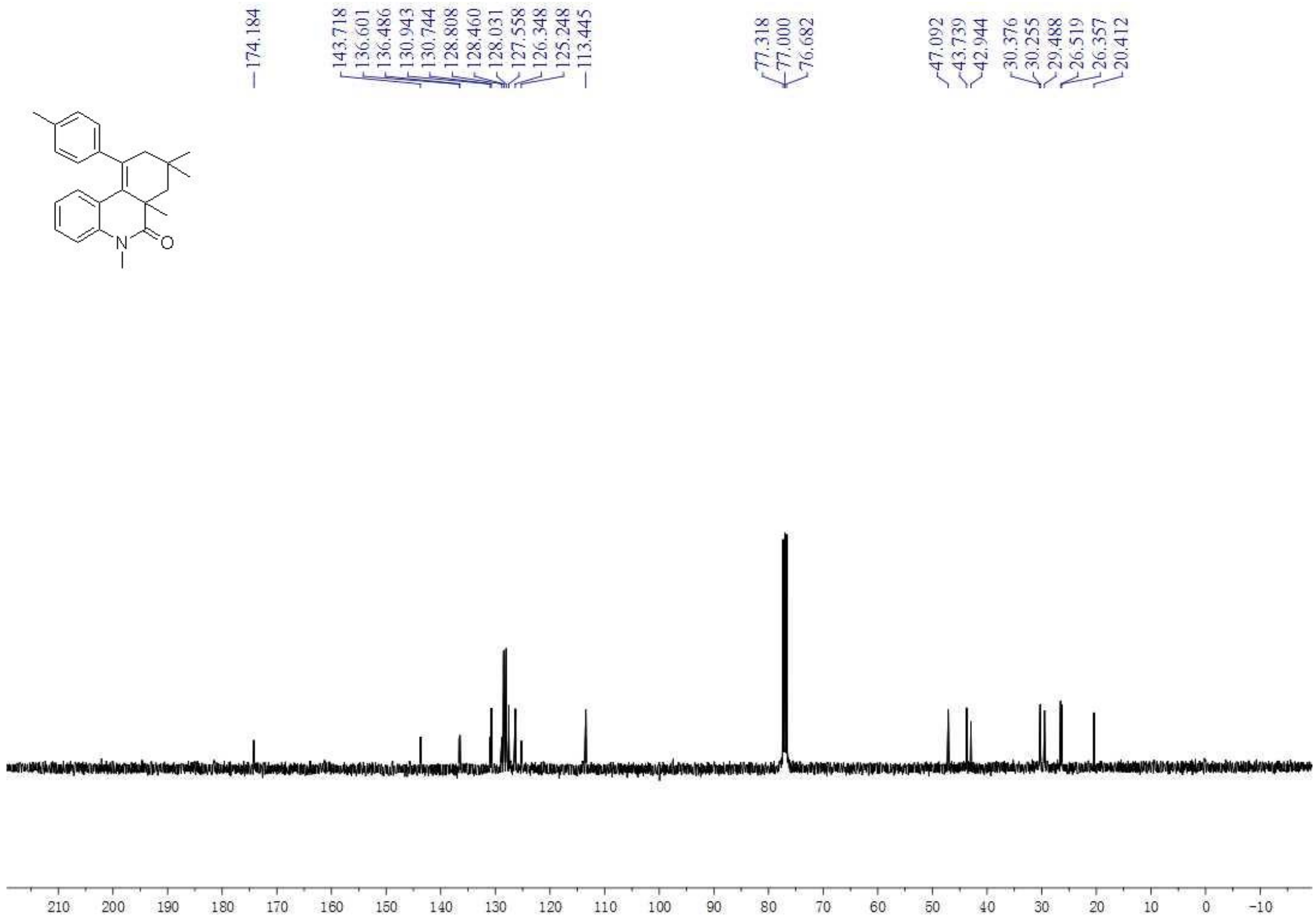

2-Chloro-5,6a,8,8-tetramethyl-10-phenyl-6a,7,8,9-tetrahydrophenanthridin-6(5*H*)-one (3qa)

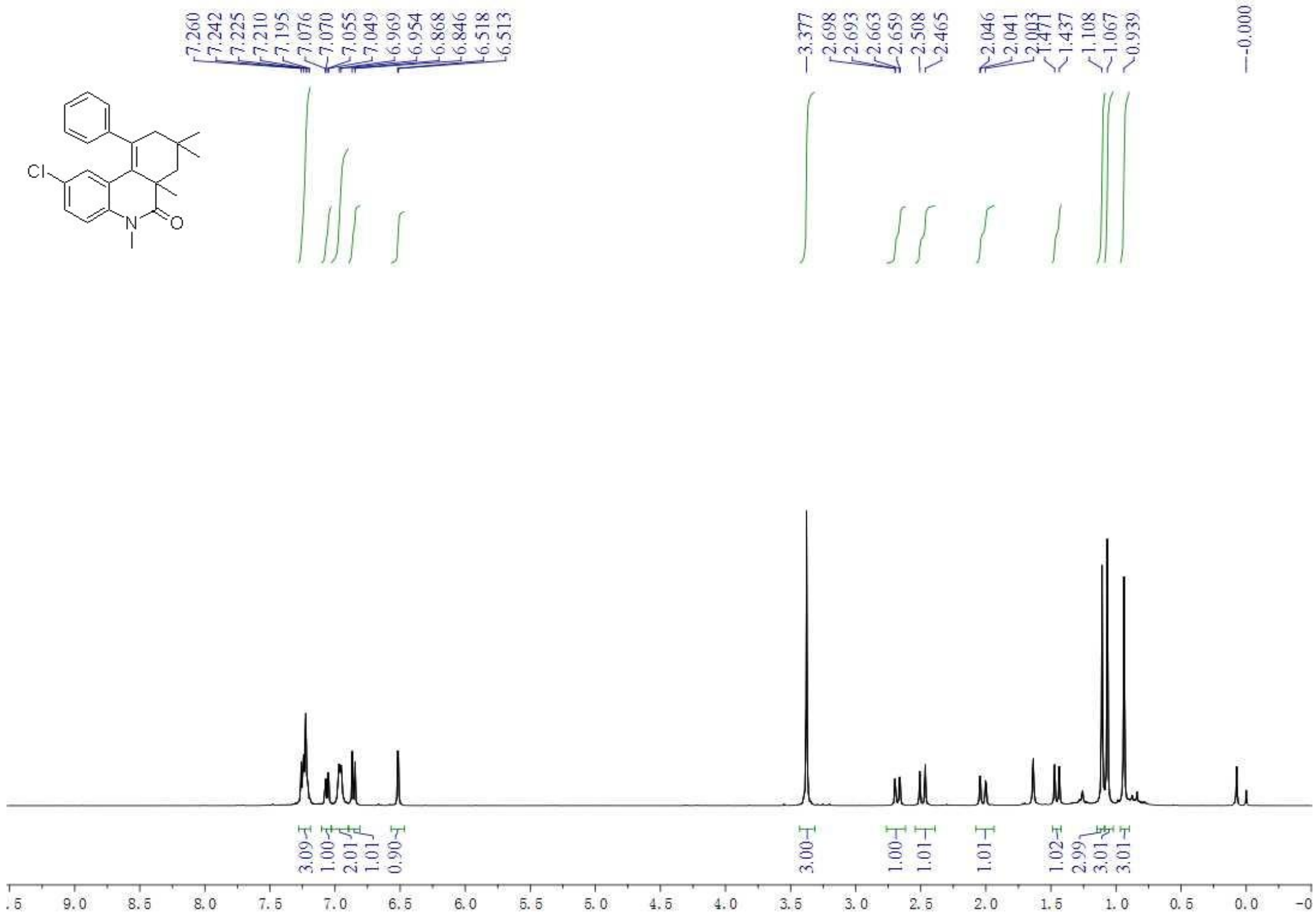

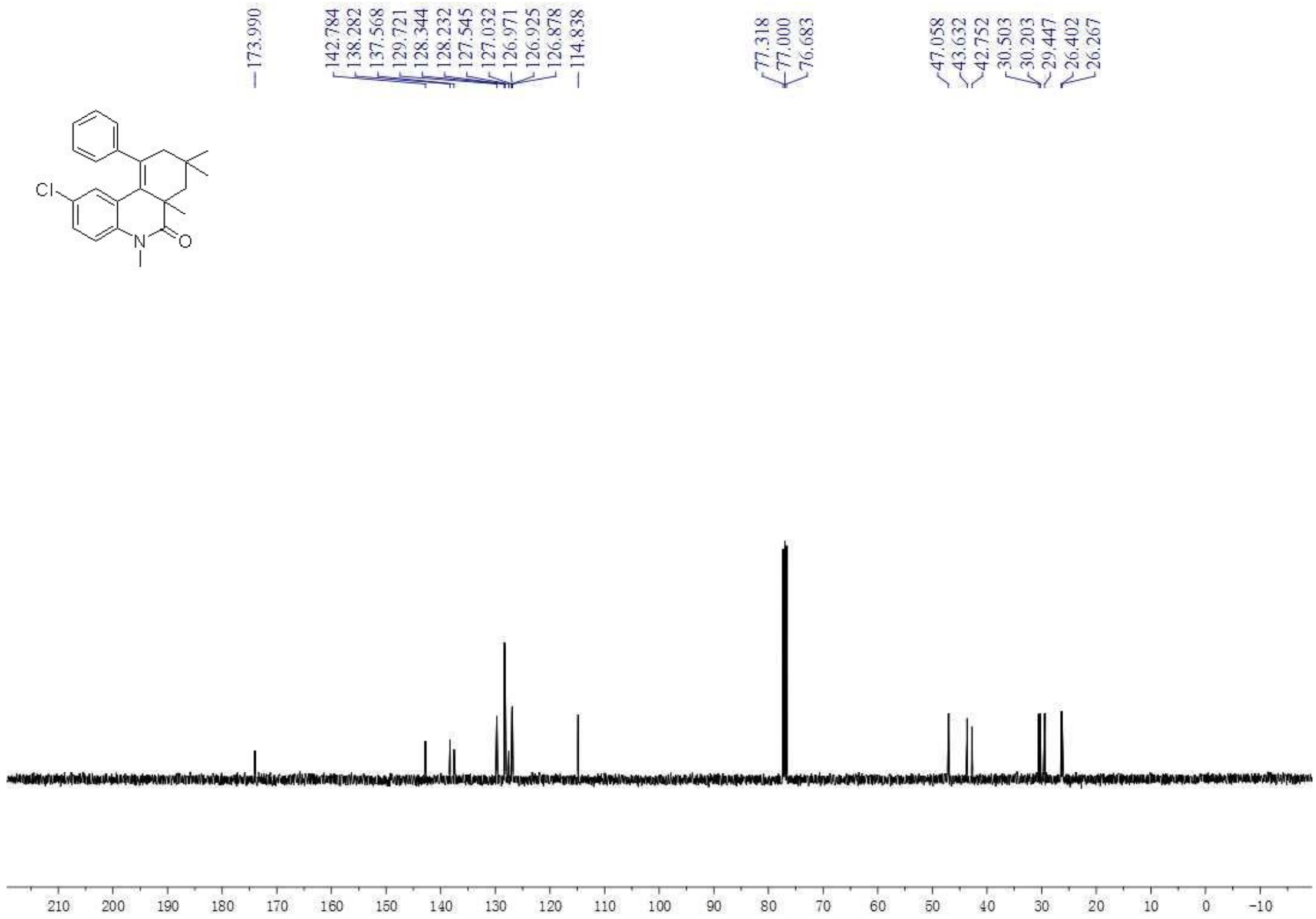

3,5,6a,8,8-Pentamethyl-10-phenyl-6a,7,8,9-tetrahydrophenanthridin-6(5*H*)-one (3ra)

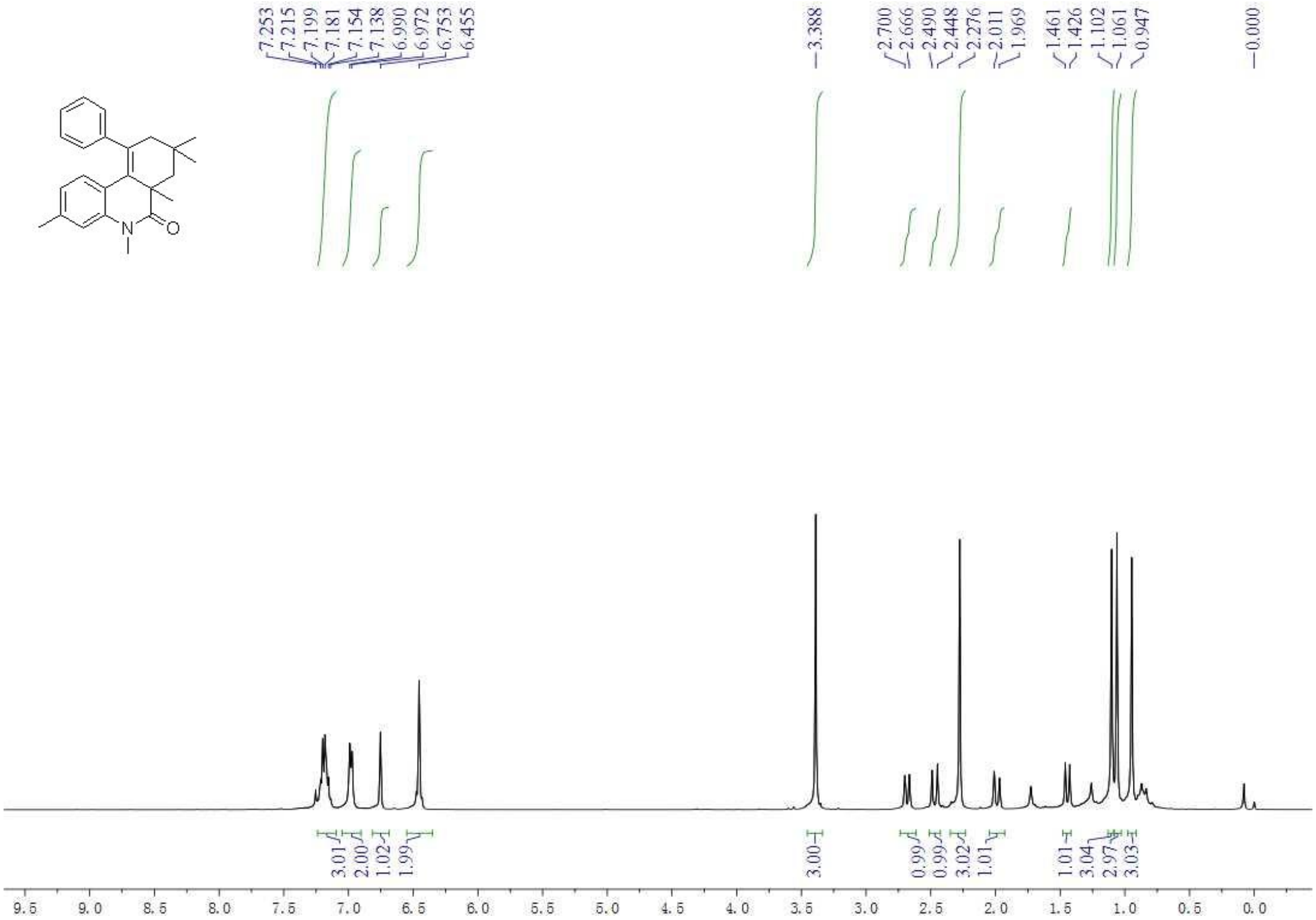

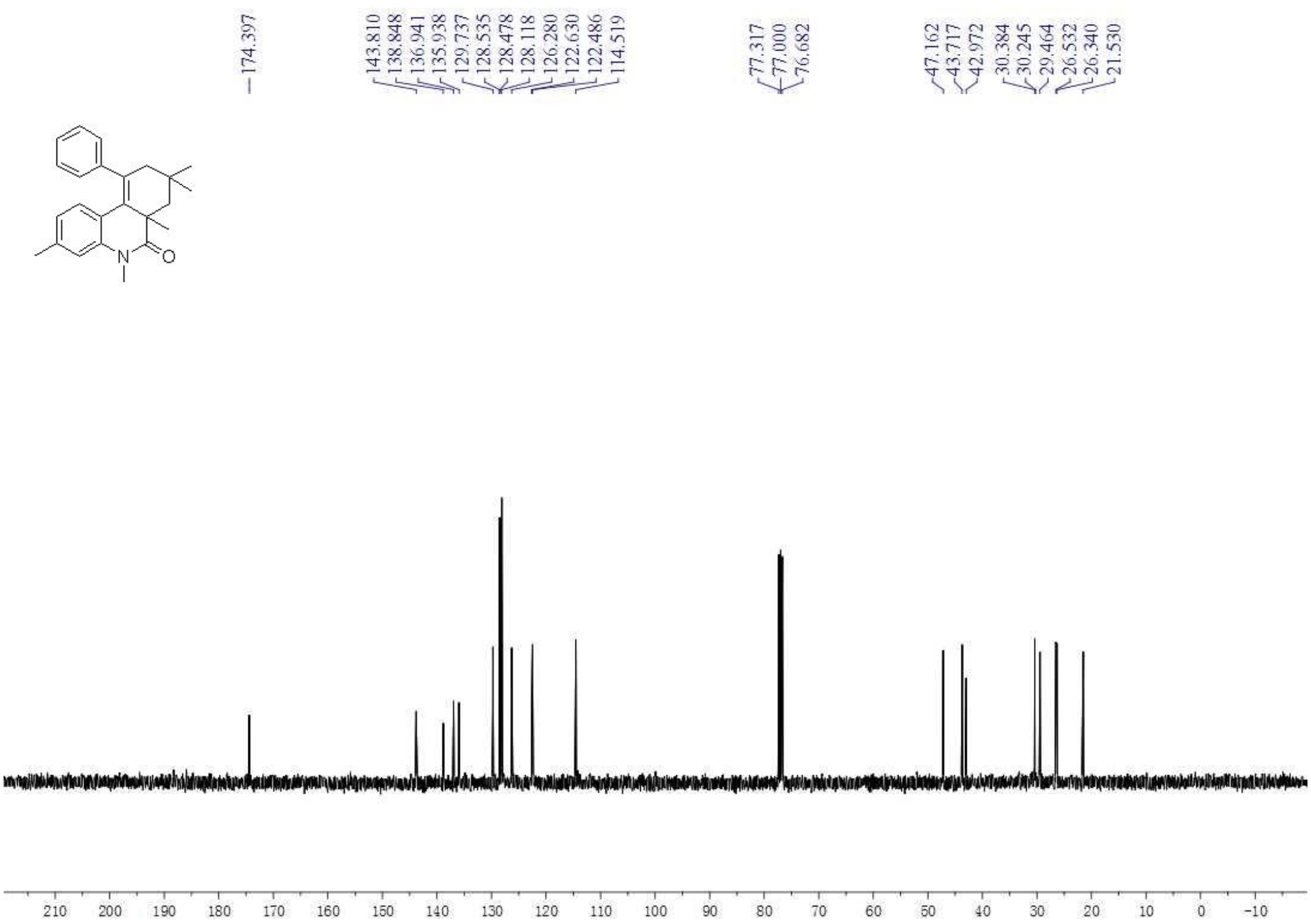

5-Methyl-6a-neopentyl-11-phenyl-5,6a-dihydro-6*H*-indeno[1,2-*c*]quinolin-6-one (3sa)

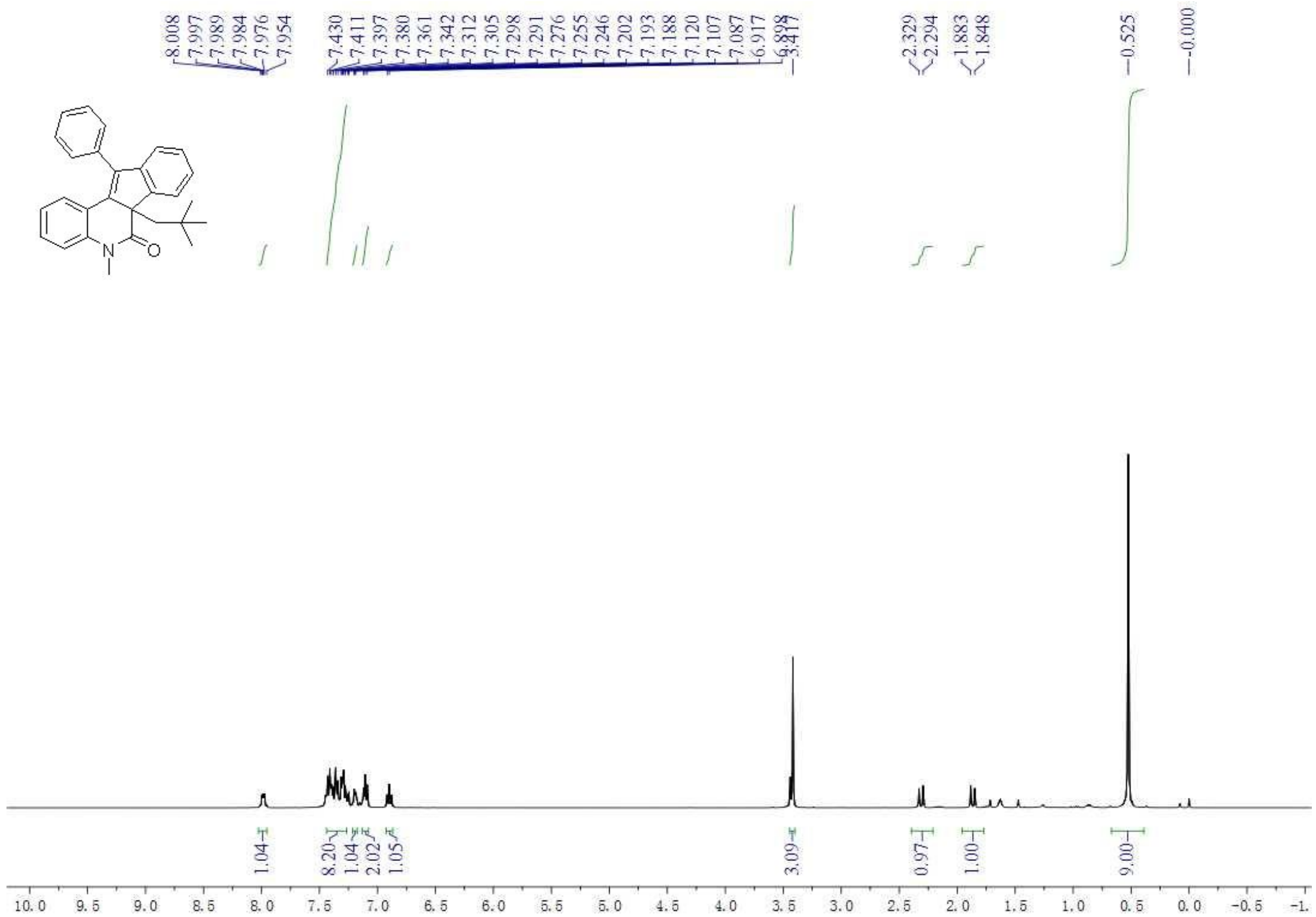

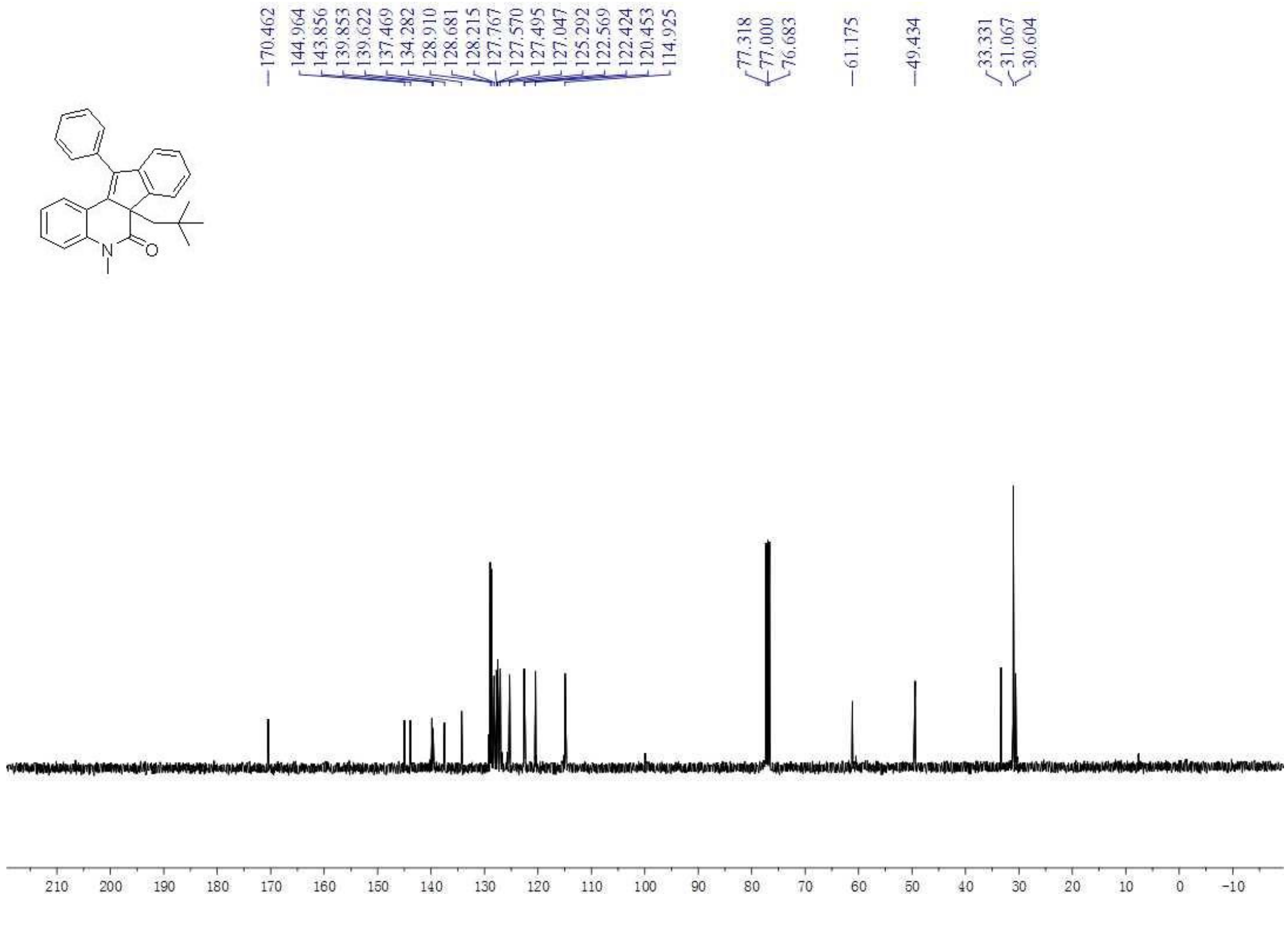

3-(8-Ethyl-5,6a,9-trimethyl-6-oxo-10-phenyl-5,6,6a,7,8,9-hexahydrophenanthridin-8-yl)propanenitrile (3ab)

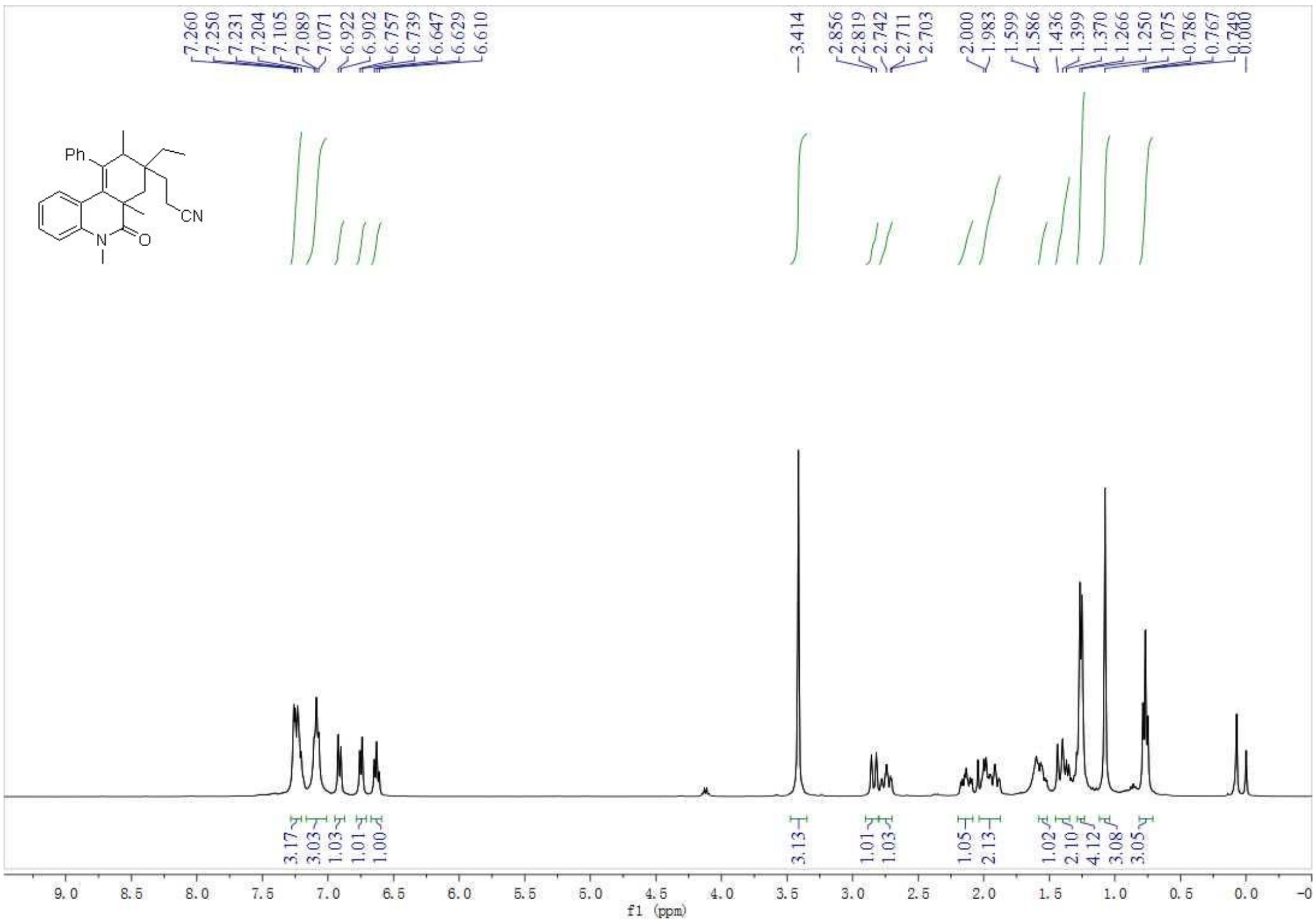

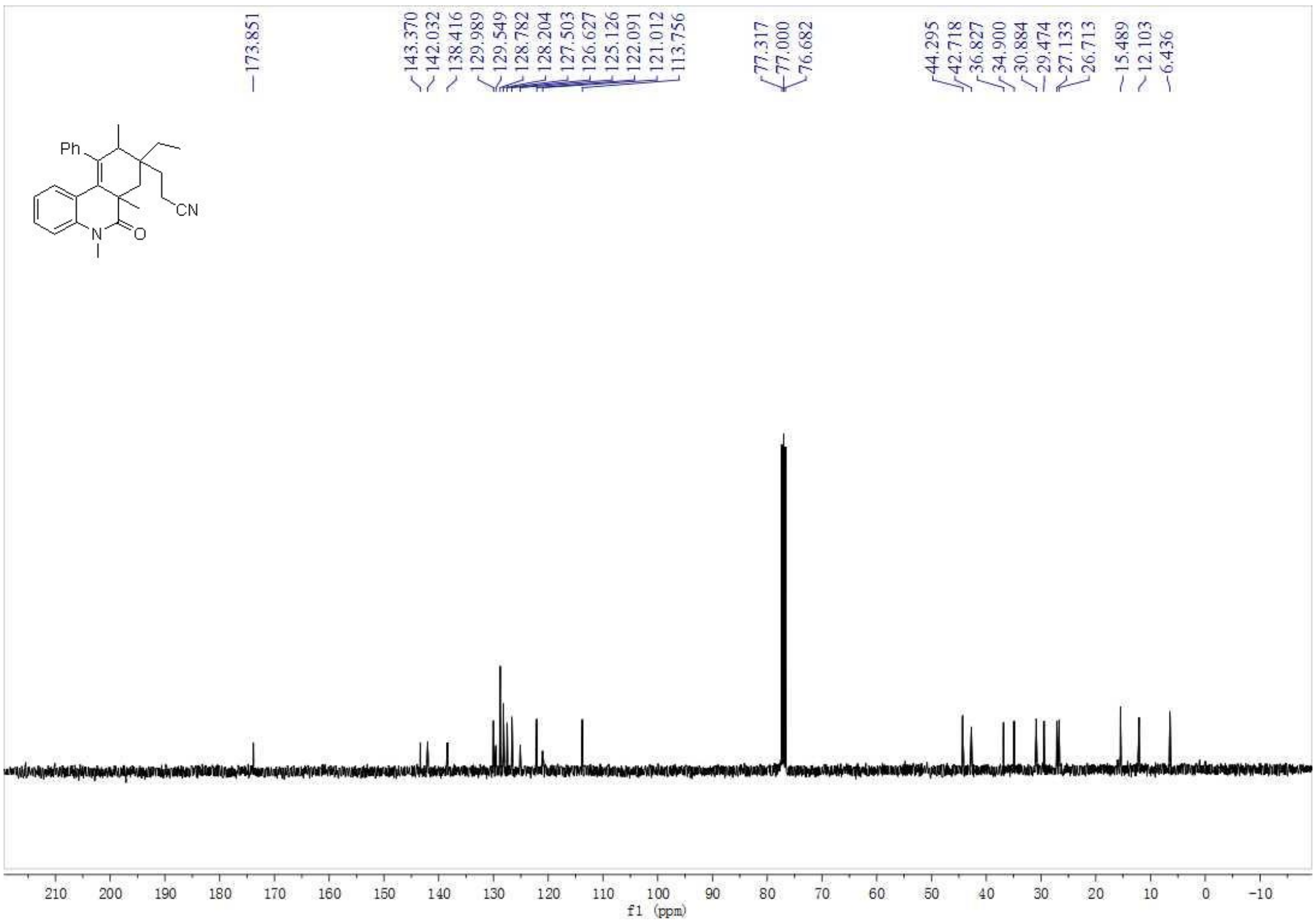

8,8-Diethyl-5,6a-dimethyl-10-phenyl-9-vinyl-6a,7,8,9-tetrahydrophenanthridin-6(5*H*)-one (3ac)

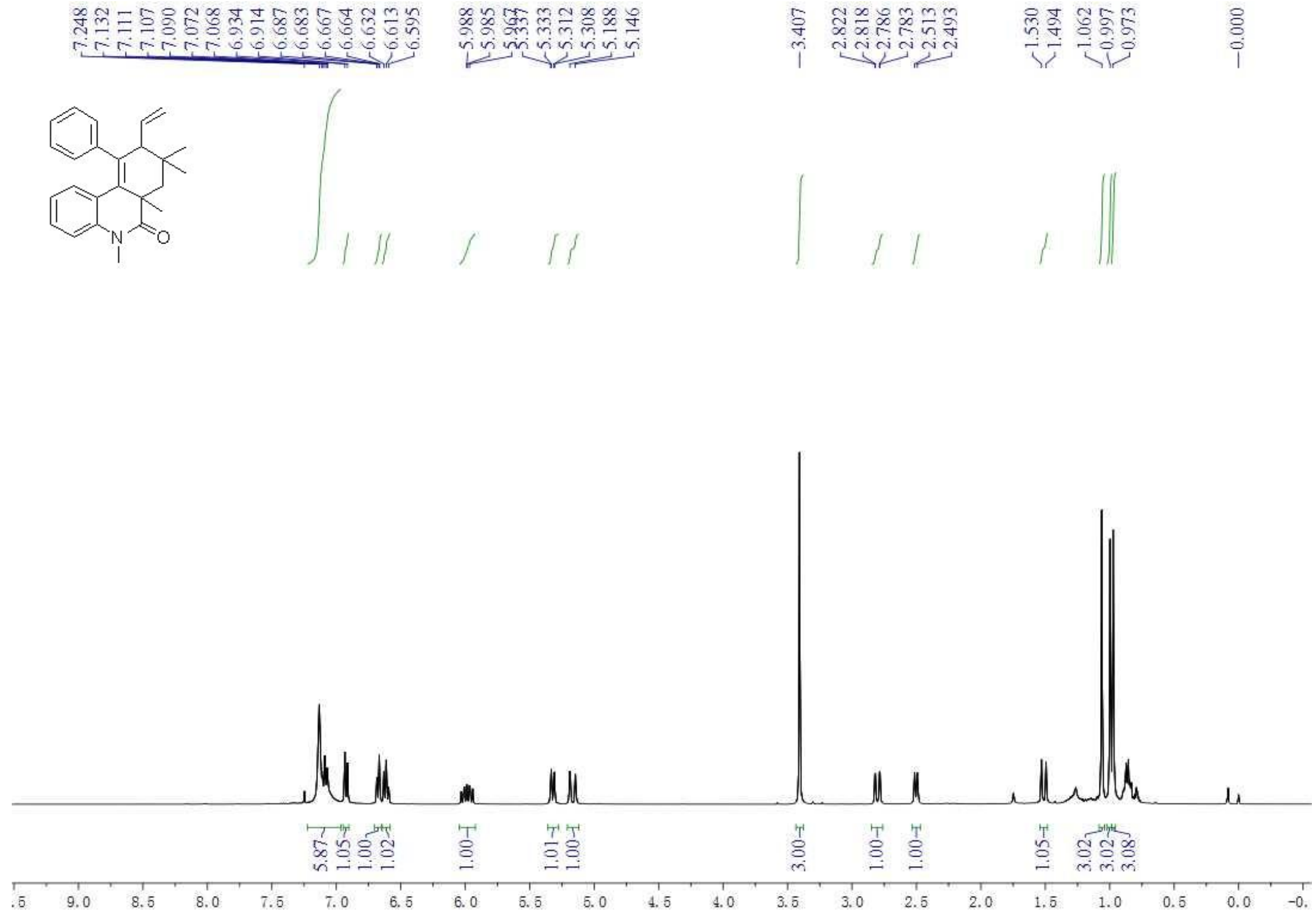

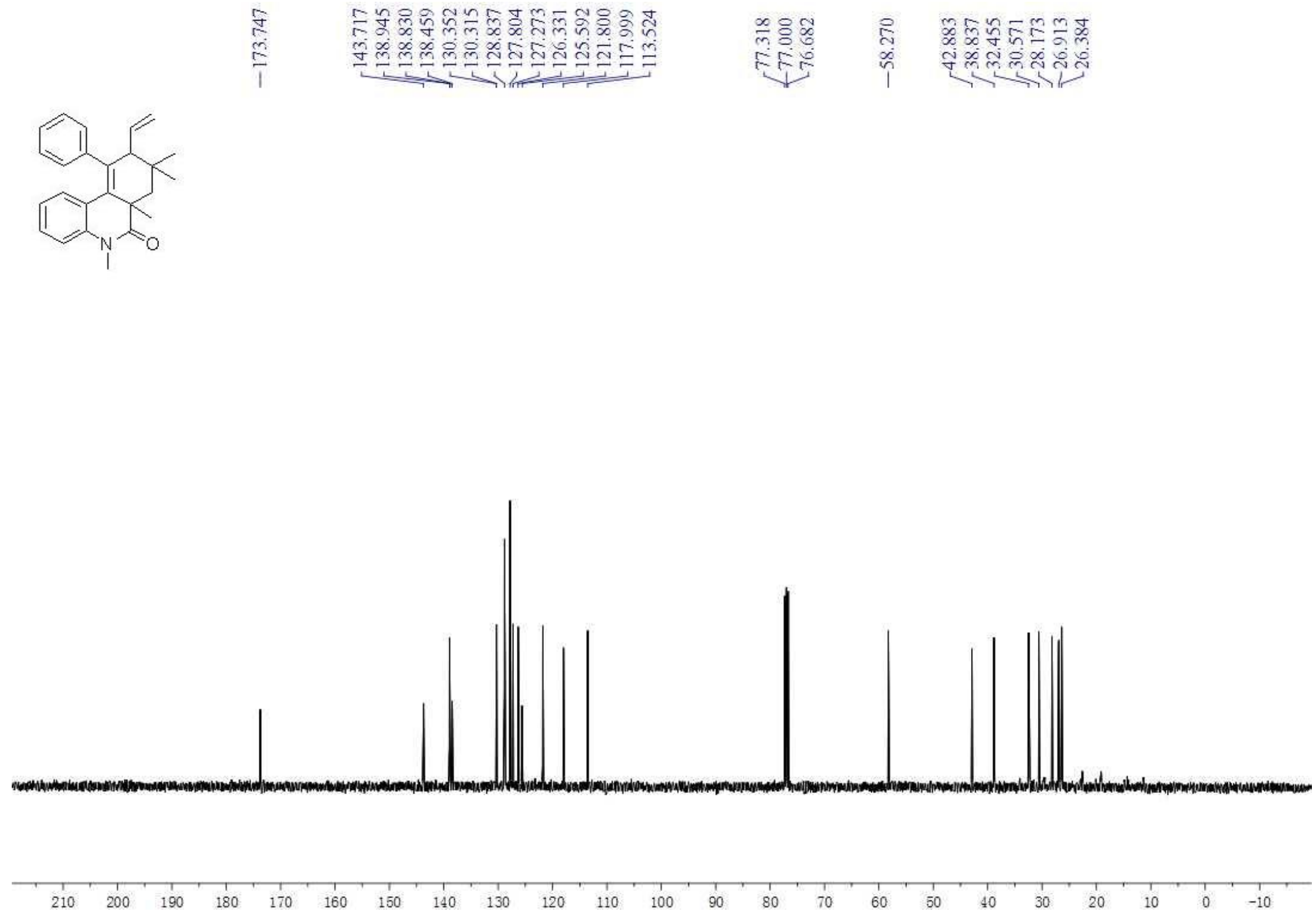

8,8-Diethyl-5,6a-dimethyl-10-(*p*-tolyl)-9-vinyl-6a,7,8,9-tetrahydrophenanthridin-6(5*H*)-one (3fc)

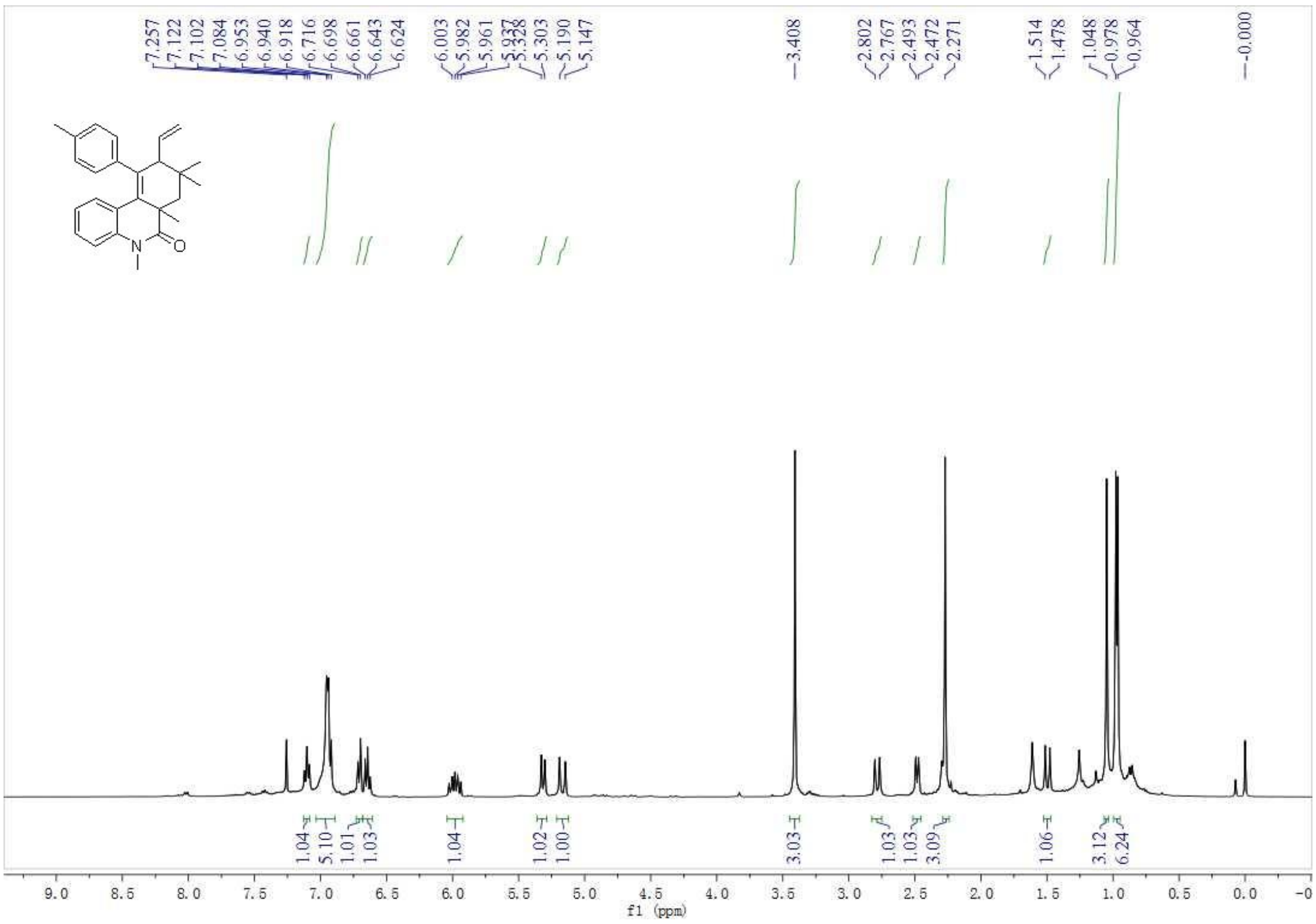

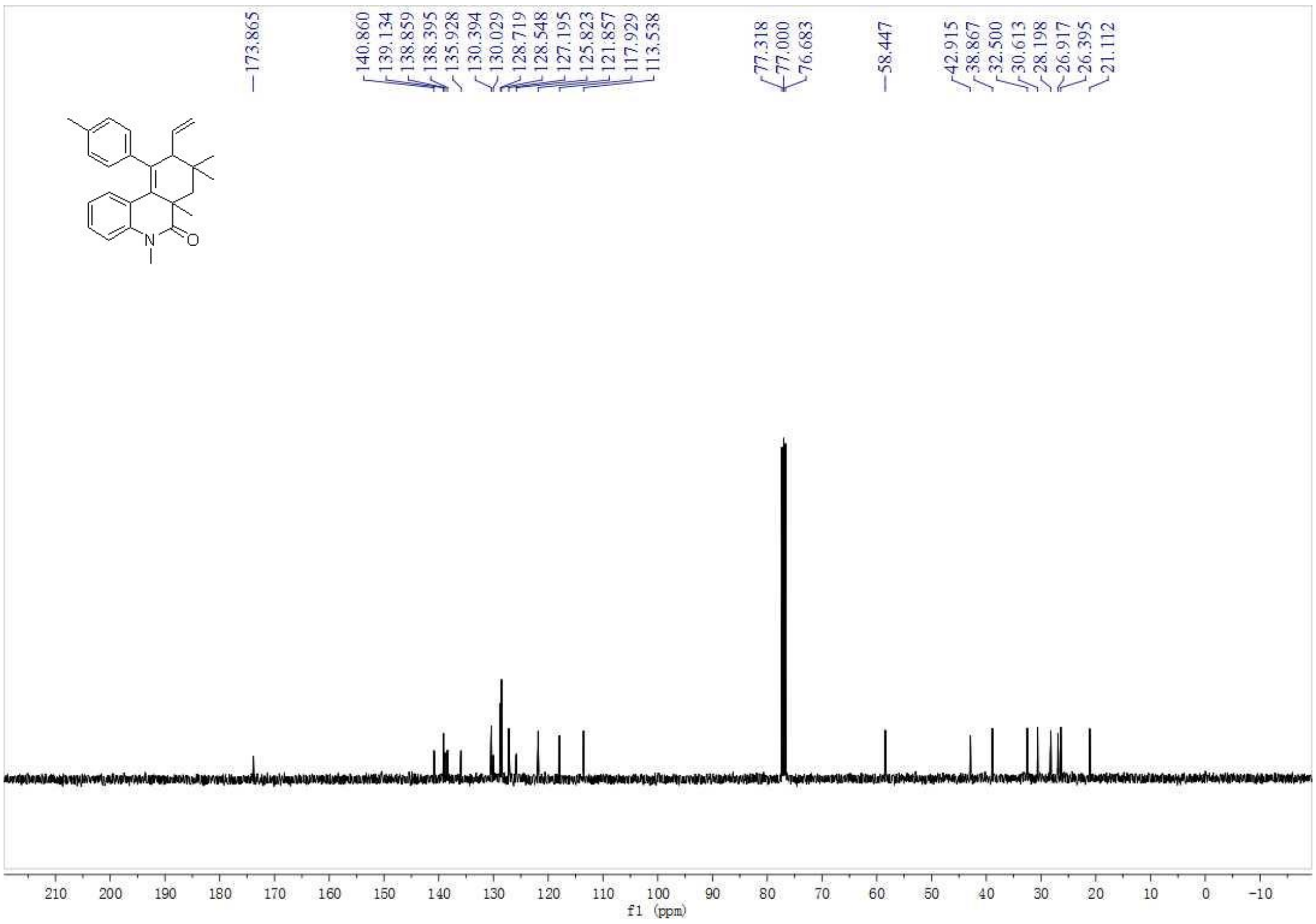

4-(8,8-Diethyl-5,6a-dimethyl-6-oxo-9-vinyl-5,6,6a,7,8,9-hexahydrophenanthridin-10-yl)benzonitrile (3kc)

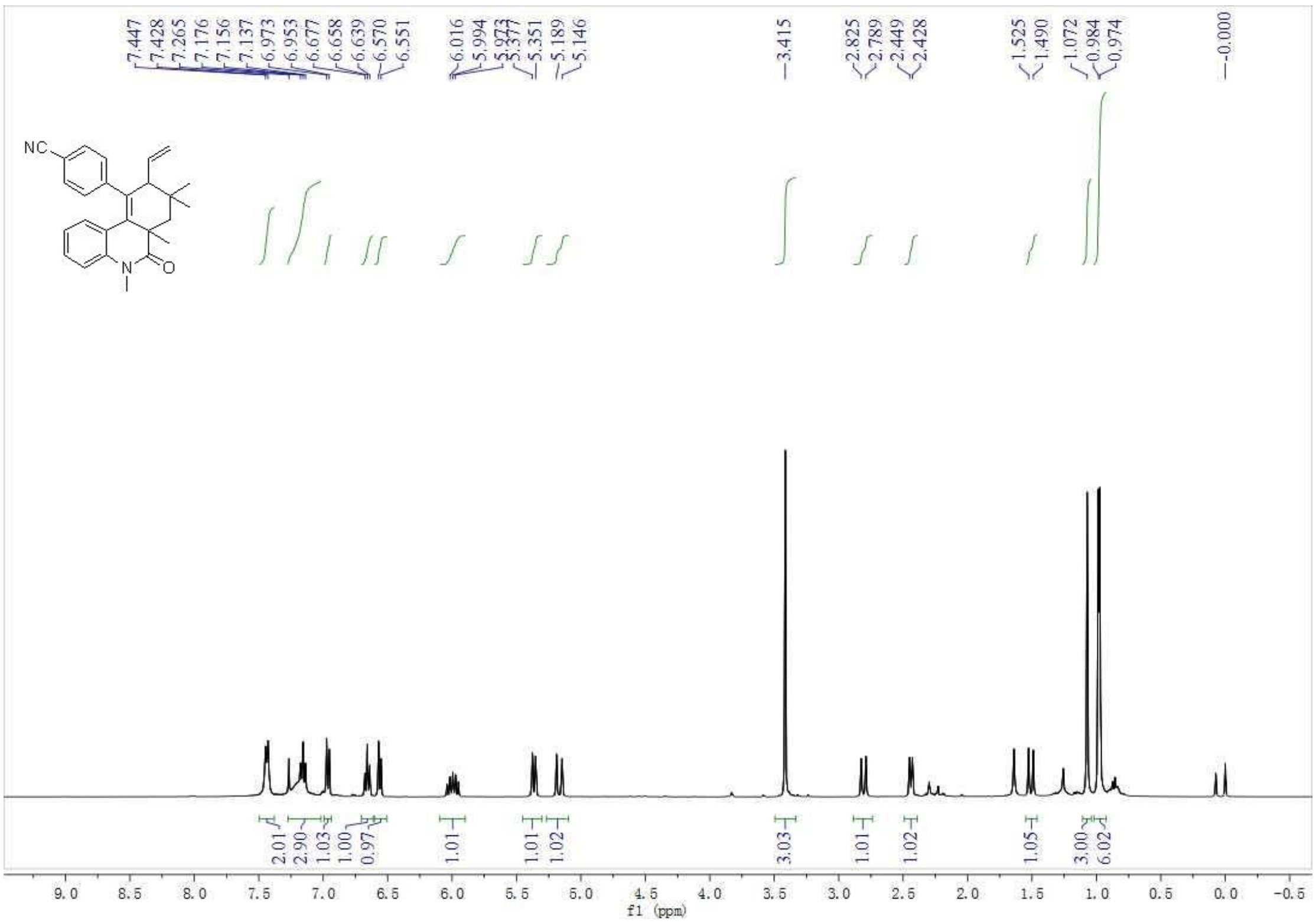

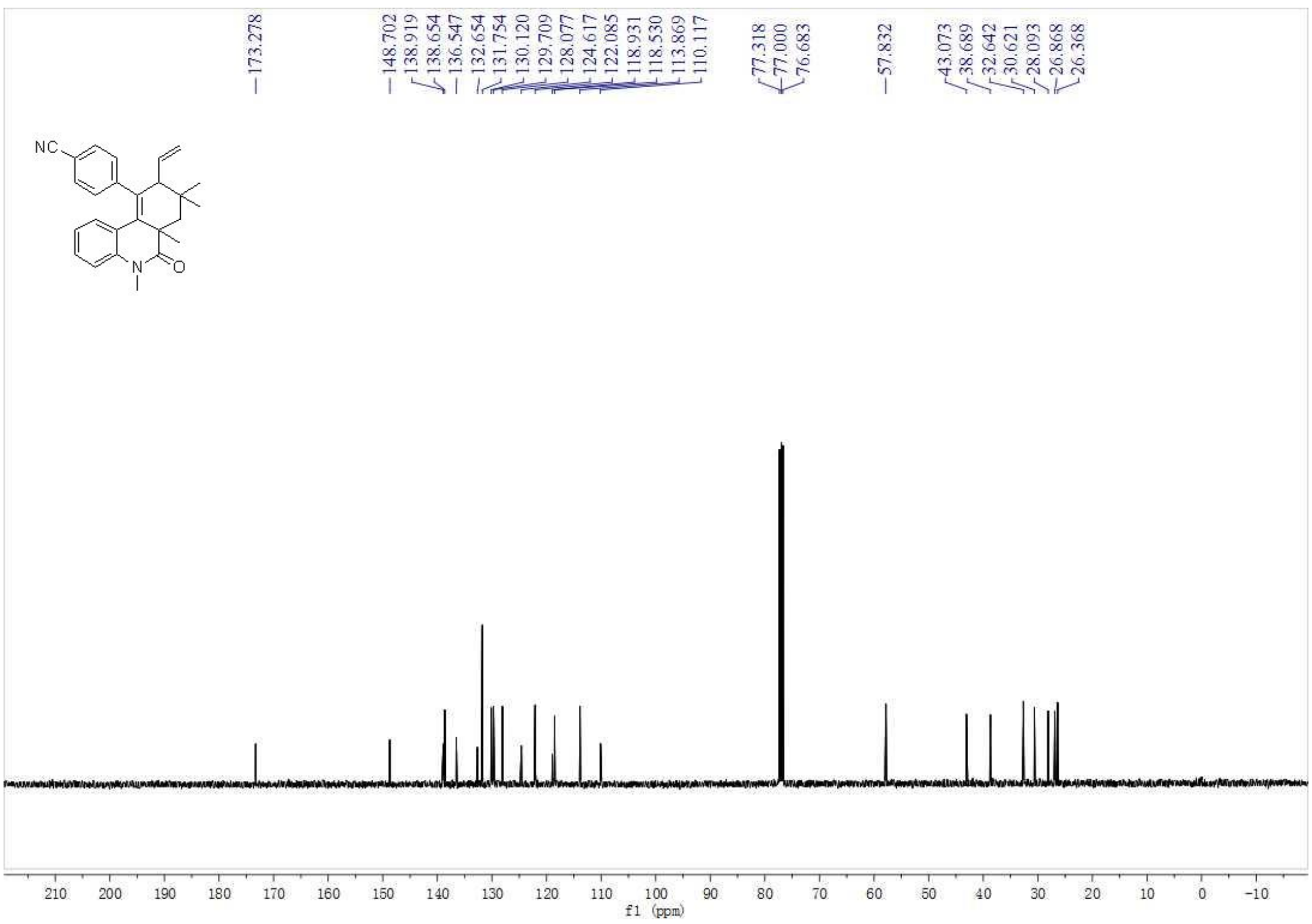

10-Cyclopropyl-8,8-diethyl-5,6a-dimethyl-9-vinyl-6a,7,8,9-tetrahydrophenanthridin-6(5*H*)-one (3tc)

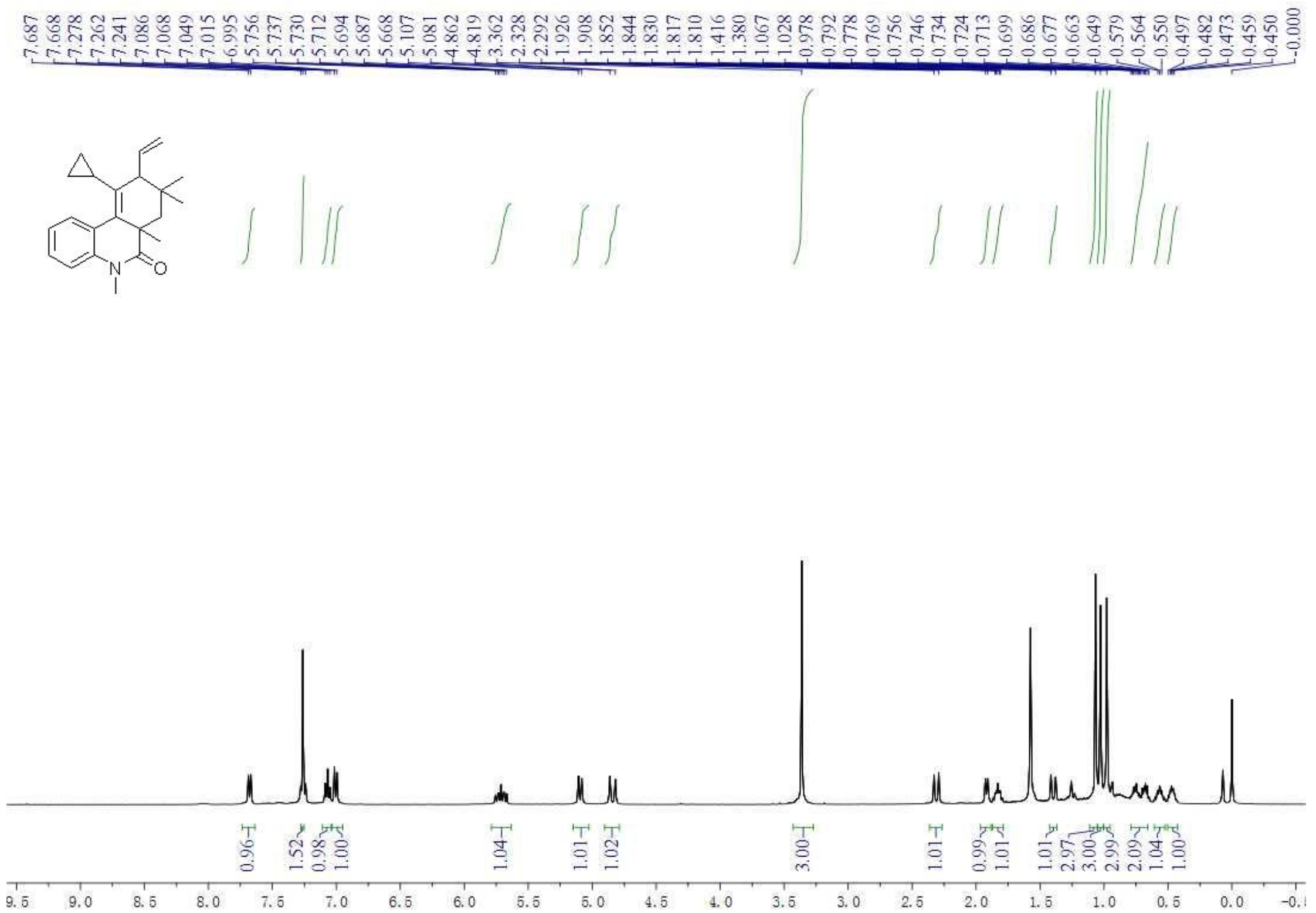

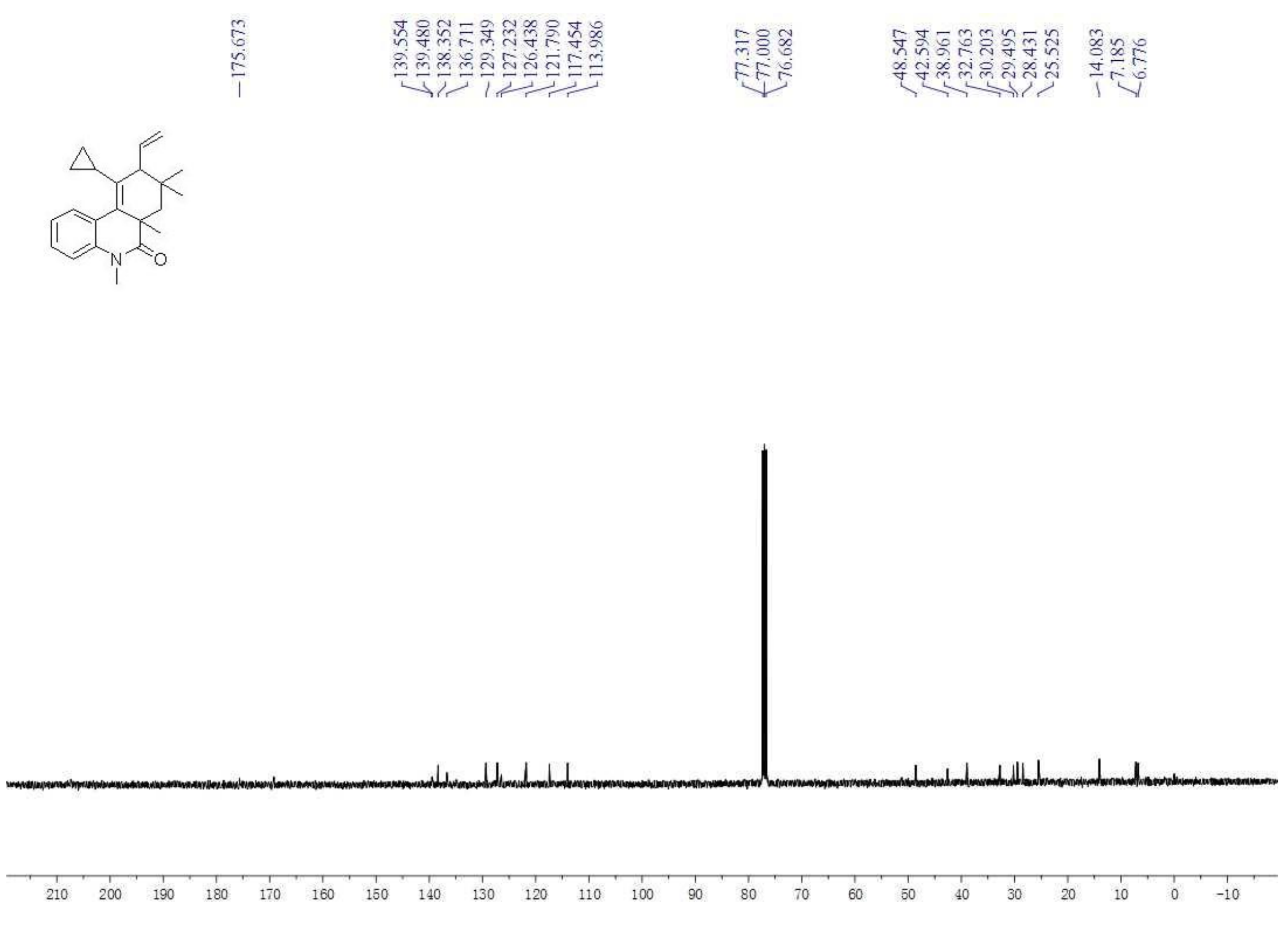

6a,8,8-Trimethyl-10-phenyl-6a,7,8,9-tetrahydro-6*H*-benzo[*c*]chromen-6-one (3ua)

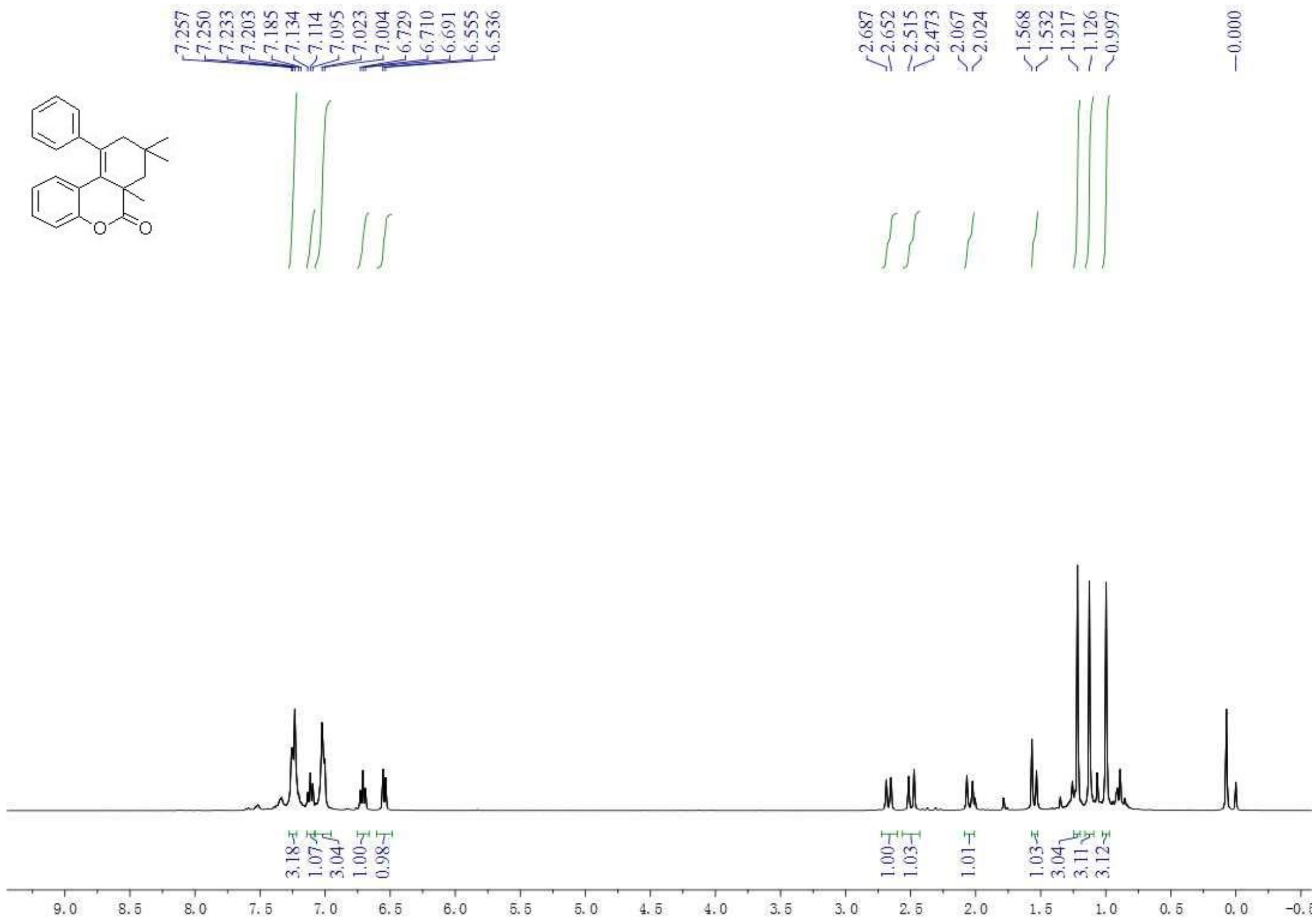

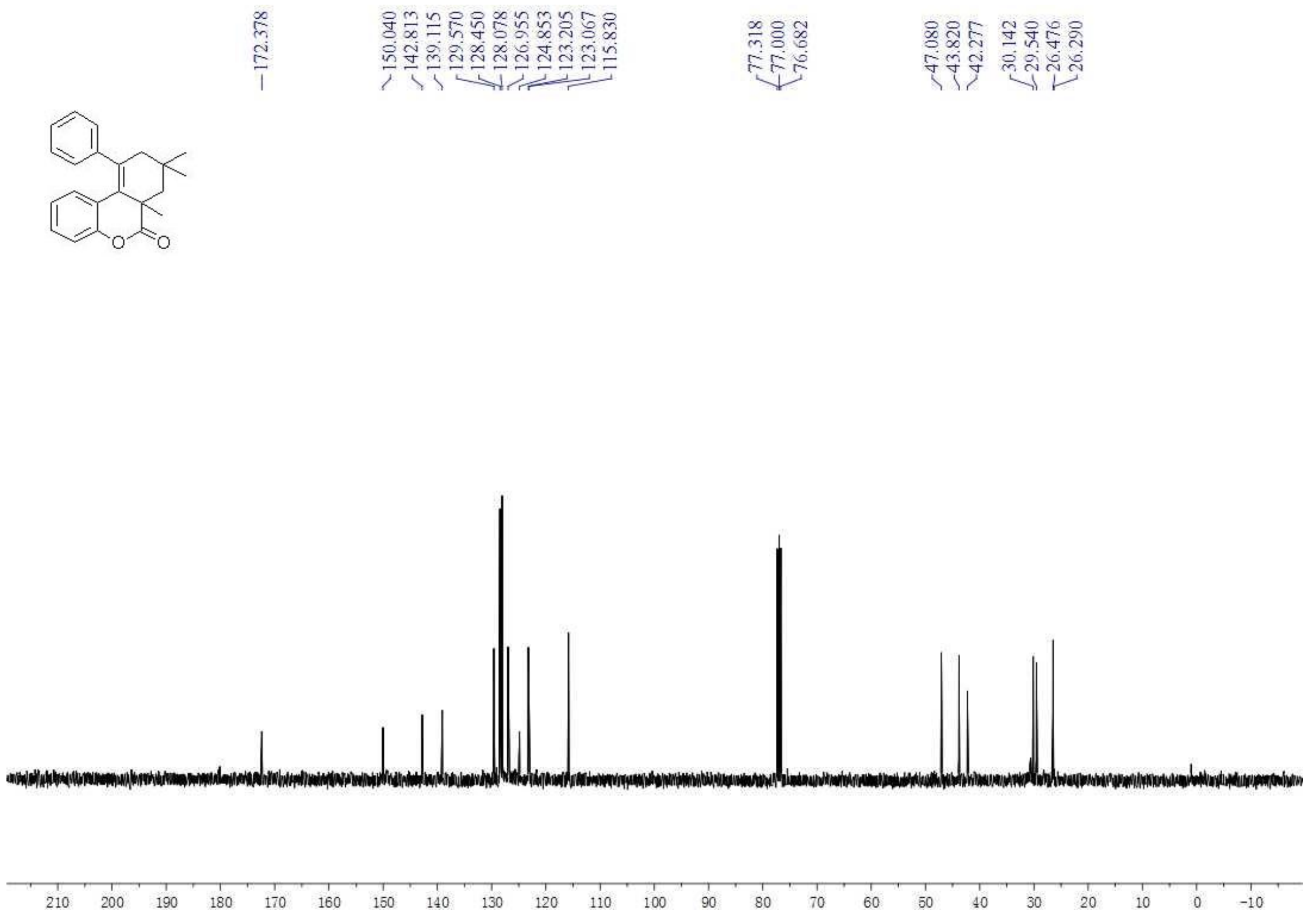

Methyl 7,7-dimethyl-5-phenyl-6,7,8,9-tetrahydro-8aH-fluorene-8a-carboxylate (3va)

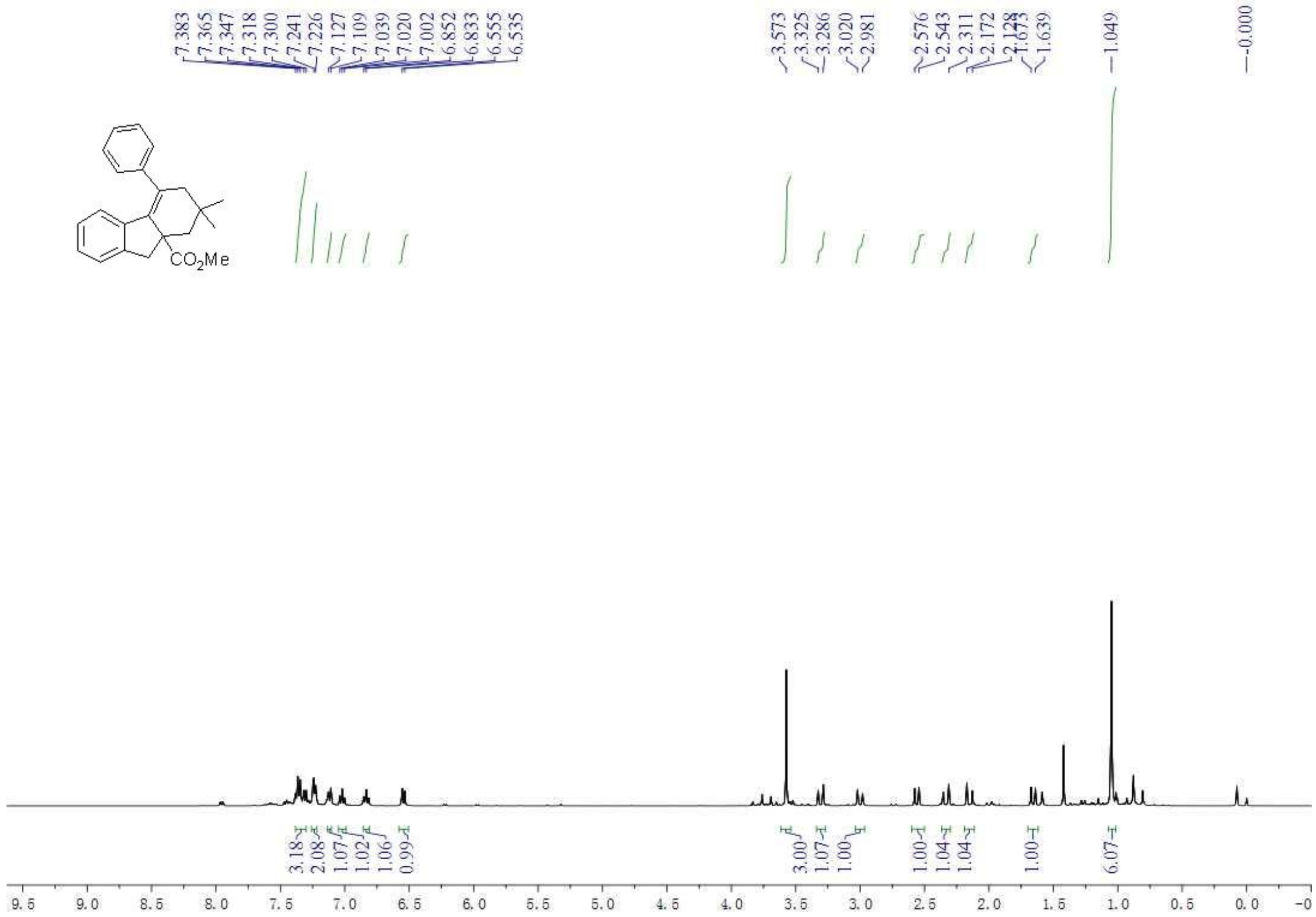

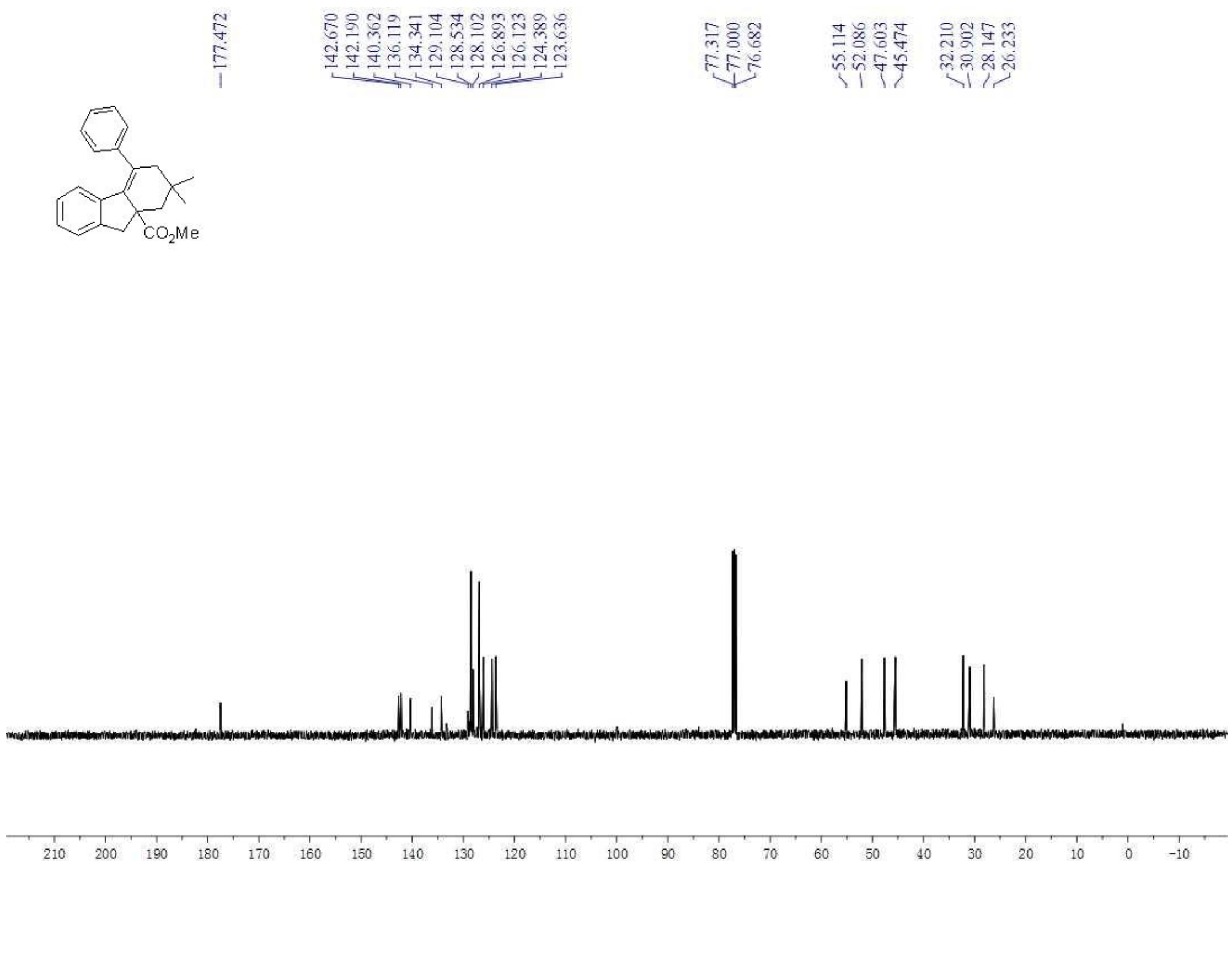

Methyl 7,7-diethyl-5-phenyl-6-vinyl-6,7,8,9-tetrahydro-8a*H*-fluorene-8a-carboxylate (3vc)

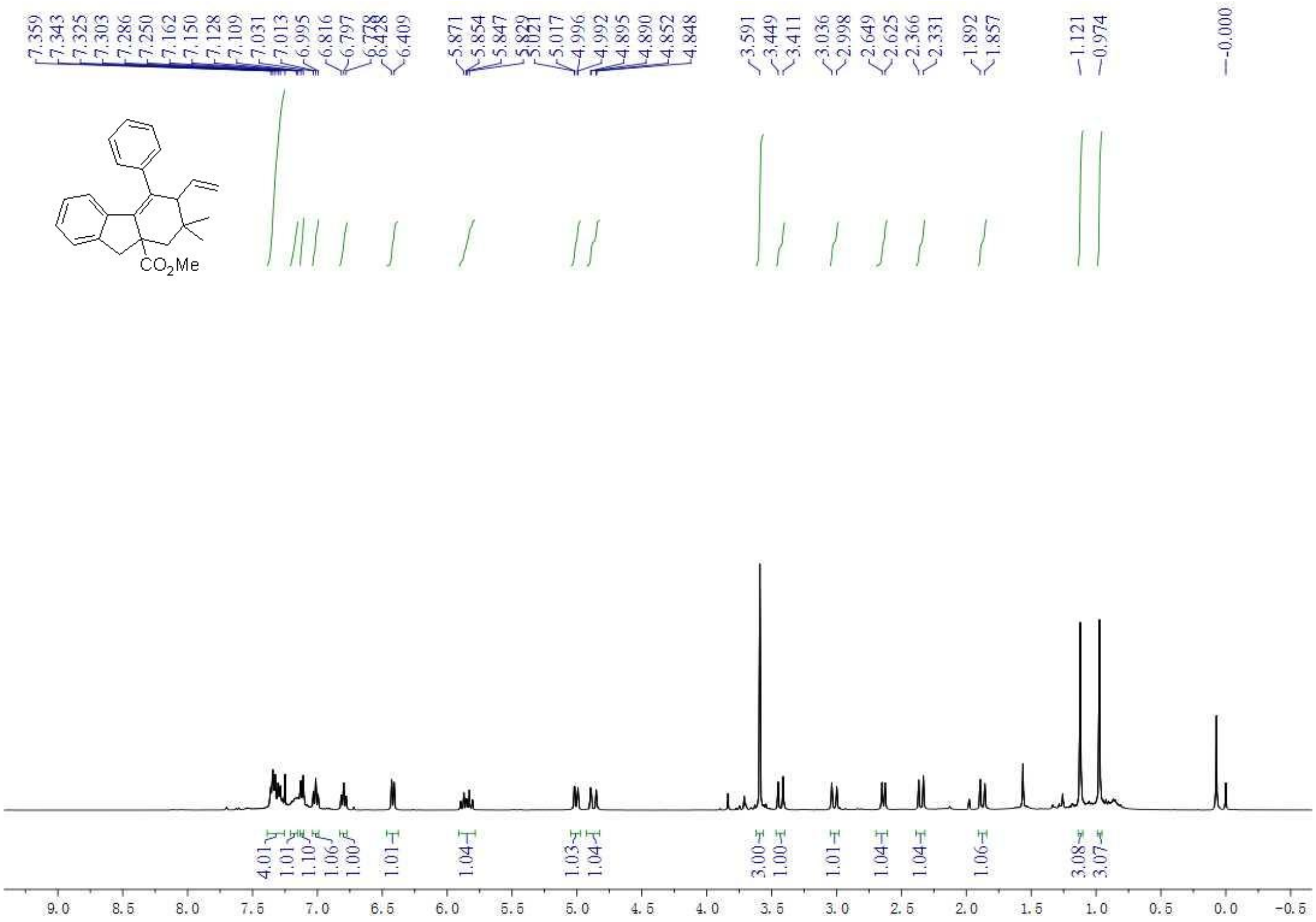

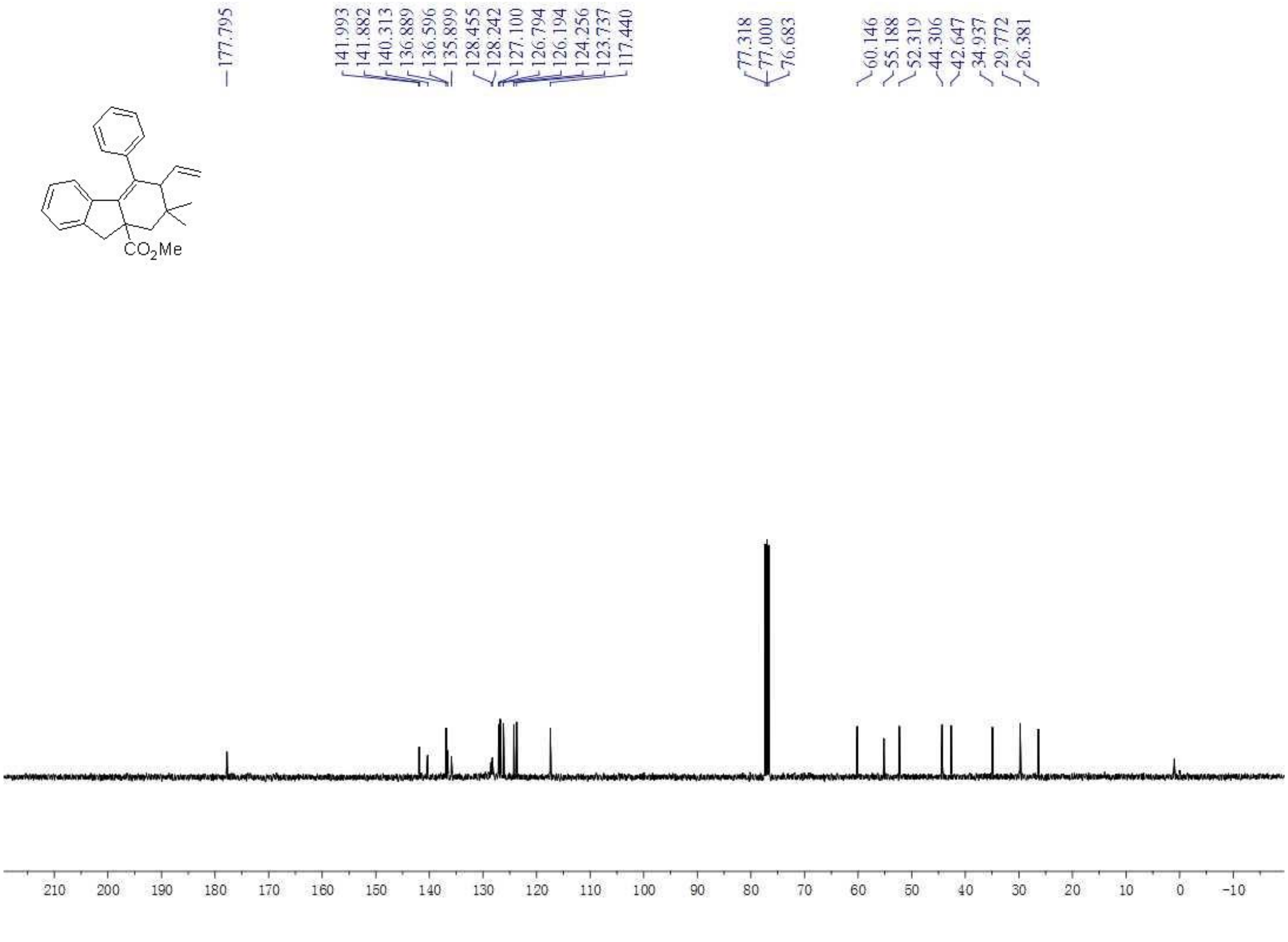

2,2,3a,5-Tetramethyl-1-phenyl-2,3,3a,5-tetrahydro-4*H*-cyclopenta[*c*]quinolin-4-one (4ad)

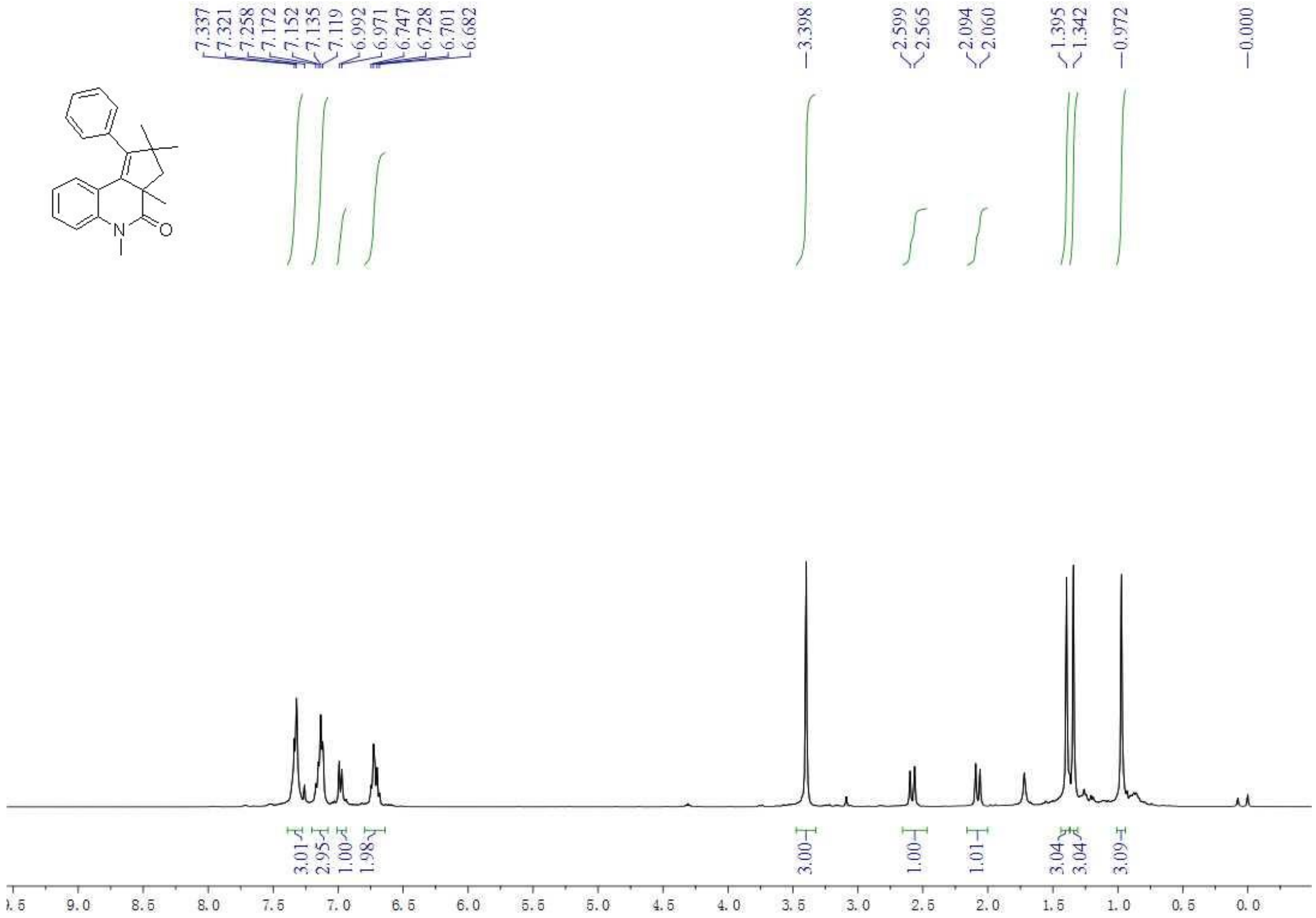

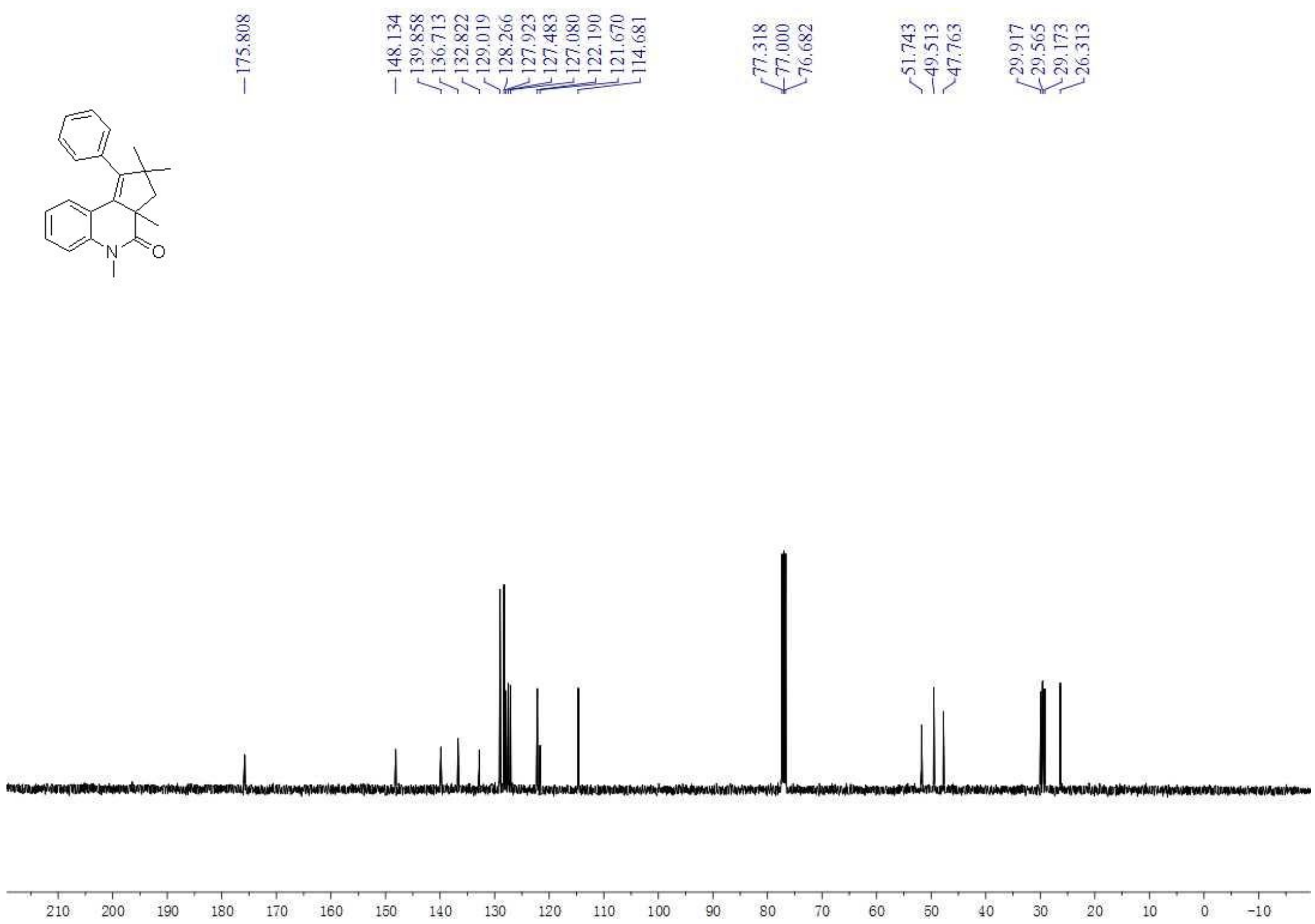

3a',5'-Dimethyl-1'-phenyl-3',3a'-dihydrospiro[cyclopentane-1,2'-cyclopenta[*c*]quinolin]-4'(5'*H*)-one (4ae)

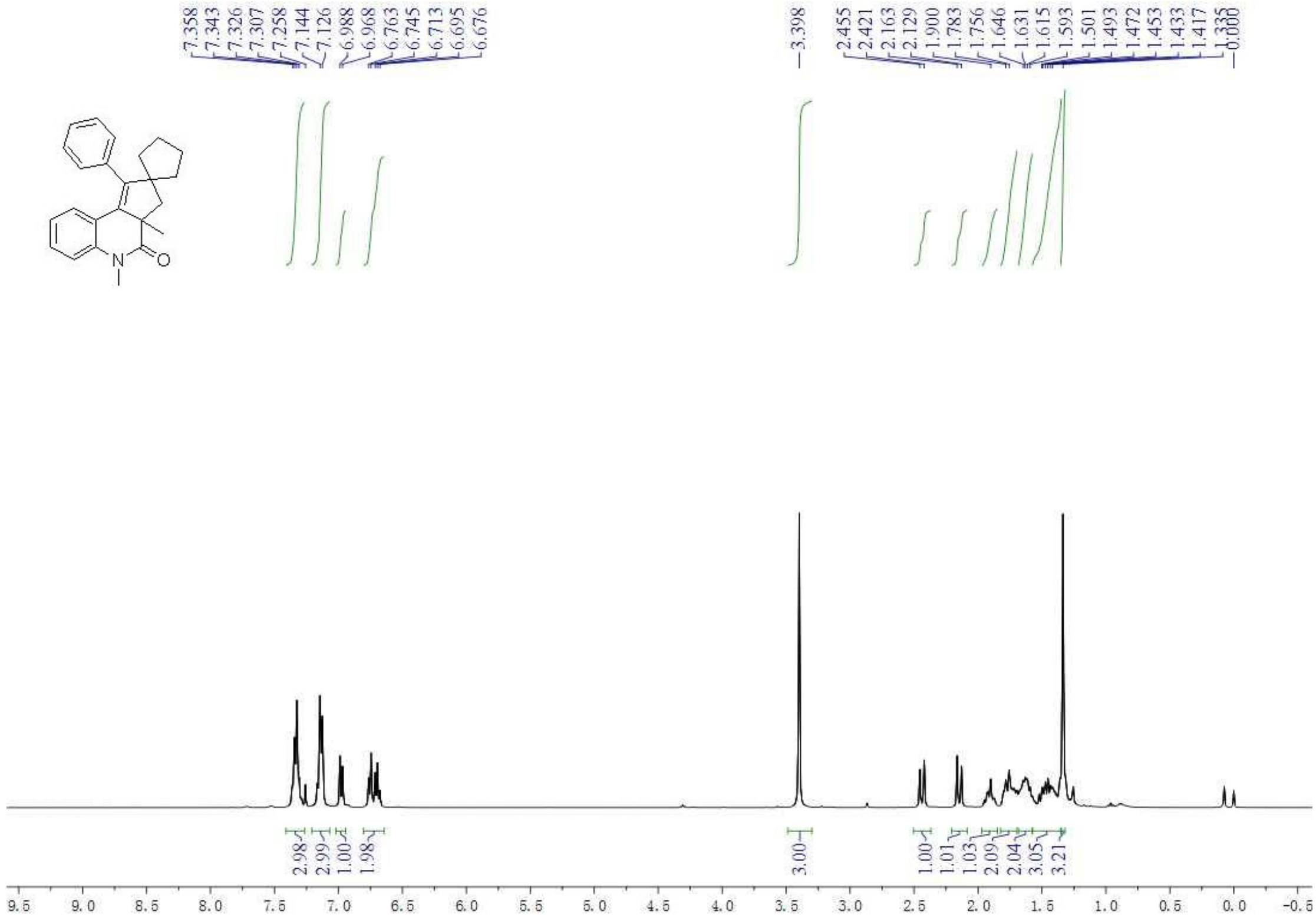

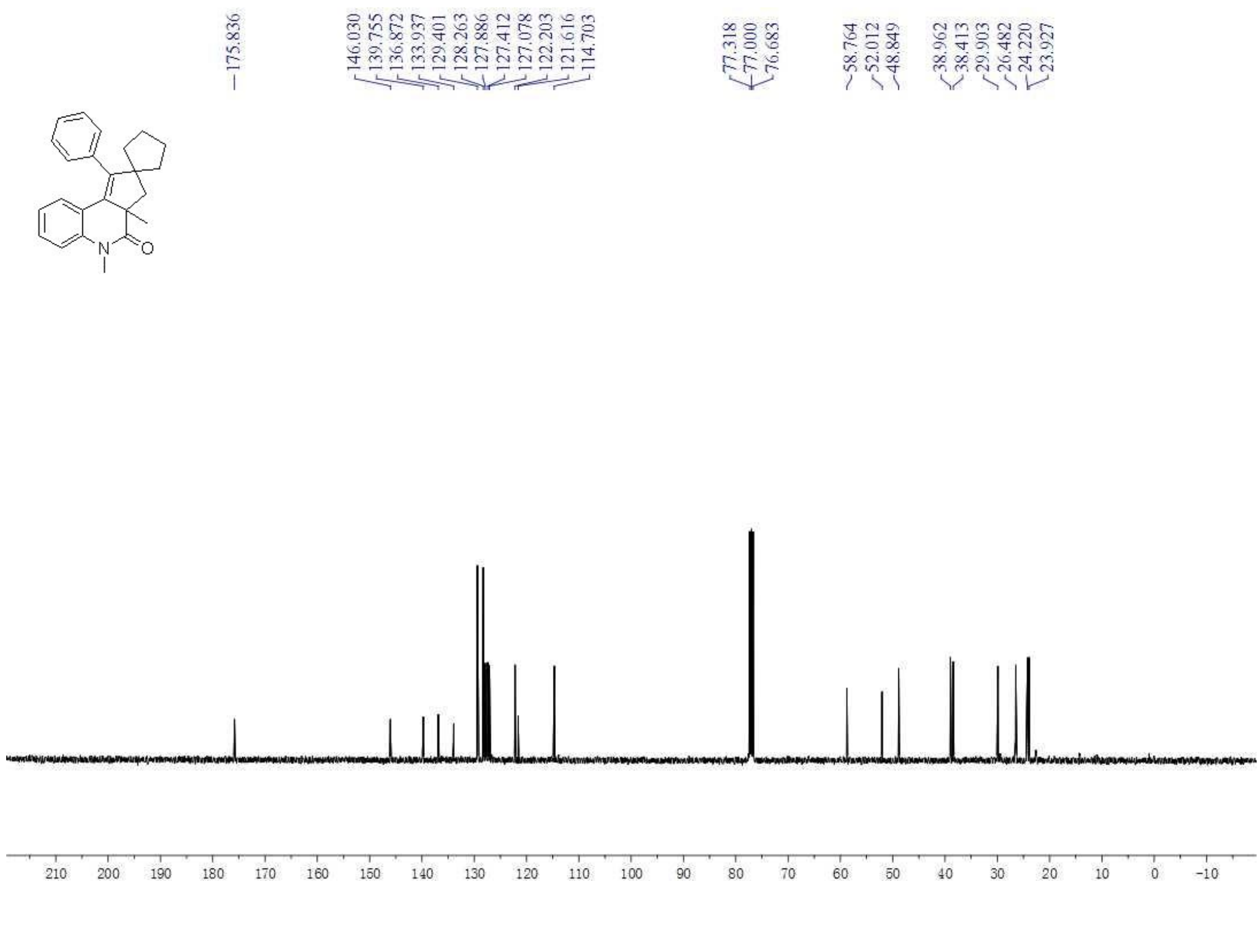

3a',5'-Dimethyl-1'-phenyl-3',3a'-dihydrospiro[cyclohexane-1,2'-cyclopenta[*c*]quinolin]-4'(5'*H*)-one (4af)

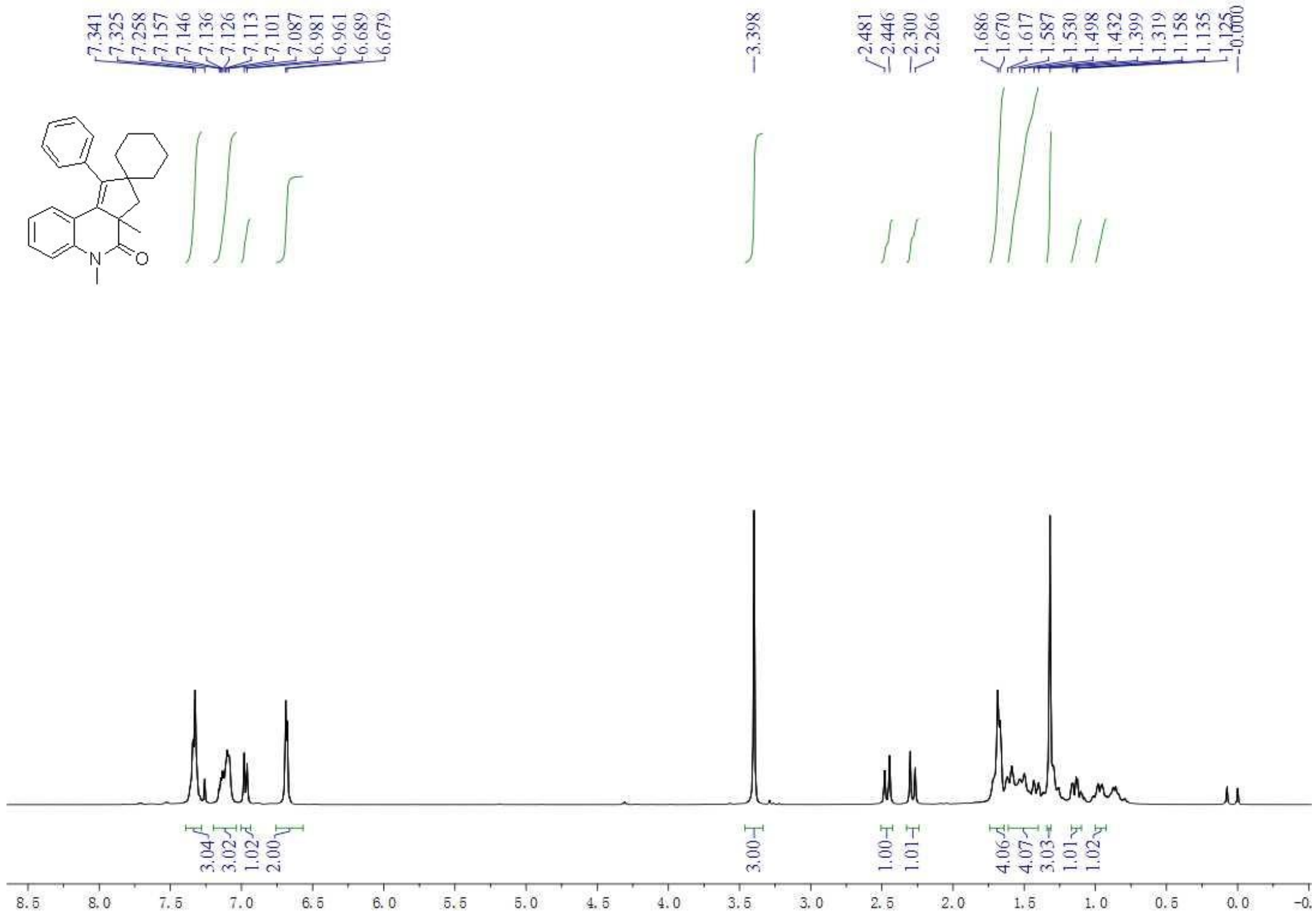

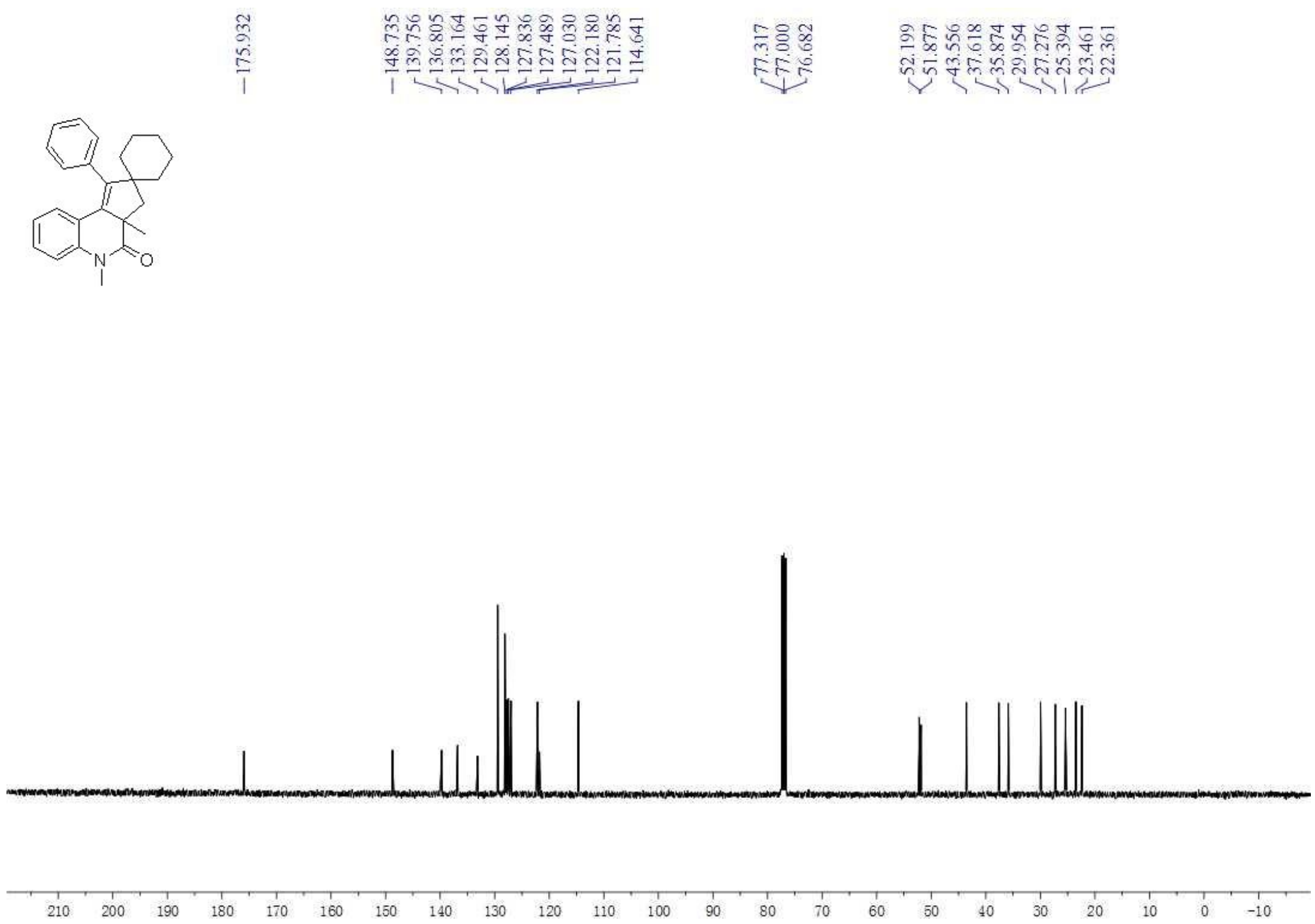

3A,5-dimethyl-1-phenyl-3,3a-dihydro-2*H*-cyclopenta[*c*]quinoline-2,4(5*H*)-dione

(5)

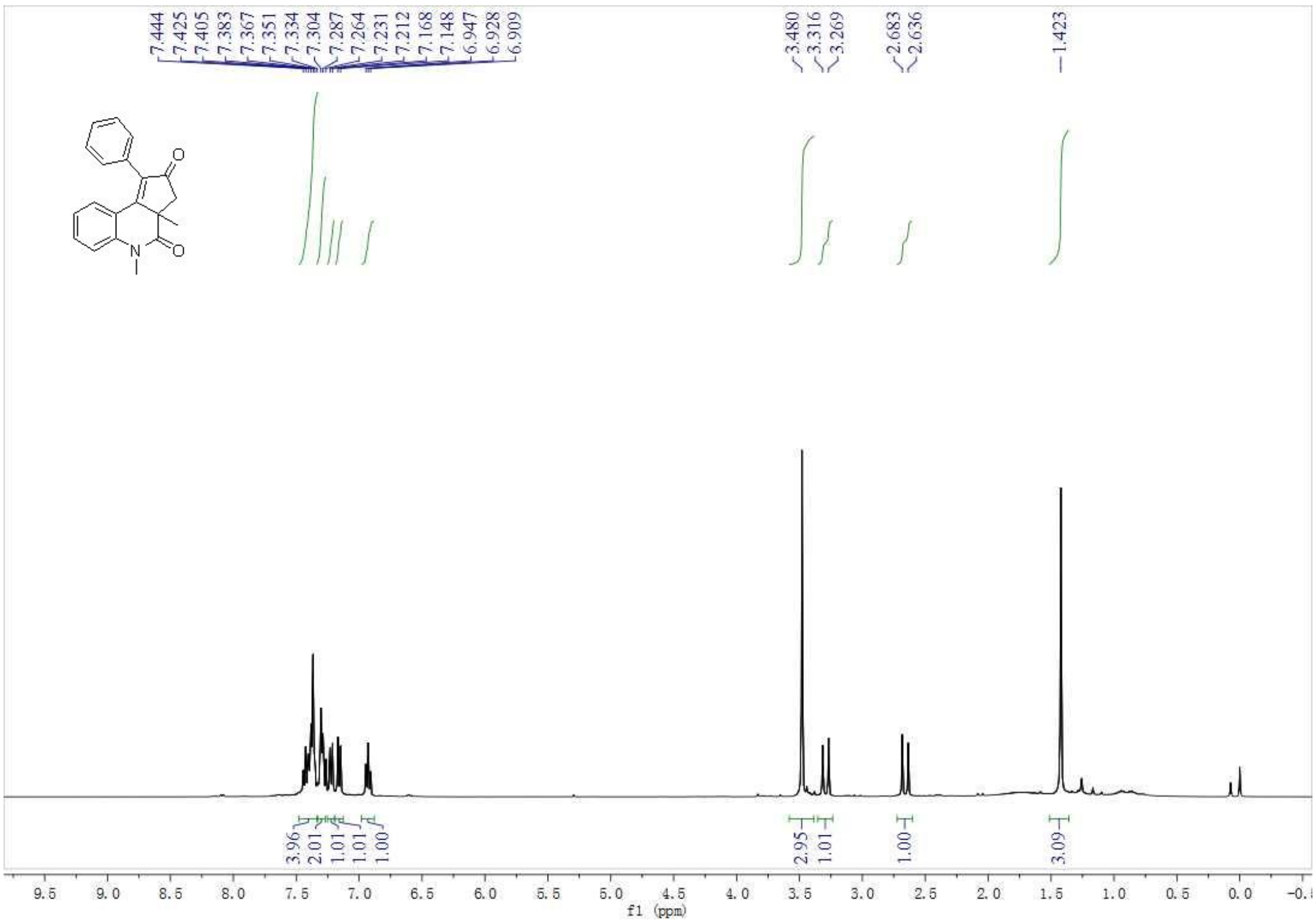

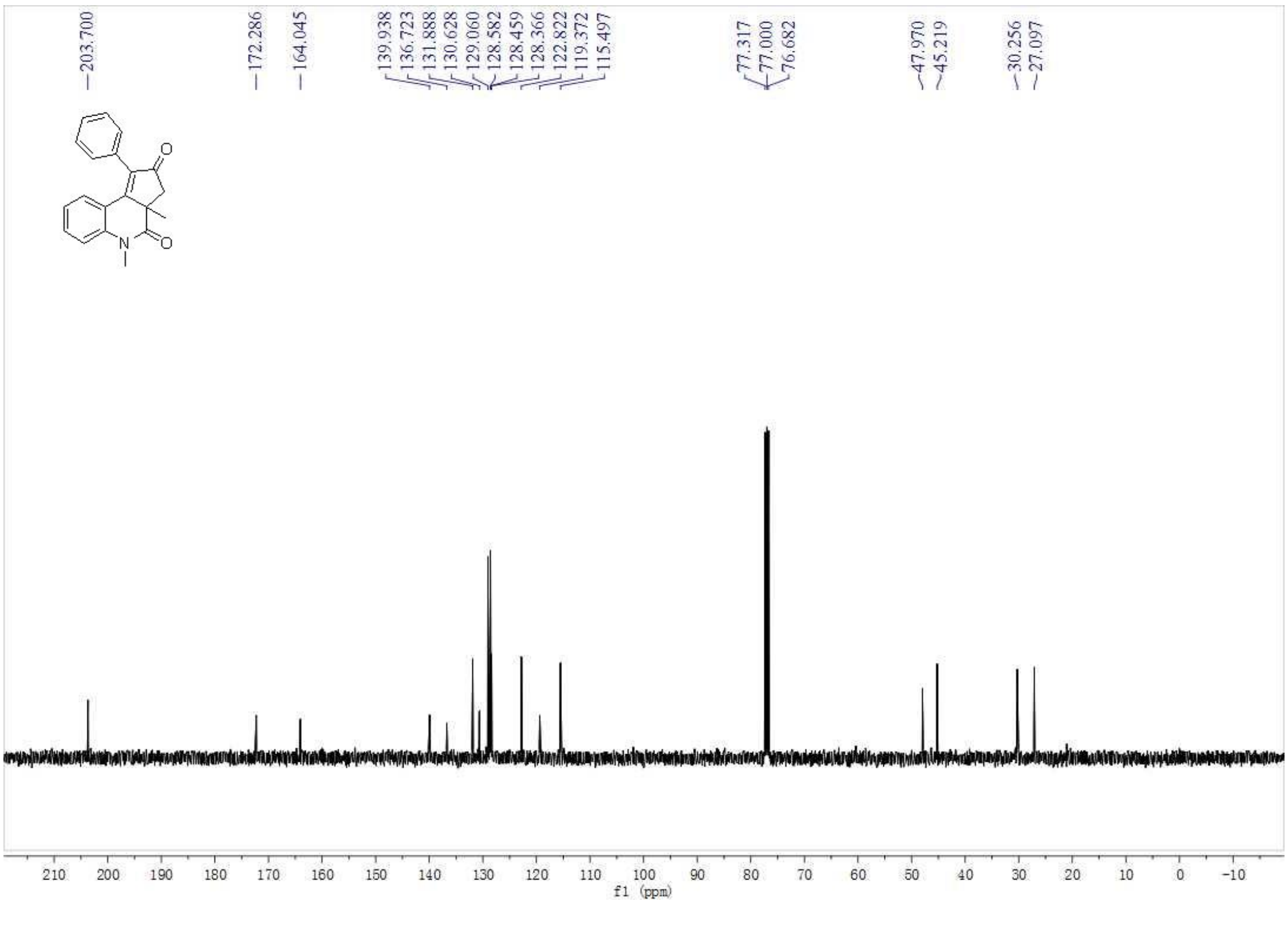

1-(*tert*-Butoxy)-2,2,6,6-tetramethylpiperidine (6)

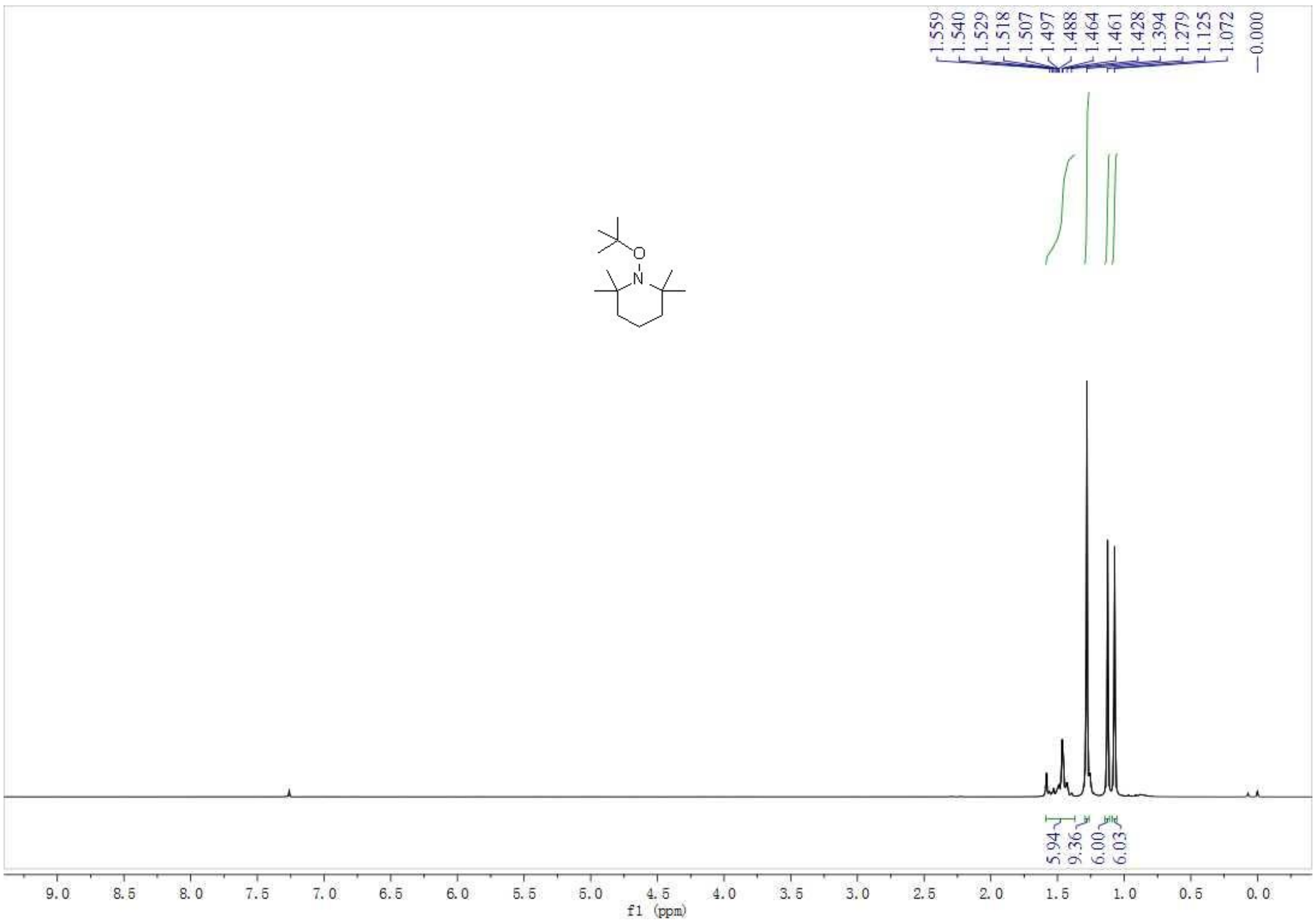

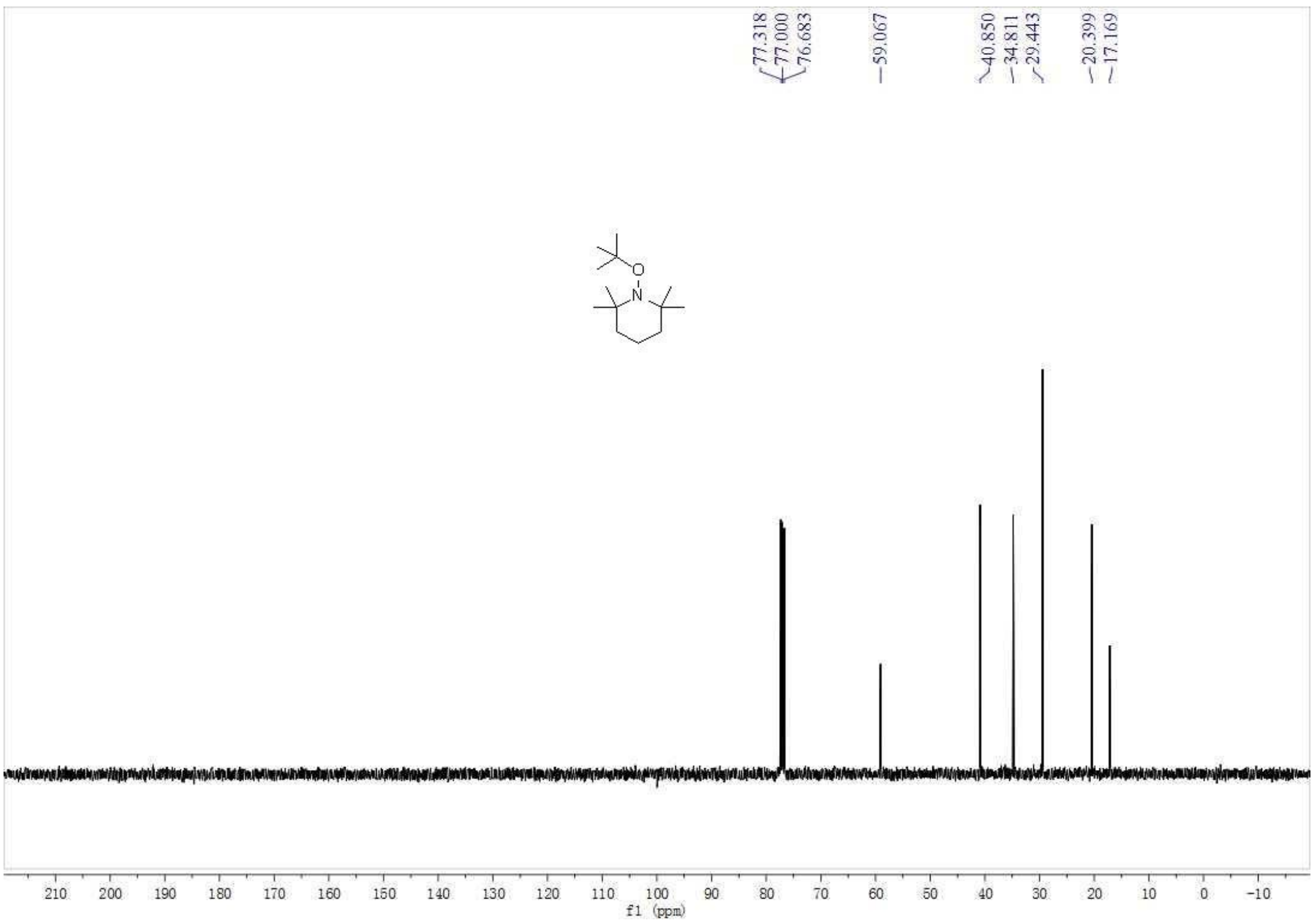

5-Methyl-12-phenylbenzo[*j*]phenanthridin-6(5*H*)-one (7)

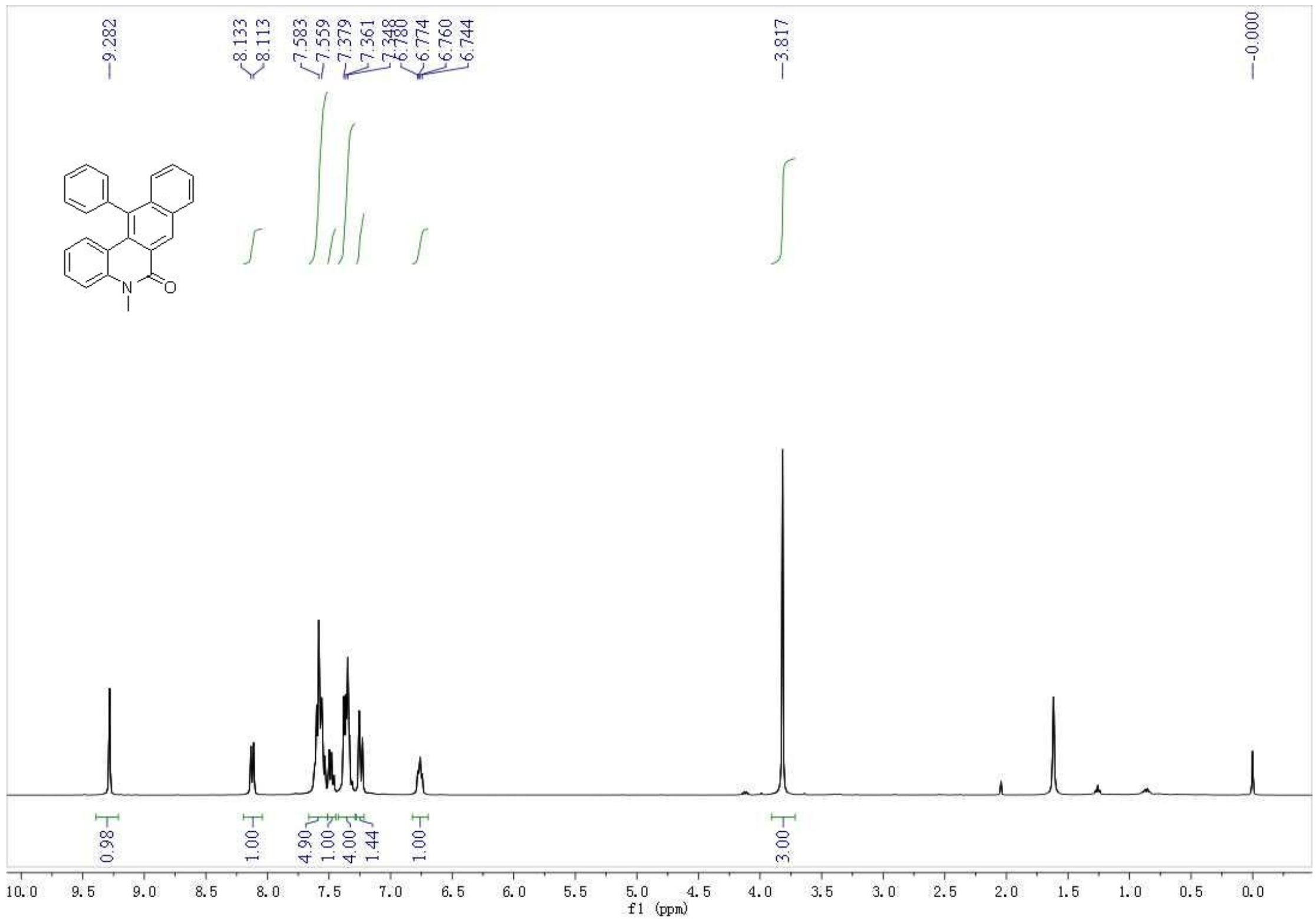

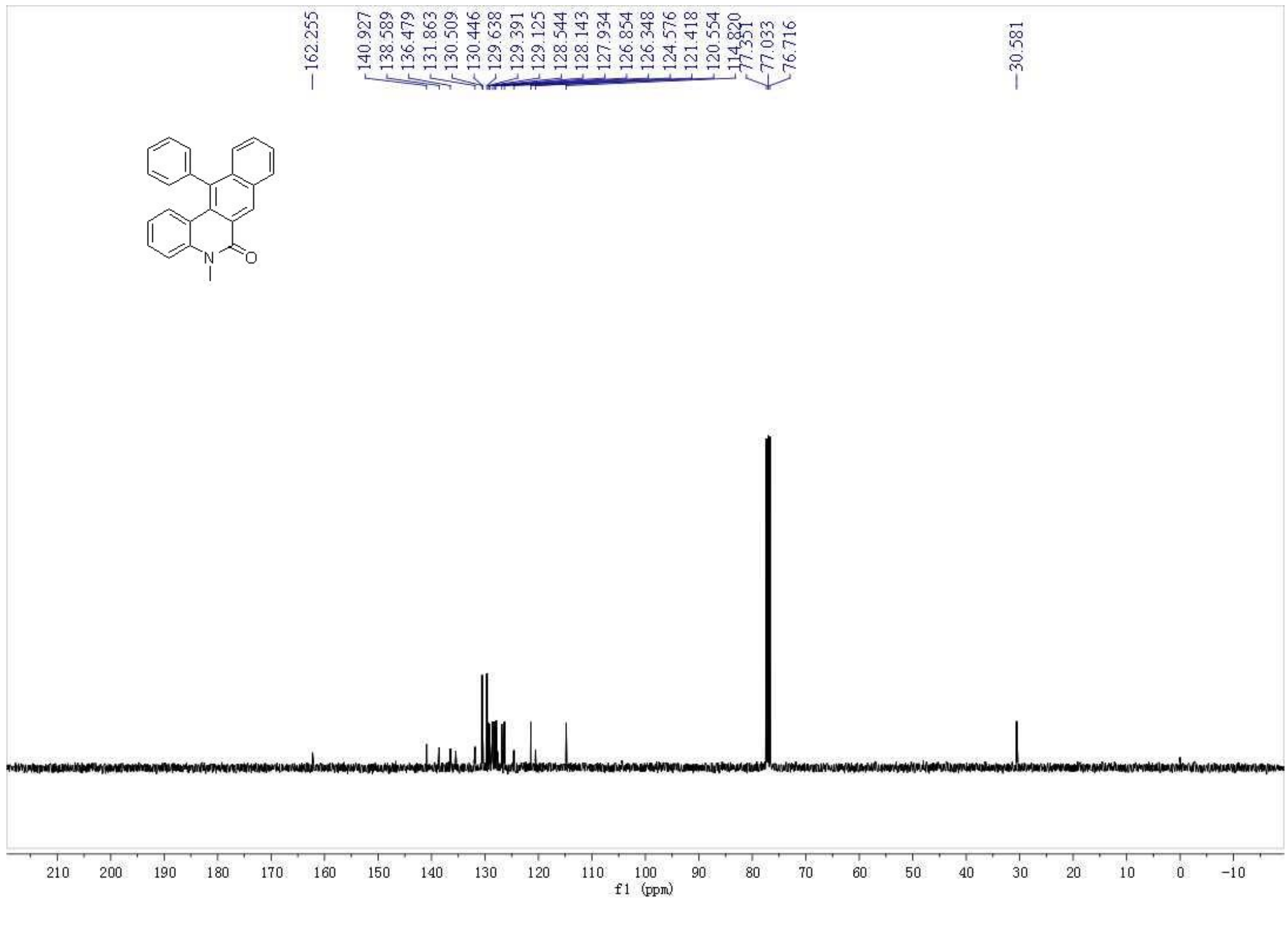

(F) The X-ray single-crystal diffraction analysis of 3ha

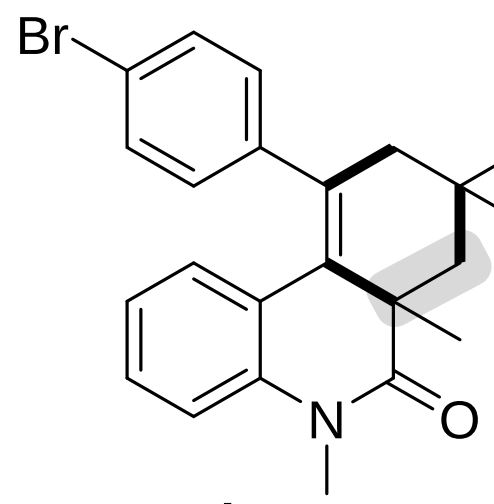

3ha

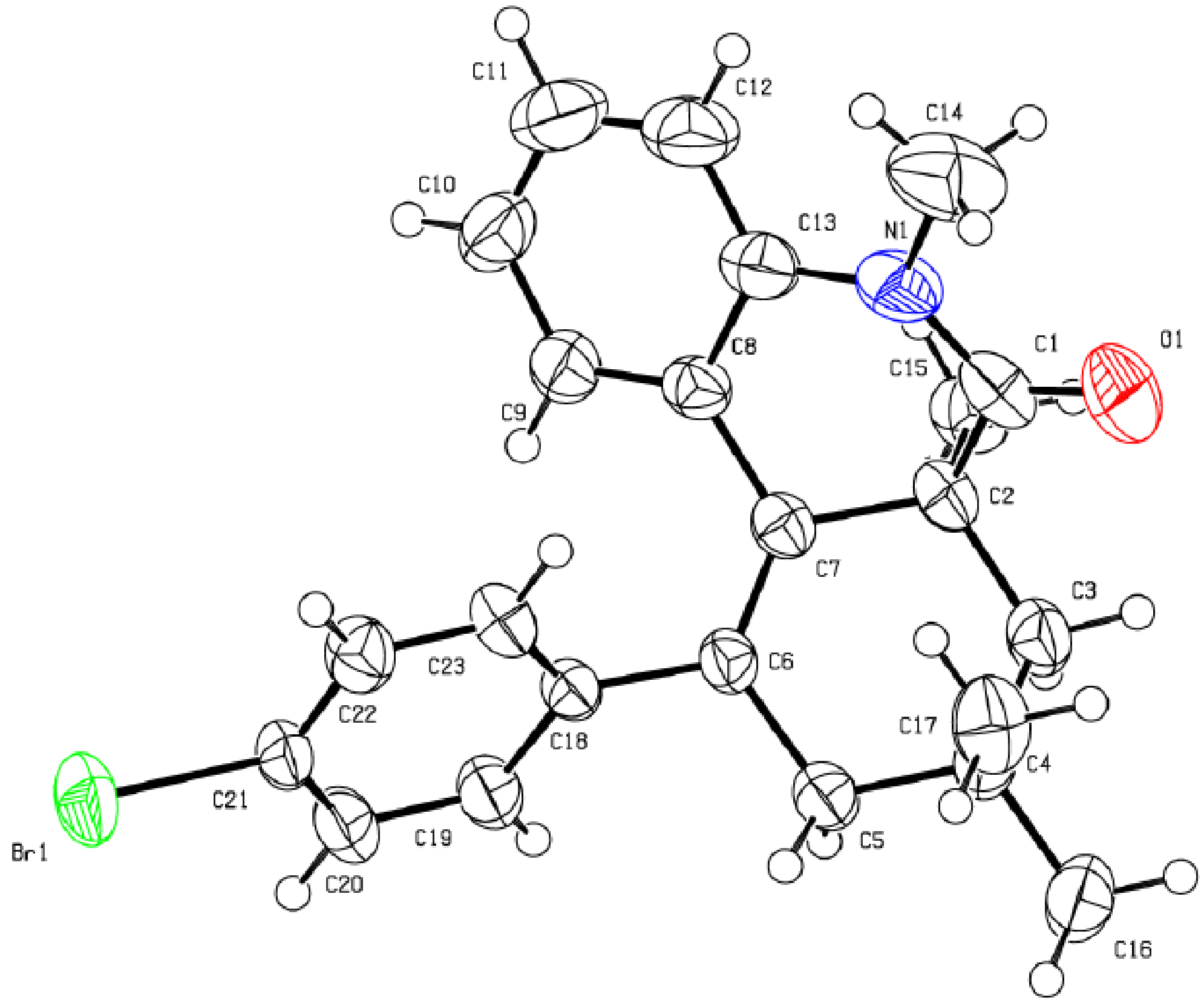

Supplement: Supplementary file 1 [file SC-007-C6SC02451C-s001.pdf]
